# Pyrrolidine Alkaloids from Mangrove Fungus *Penicillium* sp. DM27 Enhance L6 Cell Glucose Uptake

**DOI:** 10.3390/md23120455

**Published:** 2025-11-27

**Authors:** Feng-Kai Fan, Wen-Ting Zhang, Philomina Panin Edjah, Qing-Qing Tang, Wenqing Huang, Li-Ming He, Ming-Qi Zhou, Cong-Kui Tian, Kong-Kai Zhu, Xinzhou Yang, You-Sheng Cai, Kui Hong, Yuan-Zhen Liu

**Affiliations:** 1 Hubei Key Laboratory of Purification and Application of Plant Anticancer Active Ingredients, School of Chemistry and Life Science, Hubei University of Education, Wuhan 430205, China; apexwinner@163.com (F.-K.F.); 15895909065@163.com (W.-T.Z.); cysh2002@whu.edu.cn (Y.-S.C.); 2 Key Laboratory of Combinatorial Biosynthesis and Drug Discovery, Ministry of Education, School of Pharmaceutical Sciences, Wuhan University, Wuhan 430071, China; edjahphilomina2@gmail.com (P.P.E.); 17340559065@163.com (Q.-Q.T.); heliming1999@whu.edu.cn (L.-M.H.); 13554638501@163.com (M.-Q.Z.); 3 International Cooperation Base for Active Substances in Traditional Chinese Medicine in Hubei Province, School of Pharmaceutical Sciences, South-Central Minzu University, Wuhan 430074, China; 18325161433@163.com (W.H.); xzyang@mail.scuec.edu.cn (X.Y.); 4 Hubei Key Laboratory of Selenium Resources Research and Biological Applications, Institute of Selenium Science and Industry, Hubei Minzu University, Enshi 445000, China; pkutianck@163.com; 5 Advanced Medical Research Institute, Shandong University, Jinan 250012, China; kkzhu@sdu.edu.cn

**Keywords:** *Penicillium* sp., pyrrolidine, pyrrolizidine alkaloids, glucose uptake capacity

## Abstract

Ten previously undescribed pyrrolidine alkaloids, namely penicipyrrolidines O–X (**1**–**10**), were isolated from the mangrove-derived fungus *Penicillium* sp. DM27, along with five known compounds (**11**–**15**). Their structures were determined by comprehensive analysis of HRESIMS and NMR spectroscopic data, and the absolute configurations were established based on biosynthetic considerations and TDDFT-ECD calculations. All isolates were evaluated for their glucose uptake capacity. Notably, penicipyrrolidine P (**2**) significantly enhanced cellular glucose uptake in L6 myotubes by 3.83-fold, demonstrating activity comparable to that of metformin, whereas penicipyrrolidines Q and R (**3** and **4**) showed relatively weaker effects.

## 1. Introduction

Mangrove-associated fungi are a prolific source of diverse bioactive metabolites. Endophytic fungi, once poorly documented in mangrove roots [1], have emerged as promising sources of complex compounds with antimicrobial, antioxidant, and enzymatic activities [2,3,4,5,6,7,8]. Fungi belonging to the genus *Penicillium* are especially recognized as producers of structurally unique and biologically active metabolites [9]. Notably, natural products containing pyrrolidine core exhibit a broad spectrum of pharmacological activities, such as antimicrobial, anticancer, and enzyme inhibitory effects [10]. Nevertheless, only a limited number of pyrrolidine derivatives containing the distinctive 2-methyl-1-(pyrrolidinyl)-decanone scaffold have been reported from marine-derived *Penicillium* species [11,12,13,14,15].

Our prior research on the mangrove-derived fungus *Penicillium* sp. DM27 led to the isolation and characterization of nineteen novel pyrrolidine alkaloids (penicipyrrolidines A–N) and three pyrrolizidinone alkaloids, penicipyrrolizidinones A–C, with unprecedented skeletons [16,17,18]. While penicipyrrolidine K demonstrated potent anti-fibrotic activity by targeting ADAM17, and two compounds exhibited moderate cytotoxicity, the overall exploration of biological activities for these structurally diverse pyrrolidine derivatives from the strain DM27 remains limited [16,17].

**Figure 1:**
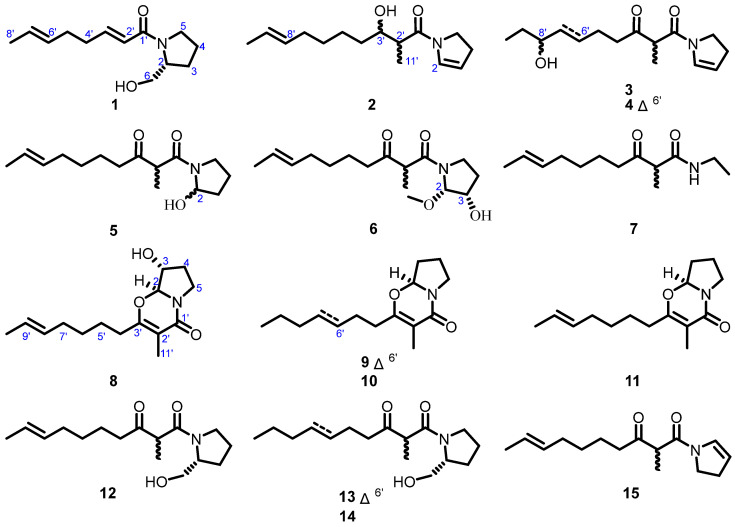
Structures of compounds **1**–**15**.

Motivated by the structural richness and untapped bioactivity potential of pyrrolidine alkaloids from the title fungus, we turned our attention to diabetes mellitus, a widespread metabolic disorder marked by progressive β-cell dysfunction and hyperglycemia [19,20]. Through large-scale fermentation of *Penicillium* sp. DM27, ten new pyrrolidine alkaloids, designated penicipyrrolidines O–X (**1**–**10**), along with five known analogues (**11**–**15**), were obtained (Figure 1). Notably, penicipyrrolidines P–R (**2**–**4**) exhibited stimulatory effects on glucose uptake, highlighting their potential as antidiabetic agents and prompting further investigation.

## 2. Results and Discussion

### 2.1. Structural Analysis

**Figure 2:**
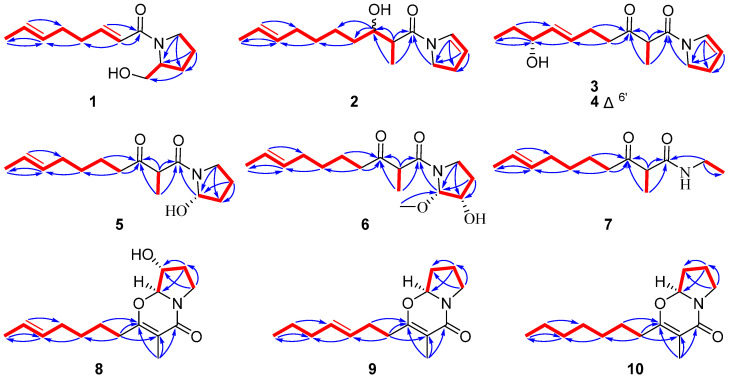
Key ^1^H-^1^H COSY (red bold) and HMBC (blue arrows) correlations of compounds **1**–**10**.

Penicipyrrolidine O (**1**) was isolated as a colorless oil, and assigned a molecular formula of C_13_H_21_NO_2_ (four degrees of unsaturation) based on HRESIMS ions at *m*/*z* [M + Na]^+^ 246.1463 and ^13^C NMR data (Table 1). The ^1^H NMR spectrum of **1** exhibited four olefinic proton signals (*δ*_H_ 6.96, 6.12, 5.46, 5.42) and one methyl at *δ*_H_ 1.65. The ^13^C NMR and HSQC of **1** showed an amide carbonyl (*δ*_C_ 167.6), four olefinic carbon signals (*δ*_C_ 147.1, 129.9, 126.2, 121.6), one oxymethylene (*δ*_C_ 67.9), one methine (*δ*_C_ 61.6), five methylenes (*δ*_C_ 48.2, 32.7, 31.4, 28.5, 24.6), and one methyl (*δ*_C_ 18.1). Four degrees of unsaturation were calculated from its molecular formula, the carbonyl and the two double bonds accounted for three, which suggested a ring in **1**. Comprehensive analysis of the NMR data (Table 1), particularly the characteristic proton resonances at CH-2, CH_2_-3, CH_2_-4, CH_2_-5 and CH_2_-6, combined with COSY correlations of C-2(–C-6)–C-3–C-4–C-5, established **1** as a pyrrolidine alkaloid featuring a pyrrolidin-2-ylmethanol substituent like scalusamide A (**12**) [11]. This assignment was confirmed by HMBC correlations of H-3 with C-6, H-4 with C-2 and C-3, and H-5 with C-2 and C-3 (Figure 2). The COSY spectrum established a continuous spin system, CH-2′/CH-3′/CH_2_-4′/CH_2_-5′/CH-6′/CH-7′/CH_3_-8′, which was further confirmed and defined as a hepta-1,5-diene chain by the key HMBC correlations from H-2′ to C-4′, H-7′ to C-5′, and from both H-4′ and H-8′ to C-6′. The HMBC correlations of H-2′ and H-3′ with C-1′ implied that the amide carbonyl was connected to C-2′. Comparative analysis of scalusamides A–C (**12**–**14**) demonstrated that the pyrrolidin-2-ylmethanol substituent is covalently linked to the hepta-1,5-diene substituent via the carbonyl group C-2′. Therefore, the planar structure of **1** was determined as shown [11]. Notably, compound **1** is the first pyrrolidine alkaloid featuring an eight-carbon chain attached to the nitrogen atom of pyrrolidin-2-ylmethanol substituent.

The trans-configuration of the C-2′–C-3′ double bond in **1** was unambiguously confirmed by the observed vicinal coupling constant (^3^*J*_H-2′/H-3′_ = 15.0 Hz). The *E* configuration of the C-6′–C-7′ double bond was assigned based on the upfield chemical shift of C-8′ (*δ*_C_ 18.1, <20.0 ppm), by analogy to the established correlation for the C-13–C-14 bond in scalusamide A (**12**) (*δ*_C_ 17.7 for C-15) [11,21]. Based on the biosynthetic considerations, the pyrrolidin-5-ylmethanol moiety was suggested to originate from the reduction of *R*-proline [11]. Thus, the absolute configuration of C-2 in **1** was determined to be *R*.

Penicipyrrolidine P (**2**) was obtained as a white amorphous powder, the molecular formula was inferred to be C_15_H_25_NO_2_ from its HRESIMS ions at *m*/*z* [M + H]^+^ 252.1953 (calcd for 252.1964) and ^13^C NMR data (Table 1), indicating four degrees of unsaturation. The ^1^H NMR spectrum of **2** exhibited four olefinic protons (*δ*_H_ 6.50, 5.37, 5.37, 5.26) and two methyls at *δ*_H_ 1.60 and 1.21. The ^13^C NMR and HSQC of **2** showed two sets of signals for an amide carbonyl (*δ*_C_ 172.4 and 172.5), four olefinic carbon signals (*δ*_C_ 131.4, 128.4, 124.8, 112.9 and 131.4, 128.8, 124.7, 111.7), one oxymethine (*δ*_C_ 74.0 and 74.1), one methine (*δ*_C_ 42.4 and 42.7), six methylenes (*δ*_C_ 44.9, 35.3, 32.6, 29.6, 28.2, 25.5 and 45.6, 35.3, 32.5, 30.0, 28.1, 25.4), and two methyls (*δ*_C_ 18.0, 15.1 and 17.9, 14.9). The carbonyl group and two double bonds represent three, suggesting a ring in **2**. Considering the rotameric phenomenon of **2**, compound **2** was considered as a rotational isomer for the amide carbonyl linked with an asymmetric pyrrolidine moiety [11].

The ^1^H-^1^H COSY NMR data showed two isolated proton spin systems of the 2-decene-8-hydroxy-9-yl fragment, and C-2–C-3–C-4–C-5 subunits of **2** (Figure 2). HMBC correlations from H-4 and H-5 to C-2 resulted the formation of a dihydropyrrole ring. The connectivity among the two isolated spin systems and the one carbonyl group (C-1′) was established by analysis of the HMBC spectrum. HMBC correlations from H-5, H-3′ and H_3_-11′ to C-1′ led to form the planar structure of **2**. After relentless structural search it was found that the planar structure of **2** was similar to that of previously reported *N*-(2-methyl-3-oxodec-8-enoyl)-2-pyrroline (**15**) [22]. The only difference observed was the reduction of the keto carbonyl group at C-3′ in **15** to a hydroxyl group in **2**. In the ^1^H NMR spectrum of **2**, some signals are highly overlapped, rendering the absolute configuration of C-3′ challenging to ascertain through techniques such as the modified Mosher′s method (Figure S13).

Penicipyrrolidine Q (**3**) was isolated as a colorless oil, and assigned a molecular formula of C_15_H_25_NO_3_ (four degrees of unsaturation) by HRESIMS 290.1718 [M + Na]^+^ (calcd for 290.1732) and ^13^C NMR data (Table 1). The planar structure of **3** was established by analysis of ^1^H-^1^H COSY and HMBC spectra. Detailed comparison of the NMR data of **3** with those of **15** revealed the same skeleton. The most significant difference was the disappearance of the C-8′–C-9′ double bond, which was replaced by a methylene group and an oxygenated methine group in **3**, indicating that **3** is a hydration product of **15**. The presence of the hydroxyl group at C-8′ was supported by the key HMBC correlations from H-6′ and H-10′ to it (Figure 2). Thus, the planar structure of **3** was established as shown.

As with **2**, the assignment of the absolute configuration at C-3′ in **3** is challenging due to its rotameric nature. Although we attempted to determine the absolute configuration of the secondary hydroxyl group at C-8′ using the modified Mosher′s method, it was unsuccessful due to signal overlapping caused by rotational isomer.

Penicipyrrolidine R (**4**) was obtained as colorless oil and was inferred to have the molecular formula C_15_H_23_NO_3_ from its protonated ion [M + Na]^+^ at *m*/*z* 288.1563 (calcd 288.1576 for C_15_H_23_NO_3_), suggesting one more degree of unsaturation than that of **3**. Detailed interpretation of the 1D and 2D NMR spectra (Table 1) of **4** revealed structural similarities to **3**, suggesting they shared the same skeleton. The major difference was the presence of an additional double bond between C-6′ and C-7′ in **4**. The foregoing information is also supported by the HMBC correlations from H-6′ (*δ*_H_ 5.59, m) and H-7′ (*δ*_H_ 5.44, dd, *J* = 15.7, 6.5 Hz) to the oxymethine at C-8′ (*δ*_C_ 74.2) and C-5′ (*δ*_C_ 26.0) (Figure 2). Thus, the planar structure of **4** was established as shown. The coupling constant ^3^*J*_H-6′/H-7′_ = 15.7 Hz suggested the *E* configuration in **4**. Following a C_18_ HPLC separation, **4** was separated into **4a** and **4b**. However, **4a** and **4b** were readily epimerized at C-2′ to each other within 48 h, a phenomenon previously observed in **12** [11]. The absolute configuration at C-8′ of **4** could not be determined due to rotational isomer, as well as the epimerization phenomenon [11].

The molecular formula of penicipyrrolidine S (**5**) was established as C_15_H_25_NO_3_, indicating an identical molecular formula to that of **3**. A detailed analysis of the NMR spectra (Table 2) suggested that **5** is a pyrrolidine alkaloid with the same skeleton as **15** [22]. Apart from the same fragment of *N*-(2-methyl-3-oxodec-8-enoyl)pyrrolidine, the most obvious difference was the signals for the C-2–C-3 double bond in **15** were absent, replaced by those for a methylene group and an oxygenated methine group (C-2). The ^1^H-^1^H COSY correlations of C-2–C-3–C-4–C-5 and HMBC correlations from H-5 and H-4 to C-2 (*δ*_C_ 89.7) supported the presence of a hydroxyl group at C-2 in **5** (Figure 2). Therefore, all of the atom connections in **5** were established as shown. In contrast to **2**–**4**, rotational isomerism was not observed for **5**, which can be attributed to the steric hindrance imposed by a bulky substituent at C-2. Attempts to assign the absolute configuration of **5** using the modified Mosher′s method were hampered by epimerization at C-2′ during the prolonged standing.

Penicipyrrolidine T (**6**) had the molecular formula C_16_H_27_NO_4_ indicated by HRESIMS, representing an addition of a CH_2_O unit relative to **5**. The similarity between the ^1^H and ^13^C NMR spectra (Table 2) of **6** and those of **5**, especially the presence of *N*-(acetyl)pyrrolidine and 2-hepten-7-yl moiety. The ^1^H and ^13^C NMR spectra of **6** showed the presence of a methoxy group (*δ*_C/H_ 57.1/3.41). The HMBC correlations from H-2 (*δ*_H_ 5.29) to C-3 (*δ*_C_ 73.2), C-4 (*δ*_C_ 31.1), and C-5 (*δ*_C_ 44.6) and from the methoxy group to C-2 confirmed the planar structure of **6** and the presence of the methoxy group in C-2 and the hydroxyl group in C-3 (Figure 2). We expect to establish the relative configuration of **6** by *J*-based configuration analysis, ROESY [23]. Although the ^2^*J*_C-3,H-2_ and ^2^*J*_C-2,H-3_ coupling constants were too small for detection in the HECADE spectra, their minimal values are consistent with, and indeed support, the assigned 2S*,3S* relative configuration of compound **6**. Compound **6** also exhibits dual NMR signals due to C-2′ epimerization. However, all attempts to unambiguously determine the configuration of **6** were inconclusive. Furthermore, the limited quantity of isolated compound **6**, which was subsequently utilized for biological assays, precluded further analysis. Therefore, the absolute configuration of **6** remains to be elucidated.

Penicipyrrolidine U (**7**) was isolated as a colorless oil. The molecular formula C_13_H_23_NO_2_ (three degrees of unsaturation) was established by HRESIMS ([M + H]^+^, calcd for 226.1807) and ^13^C NMR data (Table 2). A detailed analysis of the NMR spectra (Table 2) suggested that **7** possesses the same 1-azaneyl-2-methyldec-8-ene-1,3-dione side chain as **15** [22]. The ^1^H-^1^H COSY cross peaks identified an ethyl group (C-3–C-2) and HMBC correlations from H-2 to C-1′ established the gross structure as *N*-ethyl-2-methyl-3-oxodec-8-enamide (Figure 2).

**Figure 3:**
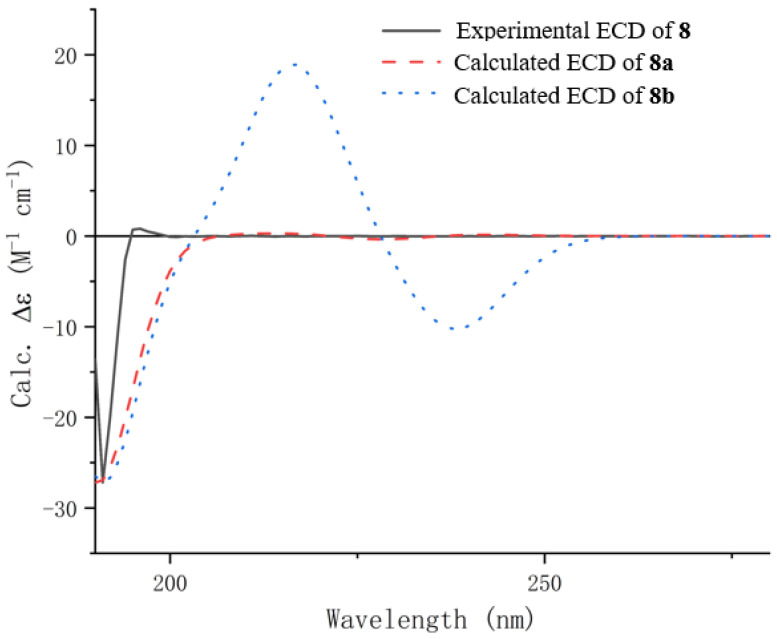
Experimental and calculated ECD spectra of compound **8**.

HRESIMS established the molecular formula of penicipyrrolidine V (**8**), a yellow oil, as C_15_H_23_NO_3_, which contains one more oxygen atom than that of (–)-penicilactam A (**11**) [24]. In the ^1^H and ^13^C NMR spectra of **8**, two methyl groups (*δ*_C/H_ 10.2/1.79; 18.1/1.64), two methines (*δ*_C/H_ 75.5/4.49) and (*δ*_C/H_ 92.4/5.02), two olefinic methines (*δ*_C/H_ 131.0/5.40) and (*δ*_C/H_ 125.4/5.39) and three quaternary carbons comprising one carbonyl carbon (*δ*_C_ 163.4) and two olefinic carbons (*δ*_C_ 163.4 and 106.7). Detailed NMR comparison between **8** and **11** established that the sole difference was the conversion of the C-3 methylene group into an oxygenated methine. Key HMBC correlations from H-4 to C-2 and C-3, and from H-5 to C-3 and C-4, along with COSY correlations between H-2, H-3, and H-4, confirmed the proposed change (Figure 2). Further analysis of ^1^H-^1^H COSY and HMBC spectra confirmed that the remainder of the structure is identical to that of **11** (Figure 2). In order to establish the absolute configuration of C-2 and C-3, TD-DFT calculations were performed on two possible enantiomeric structures of 2*S*, 3*R* (**8a**) and 2*S*, 3*S* (**8b**). The calculated ECD spectrum of **8a** was in good agreement with the experimental one for **8** (Figure 3). Thus, the absolute configuration was established as depicted.

HRESIMS established the molecular formulas of penicipyrrolidines W and X (**9** and **10**) as C_15_H_23_NO_2_ (*m*/*z* 250.1803 [M + H]^+^, calcd for 250.1807) and C_15_H_25_NO_2_ (*m*/*z* 252.1960 [M + H]^+^, calcd for 252.1964), respectively. In contrast to **9**, which shares an identical formula with **11**, compound **10** contains two more hydrogen atoms than **11**, identifying it as a dihydrogenated analogue. The ^13^C NMR data of **9** (Table 3) showed close similarity to those of **11**, with the exception of the chemical shifts of three carbons attributable to two olefinic units and one terminal methyl group. Comprehensive analyses of the ^1^H-^1^H COSY and HMBC spectra established the connectivity from the terminal methyl at C-10′ to the olefinic methine (C-7′) via two methylene units (C-8′ and C-9′), indicating that **9** possessed the double bond at C-6′–C-7′. The *E*-geometry of this double bond was assigned based on the coupling constant ^3^*J*_H-6′/H-7′_ = 15.5 Hz. Analysis of the ^1^H and ^13^C NMR data (Table 3) indicated that the data for **10** differed from those of **11** primarily by the absence of signals corresponding to olefinic functionalities, suggesting that **10** features a saturated carbon chain. The absolute configuration of C-2 was established as *S* by comparing its calculated ECD curve (2*S*) with the experimental ECD curve of **9** and **10** (Figures S58 and S65).

Five known compounds were identified as (–)-penicilactam A (**11**), scalusamides A–C (**12**–**14**) and *N*-(2-methyl-3-oxodec-8-enoyl)-2-pyrroline (**15**), by comparing their physicochemical and spectroscopic data with the reference data [11,24,25,26].

### 2.2. Glucose Uptake in L6 Skeletal Muscle Cells

The cytotoxicity of compounds **1**–**10** against L6 cells was assessed using the CCK-8 assay. Compounds **1**–**9** showed no significant effect on the viability of L6 cells at concentrations up to 100 μM, whereas only compound **10** exhibited mild cytotoxicity at this concentration (Figure S77). Therefore, a safe working concentration of 10 μM was selected for subsequent experiments.

The stimulatory effects of the isolated compounds on glucose uptake were evaluated in L6 skeletal muscle cells using the 2-NBDG assay. At a concentration of 10 μM, the compounds displayed a range of activities. Notably, compound **2** emerged as the most potent, enhancing glucose uptake by 3.83-fold relative to the untreated control. This activity was comparable to that of the positive control, metformin. Compounds **3** and **4** also showed significant, though lesser, effects, increasing uptake by 1.84-fold and 1.25-fold, respectively. These results clearly indicate that compound **2** is a promising candidate for enhancing glucose metabolism.

## 3. Materials and Methods

### 3.1. General Experimental Procedures

Optical rotations were determined using a PerkinElmer Model 341 polarimeter (PerkinElmer Inc., Waltham, MA, USA), UV-Vis absorption spectra were obtained with a Shimadzu UV-1700 spectrophotometer (Shimadzu Corporation, Kyoto, Japan), and IR spectra were collected on a Thermo Nexus 470 FT-IR spectrometer (Thermo Electron Corporation, Waltham, MA, USA). NMR spectra were acquired using a Biospin AV 400 NMR instrument (Bruker BioSpin GmbH, Rheinstetten, Germany) at 400 MHz for ^1^H NMR and 100 MHz for ^13^C NMR. High-resolution mass spectrometry data were obtained on an LTQ Orbitrap XL mass spectrometer (Thermo Fisher Scientific, Waltham, MA, USA). Column chromatography was performed using silica gel (200–300 mesh, Anhui Liangchen Guiyuan Material Ltd., Liuan, China), and size-exclusion chromatography was conducted with Sephadex LH-20 (Cytiva, Uppsala, Sweden).

### 3.2. Fungal Material

The fungus *Penicillium* sp. DM27, derived from mangrove roots, was isolated from the rhizosphere soil of *Bruguiera gymnorrhiza* (L.) Poir. on 26 September 2013 in the Tachalab subdistrict of the Tamai district in Chantaburi Province, Thailand. Tachalab is located at 102° E 3.4′ longitude, and 12° N 32′ latitude. The fungus was identified according to its morphological characteristics and the sequences of the internal transcribed spacer (ITS). A voucher specimen (*Penicillium* sp. DM27) is available for inspection at the School of Pharmaceutical Sciences, Wuhan University.

### 3.3. Mass Culture

*Penicillium* sp. DM27 was initially inoculated into potato dextrose agar medium, which consisted of 6 g of potato extract, 20 g of glucose, 15 g of sea salt, 20 g of agar, and 1 L of distilled water. The pH of the solution was subsequently adjusted to 6.5, and it was incubated for seven days at 28 °C. Following this incubation period, the culture was transferred to a 300 mL potato dextrose liquid medium contained in a 1 L flask and fermented for 28 days at 28 °C. A total of 60 flasks of the liquid medium were prepared for scale-up in this study.

### 3.4. Extraction and Isolation

The filtered brown culture yielded mycelia and a liquid phase. The mycelia were ultrasonically extracted with 100% acetone (3 × 3 L), and the combined extracts were concentrated in vacuo to give a crude extract. This extract was then combined with the liquid phase (30 L) and fractionated over macroreticular resin HP20, eluting with MeOH/H_2_O (30%, 50%, 70%, 90%) to yield four fractions, designated as A, B, C, and D. Fraction A (5.24 g) was fractionated by silica gel column chromatography (CC) with a stepwise gradient of CH_2_Cl_2_/MeOH (40:1 to 1:1, *v*/*v*) to yield eighteen subfractions (Fr.A.1–Fr.A.18). Fr.A.3 (26 mg) was further purified by RP-HPLC on a Sepex-C18 column (250 × 10 mm, 5 μm) using an isocratic eluent of MeOH/H_2_O (60:90, *v*/*v*) over 30 min at a flow rate of 3.0 mL/min, affording compound **1** (1 mg, *t*_R_ = 20 min). Fr.A.3 (108 mg) was chromatographed over Sephadex LH-20 (eluent: MeOH/CH_2_Cl_2_, 1:1) to give five subfractions (Fr.A.5.1–Fr.A.5.5). Subsequent purification of Fr.A.5.3 (23 mg) by RP-HPLC with MeOH/H_2_O (35:50, *v*/*v*; 45 min) yielded compound **4** (8 mg, *t*_R_ = 20 min) and **7** (1 mg, *t*_R_ = 25 min). Fr.A.18 (98 mg) was separated Sephadex LH-20 washed with MeOH-CH_2_Cl_2_ = 1:1 to provide five fractions (Fr.A.18.1 to Fr.A.18.5) then Fr.A.18.5 (15 mg) separated by RP-HPLC with MeOH-H_2_O (55:45 *v*/*v*) to yield **10** (1 mg, *t*_R_ = 20 min). The Fr.B (3.20 g) was separated into ten fractions (Fr.B.1 to Fr.B.10) by silica gel CC with elution with mixtures of PE/EA from 10:1 to 0:1 (*v*/*v*). Fr.B.9 (23 mg) was further separated into four fractions (Fr.B.9.1 to Fr.B.9.4) with CH_2_Cl_2_/MeOH 30:1 to 0:1. Fr.B.9.2 was separated by RP-HPLC with MeOH:H_2_O (50:50 *v*/*v*, 30 min) to yield **3** (2 mg, *t*_R_ = 20.4 min). Fr.B.9.3 (25 mg) was further purified by RP-HPLC with MeOH:H_2_O (50:30 to 70:20, 20 min) to afford **2** (5 mg, *t*_R_ = 15 min). Fr.C (4.50 g) was subjected to silica gel columns with CH_2_Cl_2_/MeOH (100:1 to 0:1, *v*/*v*) to nineteen fractions (Fr.C.1 to Fr.C.19). Fr.C.4 (340 mg) was separated by Sephadex LH-20 eluting with 1:1 CH_2_Cl_2_–MeOH into four fractions. (Fr.C.4.1 to Fr.C.4.4) and the resulting subfractions Fr.C.4.1 (35 mg) were purified by RP-HPLC to afford **6** (3 mg, *t*_R_ = 20 min, 30% MeCN) and **8** (1 mg, *t*_R_ = 20 min, 37% MeCN). Fr.C.6 (500 mg) was subjected to Sephadex LH-20 (MeOH-CH_2_Cl_2_/1:1) and the resulting subfractions were purified by RP-HPLC with 30% MeCN in H_2_O for 30 min to afford **9** (1 mg, *t*_R_ = 17min). Fr.C.7 (100 mg) was separated Sephadex LH-20 washed with MeOH-CH_2_Cl_2_/1:1 to provide eight fractions (Fr.C.7.1 to Fr.C.7.8). Fr.C.7.3 (30 mg) was purified by RP-HPLC with MeOH:H_2_O (50% to 100%, *v*/*v*, 20 min) to afford **5** (1 mg, *t*_R_ = 11.0 min).

*Penicipyrrolidine O* (**1**): Colorless oil; [α${]}_{D}^{20}$ −22.0 (*c* 0.10, MeOH); ^1^H and ^13^C NMR data (CDCl_3_), see Table 1; HRESIMS *m*/*z* 246.1463 [M + Na]^+^ (calcd for C_13_H_21_NO_2_, 246.1470).

*Penicipyrrolidine P* (**2**): White amorphous powder; [α${]}_{D}^{20}$ −15.0 (*c* 0.08, MeOH); UV (MeOH) *λ*_max_ (log *ε*) 206 (3.87), 286 (3.03) nm; ECD {MeOH, *λ* [nm] (Δ*ε*), *c* = 0.1 × 10^−4^ M} 216 (−7.00), 202.4 (−7.86), 196.4 (−7.50), 189.8 (−17.73); ^1^H and ^13^C NMR data (CDCl_3_), see Table 1; HRESIMS *m*/*z* 252.1953 [M + H]^+^ (calcd for C_15_H_25_NO_2_, 252.1964).

*Penicipyrrolidine Q* (**3**): Colorless oil; [α${]}_{D}^{20}$ +18.2 (*c* 0.02, MeOH); UV (MeOH) *λ*_max_ (log *ε*) 287 (2.01), 224 (2.42), 203 (3.10) nm; ECD {MeOH, *λ* [nm] (Δ*ε*), *c* = 0.1 × 10^−4^ M} 241 (−13.00), 206.9 (−7.25), 191.8 (−8.98); IR (KBr) *ν*_max_ 3446, 2926, 1624, 1515, 1384, 1318, 1122, 1053 cm^−1^; ^1^H and ^13^C NMR data (CDCl_3_), see Table 1; HRESIMS *m*/*z* 290.1718 [M + Na]^+^ (calcd for C_15_H_25_NO_3_, 290.1732).

*Penicipyrrolidine R* (**4**): Colorless oil; [α${]}_{D}^{20}$ −24.6 (*c* 0.11, MeOH); UV (MeOH) *λ*_max_ (log *ε*) 203 (4.42), 273 (3.23) nm; ECD {MeOH, *λ* [nm] (Δ*ε*), *c* = 0.1 × 10^−4^ M} 211.6 (+9.47), 198.4 (−17.30), 189 (+42.80); ^1^H and ^13^C NMR data (CDCl_3_), see Table 1; HRESIMS *m*/*z* 288.1563 [M + Na]^+^ (calcd for C_15_H_23_NO_3_, 288.1576).

*Penicipyrrolidine S* (**5**): Colorless oil; [α${]}_{D}^{20}$ −6.6 (*c* 0.02, MeOH); ^1^H and ^13^C NMR data (CDCl_3_), see Table 2; HRESIMS *m*/*z* 290.1718 [M + Na]^+^ (calcd for C_15_H_25_NO_3_, 290.1732).

*Penicipyrrolidine T* (**6**): Colorless oil; [α${]}_{D}^{20}$ −15.3 (*c* 0.10, MeOH); ^1^H and ^13^C NMR data (CDCl_3_), see Table 2; HRESIMS *m*/*z* 320.1823 [M + Na]^+^ (calcd for C_16_H_27_NO_4_, 320.1837).

*Penicipyrrolidine U* (**7**): Colorless oil; [α${]}_{D}^{20}$ −18.7 (*c* 0.10, MeOH); ^1^H and ^13^C NMR data (CDCl_3_), see Table 2; HRESIMS *m*/*z* 226.1800 [M + H]^+^ (calcd for C_13_H_23_NO_2_, 226.1807).

*Penicipyrrolidine V* (**8**): Yellow oil; [α${]}_{D}^{20}$ −9.3 (*c* 0.10, MeOH); ^1^H and ^13^C NMR data (CDCl_3_), see Table 3; HRESIMS *m*/*z* 288.1571 [M + Na]^+^ (calcd for C_15_H_23_NO_3_, 288.1576).

*Penicipyrrolidine W* (**9**): Colorless oil; [α${]}_{D}^{20}$ −2.6 (*c* 0.10, MeOH); ^1^H and ^13^C NMR data (CDCl_3_), see Table 3; HRESIMS *m*/*z* 250.1803 [M + H]^+^ (calcd for C_15_H_23_NO_2_, 250.1807).

*Penicipyrrolidine X* (**10**): Colorless oil; [α${]}_{D}^{20}$ −2.0 (*c* 0.10, MeOH); ^1^H and ^13^C NMR data (CDCl_3_), see Table 3; HRESIMS *m*/*z* 252.1960 [M + H]^+^ (calcd for C_15_H_25_NO_2_, 252.1964).

### 3.5. Computational Analysis

To achieve conformational optimization of the stereoisomers, computational studies were conducted using density functional theory (DFT). Initial conformational sampling was performed with the MMFF94 molecular mechanics force field. The resulting low-energy conformers were subsequently optimized at the DFT level using the Gaussian 09 software package with the 6-311G (2d, p) basis set for all atoms [27]. Room-temperature equilibrium populations were estimated based on the Boltzmann distribution law (Equation (1)). The ECD spectra for individual conformers were simulated using a Gaussian function, and the final composite spectra were generated by Boltzmann weighted averaging of the respective conformer spectra.

(1) $$\frac{N_{i}}{N}=\frac{g_{i}e^{-\frac{E_{i}}{k_{B}T}}}{\sum g_{i}e^{-\frac{E_{i}}{k_{B}T}}}$$

where *N_i_* is the number of conformer *i* with energy *E_i_* and degeneracy *g_i_* at temperature *T*, and *k*_B_ is Boltzmann constant.

### 3.6. Cell Culture and Treatment

Rat skeletal muscle L6 cells were obtained from Wuhan Procell Life Science & Technology Co., Ltd., Wuhan, China and were cultured in complete medium consisting of 89% α-MEM, 10% FBS, and 1% antibiotics. After passaging and stable growth, the cells were cultured in α-MEM medium supplemented with 2% FBS. The medium was changed daily for 7 days to induce differentiation into myotubes.

The potential cytotoxicity of the test compounds was evaluated using a Cell Counting Kit-8 (CCK-8) assay. Briefly, L6 myotubes were seeded in 96-well plates at a density of 1 × 10^4^ to 5 × 10^4^ cells per well. After differentiation, the cells were treated with the test compounds at four different concentrations (3, 10, 30, and 100 μM), prepared in a medium containing a final concentration of 0.1% DMSO. A vehicle control group (0.1% DMSO in medium) and a blank control (medium only) were included. After 24 h of incubation, the medium was replaced with 100 μL of fresh α-MEM containing 10% (*v*/*v*) CCK-8 reagent. The plates were incubated for an additional 2 h at 37 °C. The absorbance was measured at 450 nm using a microplate reader. Cell viability was expressed as a percentage relative to the vehicle control group.

Myotubes were seeded in 96-well plates at a density of 1 × 10^4^ to 5 × 10^4^ cells per well, with 100 μL of α-MEM medium added to each well until the cells covered the dish. Three groups were established: a blank control, a metformin positive control, and a drug administration group (10 μM), each with three sub-wells. 100 μL of α-MEM medium containing 2-NBDG and each compound (10 μM) was added to the myotubes and incubated for 30 min in a constant temperature incubator. After incubation, the 96-well plates were centrifuged for 5 min at 400× *g*, the supernatant was discarded, and 200 L of kit buffer was added to each cell well, mixed, and centrifuged again at 400× *g* for 5 min at room temperature. This process was repeated. The glucose uptake capacity of the cells was assessed by measuring the 2-NBDG fluorescence intensity.

## 4. Conclusions

The isolation of penicipyrrolidines O–X (**1**–**10**) from the mangrove-derived fungus *Penicillium* sp. DM27 expands the structural diversity of pyrrolidine derivatives characterized by eight- or eleven-carbon atom side chains. In compounds **2**–**4** and **15**, the two sets of signals are ascribed to rotamers arising from restricted amide bond rotation, which exist in a dynamic equilibrium, as supported by the observation of *trans* and *cis* amide conformers in a ratio of approximately 3:1. The rotational isomers are absent in compounds **1**, **5**–**6**, and **12**–**14** due to the bulky substituent at the 2-position. The two sets of signals observed in compounds **6** and **12**–**14** originates from epimerization at the 2′-position, a configurational inversion process at a chiral center. Immediate analysis of the freshly isolated pure compound—as exemplified by **12** (Figures S69 and S70)—reveals a single set of NMR signals. However, upon prolonged standing, epimerization proceeds, leading to the reappearance of two sets of signals, as illustrated by **13** and **14** (Figures S71–S74). Consequently, the presence of rotational isomerism alongside time-dependent epimerization at C-2′ precluded the reliable determination of the absolute configurations for those compounds using methods such as the modified Mosher′s method. Upon evaluation of glucose uptake activity in L6 cells for all compounds, compound **2** demonstrated the highest activity at 3.83-fold, comparable to metformin, while compounds **3** and **4** were less active at 1.84-fold and 1.25-fold, respectively.

## Figures and Tables

**Table 1:** ^1^H and ^13^C NMR spectroscopic data for compounds **1**–**4** in CDCl_3_ (*δ* in ppm, *J* in Hz).

No.	1 *^a^*		2 *^b^*		3 *^b^*		4 *^b^*	
	*δ*_C_	*δ*_H_	*δ*_C_	*δ*_H_	*δ*_C_	*δ*_H_	*δ*_C_	*δ*_H_
2	61.6	4.28, m	128.4	6.50, m	128.7	6.54, m	128.4	6.56, d (4.5)
3	28.5	2.05, m; 1.59, m	112.9	5.26, m	113.7	5.29, m	113.5	5.29, m
4	24.6	1.96, dt (13.2, 6.5) 1.88, dt (12.5, 6.5)	28.2	2.60, m	28.6	2.66, m	28.3	2.78, t (9.1)
5	48.2	3.63, m; 3.55, m	44.9	3.80, m	46.0	3.86, t (9.3)	45.6	3.87, t (9.0)
6	67.9	3.68, m; 3.61, m						
1′	167.6		172.5		165.7		165.7	
2′	121.6	6.12, dt (15.0,1.7)	42.4	2.56, m	53.1	3.58, q (7.0)	53.2	3.58, q (7.0)
3′	147.1	6.96, dt (15.0, 7.0)	74.0	3.59, m	207.5		206.1	
4′	32.7	2.28, q (7.0)	35.3	1.45, m	39.7	2.54, m; 2.47, m	39.1	2.60, m; 2.53, m
5′	31.4	2.15, dt (8.7, 7.0)	25.5	1.45, m; 1.32, m	23.8	1.60, m	26.1	2.30, m
6′	129.9	5.42, m	29.6	1.34, m	25.5	1.42, m; 1.32, m	130.0	5.59, m
7′	126.2	5.46, m	32.6	1.94, m	37.0	1.43, m	134.1	5.44, dd (15.7, 6.5)
8′	18.1	1.65, d (4.9)	131.4	5.37, m	73.4	3.48, m	74.3	3.93, m
9′			124.8	5.37, m	30.6	1.45, m	30.3	1.51, m
10′			18.0	1.60, d (3.9)	10.4	0.92, t (7.4)	9.9	0.87, t (7.4)
11′			15.1	1.21, d (7.1)	13.6	1.37, d (7.0)	13.2	1.37, d (7.1)

*^a^* Spectra were recorded at 400 MHz for ^1^H NMR and at 151 MHz for ^13^C NMR. *^b^* Spectra were recorded at 400 MHz for ^1^H NMR and at 101 MHz for ^13^C NMR.

**Table 2:** ^1^H and ^13^C NMR spectroscopic data for compounds **5**–**7** in CDCl_3_ (*δ* in ppm, *J* in Hz).

No.	5 *^a^*		6 *^b,c^*				7 *^b^*	
	δ_C_	δ_H_	δ_C_	δ_H_	δ_C_	δ_H_	δ_C_	δ_H_
1-NH								6.23, s
2	89.7	5.62, m	92.6	5.29, s	94.3	4.70, s	34.7	3.28, q (7.3)
3	29.8	1.97, m	73.2	4.20, m	73.3	4.37, m	14.8	1.12, d (7.3)
4	21.3	2.03, m; 1.91, m	31.1	2.27, m	30.0	2.19, m		
5	45.8	3.56, m; 3.51, m	44.6	3.57, m	44.2	3.64, m		
1′	170.1		171.4		172.2		169.5	
2′	52.7	4.08, q (7.2)	53.0	3.49, m	53.0	3.57, m	54.4	3.41, q (7.2)
3′	208.6		206.8		207.5		210.6	
4′	39.3	2.49, td (7.4, 1.1)	39.2	2.41, m	39.8	2.53, m	41.7	2.54, td (7.3, 3.7)
5′	23.1	1.53, p (7.4)	23.1	1.53, m	23.1	1.53, m	22.9	1.55, p (7.4)
6′	29.1	1.34, m	29.1	1.29, m	29.1	1.29, m	29.0	1.32, m
7′	32.5	1.98, m	32.4	1.94, m	32.4	1.94, m	32.4	1.97, m
8′	131.0	5.38, m	131.2	5.38, m	131.2	5.38, m	130.9	5.39, m
9′	125.3	5.40, m	125.3	5.39, m	125.3	5.39, m	125.2	5.39, m
10′	18.0	1.63, d (4.7)	18.1	1.62, d (3.6)	18.1	1.62, d (3.6)	18.0	1.63, d (4.9)
11′	14.0	1.41, d (7.1)	13.8	1.36, d (7.0)	14.2	1.41, d (7.1)	15.5	1.37, d (7.2)
2-OCH3			57.1	3.41, s	55.2	3.31, s		

*^a^* Spectra were recorded at 400 MHz for ^1^H NMR and at 101 MHz for ^13^C NMR. *^b^* Spectra were recorded at 400 MHz for ^1^H NMR and at 151 MHz for ^13^C NMR. *^c^* Spectra exhibited two sets of signals due to C-2′ epimerization of **6**.

**Table 3:** ^1^H and ^13^C NMR spectroscopic data for compounds **8**–**10** in CDCl_3_ (*δ* in ppm, *J* in Hz).

No.	8 *^a^*		9 *^b^*		10 *^a^*	
	δ_C_	δ_H_	δ_C_	δ_H_	δ_C_	δ_H_
2	92.4	5.02, d (3.8)	87.9	5.20, dd (6.0, 4.5)	87.7	5.20, dd (6.0, 4.6)
3	75.5	4.49, m	31.9	2.29, m; 2.21, m	31.9	2.28, m; 2.13, m
4	30.1	2.28, m; 1.93, m	22.1	2.01, m; 1.85, m	22.0	2.00, m; 1.86, m
5	41.8	3.65, m	44.6	3.73, m; 3.42, m	44.4	3.72, ddd (11.2, 7.5, 6.2); 3.42, ddd (11.2, 7.6, 5.9)
1′	163.4		163.7		164.0	
2′	106.7		107.0		106.5	
3′	163.4		163.2		163.7	
4′	30.6	2.30, dt (15.0, 7.8); 2.24, m	31.1	2.36, m; 2.22, m	30.7	2.28, m; 2.17, m
5′	26.4	1.53, p (7.6)	30.1	2.21, m	27.0	1.50, m
6′	29.3	1.38, m	128.8	5.39, m	29.4	1.29, m
7′	32.4	1.99, q (6.4)	131.9	5.45, dt (15.1, 6.5)	29.2	1.29, m
8′	131.0	5.40, m	34.8	1.95, q (7.3)	31.9	1.29, m
9′	125.4	5.39, m	22.8	1.35, m	22.8	1.32, m; 1.26, m
10′	18.1	1.64, d (4.7)	13.9	0.88, t (7.4)	14.2	0.87, t (6.9)
11′	10.2	1.79, s	10.3	1.79, s	10.2	1.79, s

*^a^* Spectra were recorded at 400 MHz for ^1^H NMR and at 151 MHz for ^13^C NMR. *^b^* Spectra were recorded at 600 MHz for ^1^H NMR and at 151 MHz for ^13^C NMR.

## Data Availability

The original data presented in the study are included in the article and Supplementary Material; further inquiries can be directed to the corresponding author.

## Supplementary Materials

The following supporting information can be downloaded at https://www.mdpi.com/article/10.3390/md23120455/s1, Figures S1–S12, S14–S50, S52–S57 and S59–S64: 1D and 2D NMR spectra, HRESIMS spectra of **1–10**; Figures S13: ^1^H NMR spectrum of the (*S*)-MTPA and (*R*)-MTPA esters; Figures S67–S76: 1D NMR spectra of **11–15**; Figures S51, S58 and S65–S66: Experimental ECD spectra of **8–11**; Figure S77: Cell viability of L6 cells treated with **1–10**.
