# Supplementary material for: Pyrrolidine Alkaloids from Mangrove Fungus Penicillium sp. DM27 Enhance L6 Cell Glucose Uptake
Source: Mar Drugs. 2025 Nov 27;23(12):455. doi: 10.3390/md23120455 (PMC12734938; doi:10.3390/md23120455)

Supporting Information

Pyrrolidine Alkaloids from Mangrove Fungus *Penicillium* sp. DM27 Enhance L6 Cell Glucose Uptake

Feng-Kai Fan[1,†], Wen-Ting Zhang[1,2†], Philomina Panin Edjah[2], Qing-Qing Tang[2], Wenqing Huang[3], Li-Ming He[2], Ming-Qi Zhou[2], Cong-Kui Tian[4], Kong-Kai Zhu[5], Xinzhou Yang[3], You-Sheng Cai[1,2], Kui Hong[2,*], Yuan-Zhen Liu[1,2,*]

1 Hubei Key Laboratory of Purification and Application of Plant Anticancer Active Ingredients, School of Chemistry and Life Science, Hubei University of Education, Wuhan, 430205, China
2 Key Laboratory of Combinatorial Biosynthesis and Drug Discovery, Ministry of Education and School of Pharmaceutical Sciences, Wuhan University, Wuhan, 430071, China
3 International Cooperation Base for Active Substances in Traditional Chinese Medicine in Hubei Province, School of Pharmaceutical Sciences, South-Central Minzu University, Wuhan, 430074, China
4 Hubei Key Laboratory of Selenium Resources Research and Biological Applications, Institute of selenium science and industry, Hubei Minzu University, Enshi, Hubei 445000, China
5 Advanced Medical Research Institute, Shandong University, Jinan, Shandong 250012, China
* Correspondence: kuihong31@whu.edu.cn; cysh2002@whu.edu.cn; liuyuanzhen@hue.edu.cn
† These authors contributed equally to this work.

List of Supporting Information

No.		Contents	Page
1	**Figure S1**	HR-ESIMS spectrum of **1.**	S1
2	**Figure S2**	^{1}H NMR spectrum of **1** (400 MHz, $CDCl_3$).	S1
3	**Figure S3**	^{13}C NMR spectrum of **1** (151 MHz, $CDCl_3$).	S2
4	**Figure S4**	^{1}H-^{1}H COSY spectrum of **1** (600 MHz, $CDCl_3$).	S2
5	**Figure S5**	HSQC spectrum of **1** (600 MHz, $CDCl_3$).	S3
6	**Figure S6**	HMBC spectrum of **1** (600 MHz, $CDCl_3$).	S3
7	**Figure S7**	HR-ESIMS spectrum of **2.**	S4
8	**Figure S8**	^{1}H NMR spectrum of **2** (400 MHz, $CDCl_3$).	S4
9	**Figure S9**	^{13}C NMR spectrum of **2** (101 MHz, $CDCl_3$).	S5
10	**Figure S10**	^{1}H-^{1}H COSY spectrum of **2** (400 MHz, $CDCl_3$).	S5
11	**Figure S11**	HSQC spectrum of **2** (400 MHz, $CDCl_3$).	S6
12	**Figure S12**	HMBC spectrum of **2** (400 MHz, $CDCl_3$).	S6
13	**Figure S13**	^{1}H NMR spectrum of the (*S*)-MTPA (**2a,** green) and (*R*)-MTPA (**2b,** red) esters (600 MHz, pyr-d_6).	S7
14	**Figure S14**	HR-ESIMS spectrum of **3.**	S7
15	**Figure S15**	^{1}H NMR spectrum of **3** (400 MHz, $CDCl_3$).	S8
16	**Figure S16**	^{13}C NMR spectrum of **3** (101 MHz, $CDCl_3$).	S8
17	**Figure S17**	^{1}H-^{1}H COSY spectrum of **3** (400 MHz, $CDCl_3$).	S9
18	**Figure S18**	HSQC spectrum of **3** (400 MHz, $CDCl_3$).	S9
19	**Figure S19**	HMBC spectrum of **3** (400 MHz, $CDCl_3$).	S10
20	**Figure S20**	HR-ESIMS spectrum of **4.**	S10
21	**Figure S21**	^{1}H NMR spectrum of **4** (400 MHz, $CDCl_3$).	S11
22	**Figure S22**	^{13}C NMR spectrum of **4** (101 MHz, $CDCl_3$).	S11
23	**Figure S23**	^{1}H-^{1}H COSY spectrum of **4** (400 MHz, $CDCl_3$).	S12
24	**Figure S24**	HSQC spectrum of **4** (400 MHz, $CDCl_3$).	S12
25	**Figure S25**	HMBC spectrum of **4** (400 MHz, $CDCl_3$).	S13
26	**Figure S26**	HR-ESIMS spectrum of **5.**	S13
27	**Figure S27**	^{1}H NMR spectrum of **5** (400 MHz, $CDCl_3$).	S14
28	**Figure S28**	^{13}C NMR spectrum of **5** (101 MHz, $CDCl_3$).	S14
29	**Figure S29**	^{1}H-^{1}H COSY spectrum of **5** (400 MHz, $CDCl_3$).	S15
30	**Figure S30**	HSQC spectrum of **5** (400 MHz, $CDCl_3$).	S15
31	**Figure S31**	HMBC spectrum of **5** (400 MHz, $CDCl_3$).	S16
32	**Figure S32**	NOESY spectrum of **5** (400 MHz, CDCl3).	S16
33	**Figure S33**	HR-ESIMS spectrum of **6.**	S17
34	**Figure S34**	^{1}H NMR spectrum of **6** (400 MHz, $CDCl_3$).	S17
35	**Figure S35**	^{13}C NMR spectrum of **6** (151 MHz, $CDCl_3$).	S18

36 **Figure S36** ^{1}H-^{1}H COSY spectrum of **6** (600 MHz, $CDCl_3$). S18
37 **Figure S37** HSQC spectrum of **6** (600 MHz, $CDCl_3$). S19
38 **Figure S38** HMBC spectrum of **6** (600 MHz, $CDCl_3$). S19
39 **Figure S39** HR-ESIMS spectrum of **7.** S20
40 **Figure S40** ^{1}H NMR spectrum of **7** (400 MHz, $CDCl_3$). S20
41 **Figure S41** ^{13}C NMR spectrum of **7** (151 MHz, $CDCl_3$). S21
42 **Figure S42** ^{1}H-^{1}H COSY spectrum of **7** (600 MHz, $CDCl_3$). S21
43 **Figure S43** HSQC spectrum of **7** (600 MHz, $CDCl_3$). S22
44 **Figure S44** HMBC spectrum of **7** (600 MHz, $CDCl_3$). S22
45 **Figure S45** HR-ESIMS spectrum of **8.** S23
46 **Figure S46** ^{1}H NMR spectrum of **8** (400 MHz, $CDCl_3$). S23
47 **Figure S47** ^{13}C NMR spectrum of **8** (151 MHz, $CDCl_3$). S24
48 **Figure S48** ^{1}H-^{1}H COSY spectrum of **8** (600 MHz, $CDCl_3$). S24
49 **Figure S49** HSQC spectrum of **8** (600 MHz, $CDCl_3$). S25
50 **Figure S50** HMBC spectrum of **8** (600 MHz, $CDCl_3$). S25
51 **Figure S51** ECD spectrum of **8** in MeOH. S26
52 **Figure S52** HR-ESIMS spectrum of **9.** S26
53 **Figure S53** ^{1}H NMR spectrum of **9** (600 MHz, $CDCl_3$). S27
54 **Figure S54** ^{13}C NMR spectrum of **9** (151 MHz, $CDCl_3$). S27
55 **Figure S55** ^{1}H-^{1}H COSY spectrum of **9** (600 MHz, $CDCl_3$). S28
56 **Figure S56** HSQC spectrum of **9** (600 MHz, $CDCl_3$). S28
57 **Figure S57** HMBC spectrum of **9** (600 MHz, $CDCl_3$). S29
58 **Figure S58** ECD spectrum of **9** in MeOH. S29
59 **Figure S59** HR-ESIMS spectrum of **10.** S30
60 **Figure S60** ^{1}H NMR spectrum of **10** (400 MHz, $CDCl_3$). S30
61 **Figure S61** ^{13}C NMR spectrum of **10** (151 MHz, $CDCl_3$). S31
62 **Figure S62** ^{1}H-^{1}H COSY spectrum of **10** (600 MHz, $CDCl_3$). S31
63 **Figure S63** HSQC spectrum of **10** (600 MHz, $CDCl_3$). S32
64 **Figure S64** HMBC spectrum of **10** (600 MHz, $CDCl_3$). S32
65 **Figure S65** ECD spectrum of **10** in MeOH. S33
66 **Figure S66** ECD spectrum of **11** in MeOH. S33
67 **Figure S67** ^{1}H NMR spectrum of **11** (400 MHz, $CDCl_3$). S34
68 **Figure S68** ^{13}C NMR spectrum of **11** (101 MHz, $CDCl_3$). S34
69 **Figure S69** ^{1}H NMR spectrum of **12** (400 MHz, $CDCl_3$). S35
70 **Figure S70** ^{13}C NMR spectrum of **12** (101 MHz, $CDCl_3$). S35
71 **Figure S71** ^{1}H NMR spectrum of **13** (400 MHz, $CDCl_3$). S36
72 **Figure S72** ^{13}C NMR spectrum of **13** (151 MHz, $CDCl_3$). S36

73	**Figure S73**	^{1}H NMR spectrum of **14** (400 MHz, $CDCl_3$).	S37
74	**Figure S74**	^{13}C NMR spectrum of **14** (151 MHz, $CDCl_3$).	S37
75	**Figure S75**	^{1}H NMR spectrum of **15** (400 MHz, $CDCl_3$).	S38
76	**Figure S76**	^{13}C NMR spectrum of **15** (101 MHz, $CDCl_3$).	S38
77	**Figure S77**	Cell viability of L6 cells treated with compounds **1−10** (n = 5).	S39

Figure S1 HRESIMS spectrum of **1**.

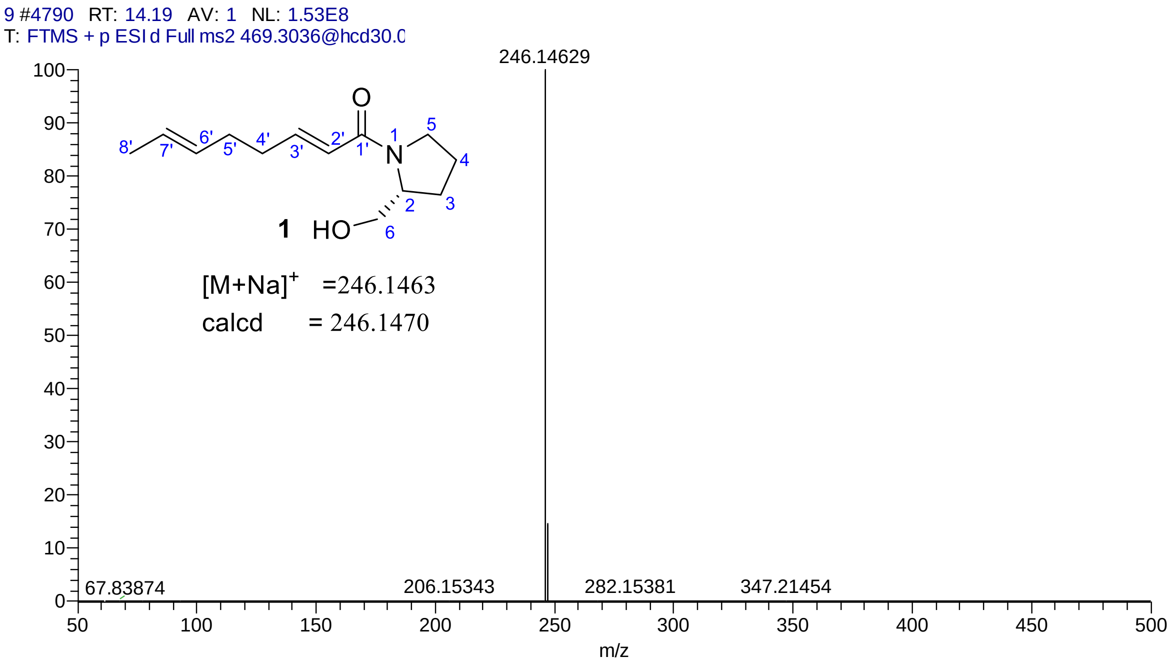

Figure S2 ^{1}H NMR spectrum of **1** (400 MHz, $CDCl_3$).

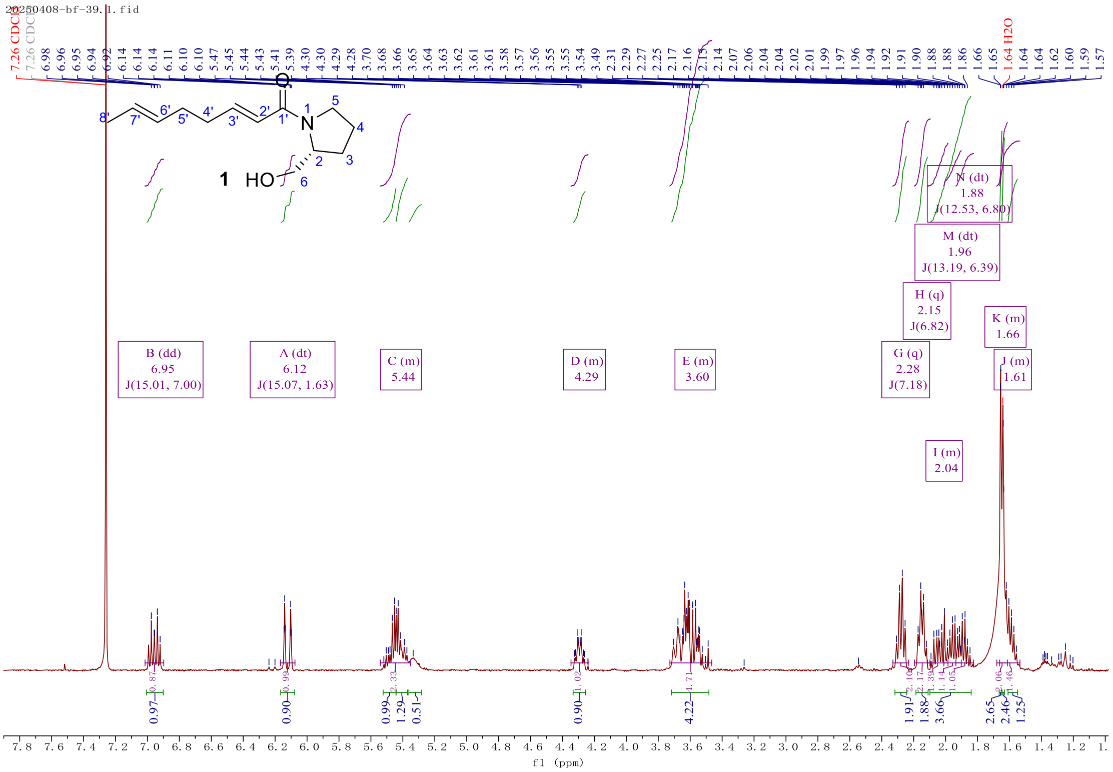

Figure S3 ^{13}C NMR spectrum of **1** (151 MHz, $CDCl_3$).

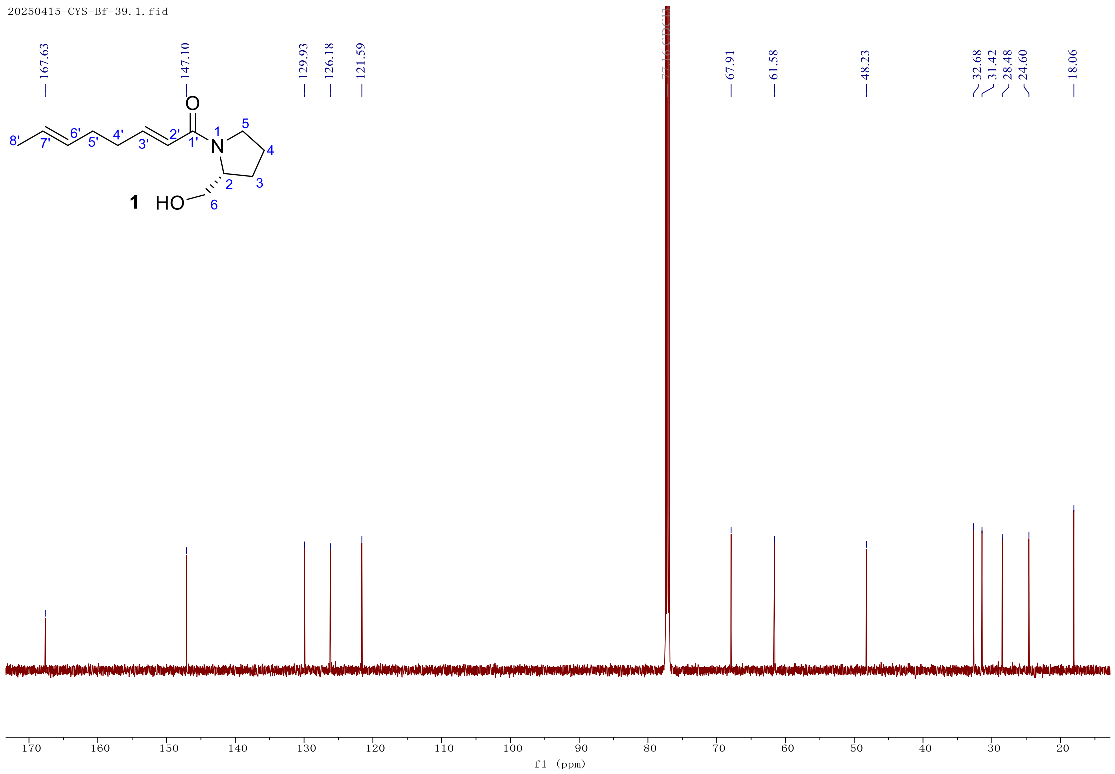

Figure S4 ^{1}H-^{1}H COSY spectrum of **6** (600 MHz, $CDCl_3$).

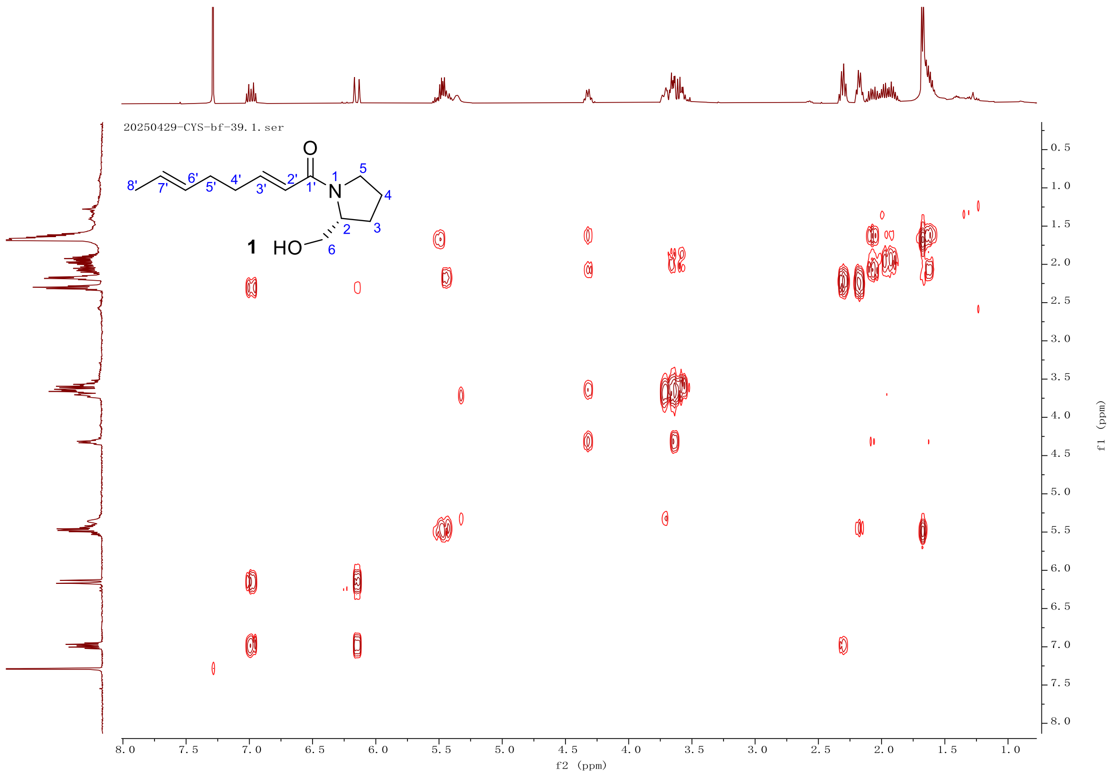

Figure S5 HSQC spectrum of **1** (600 MHz, $CDCl_3$).

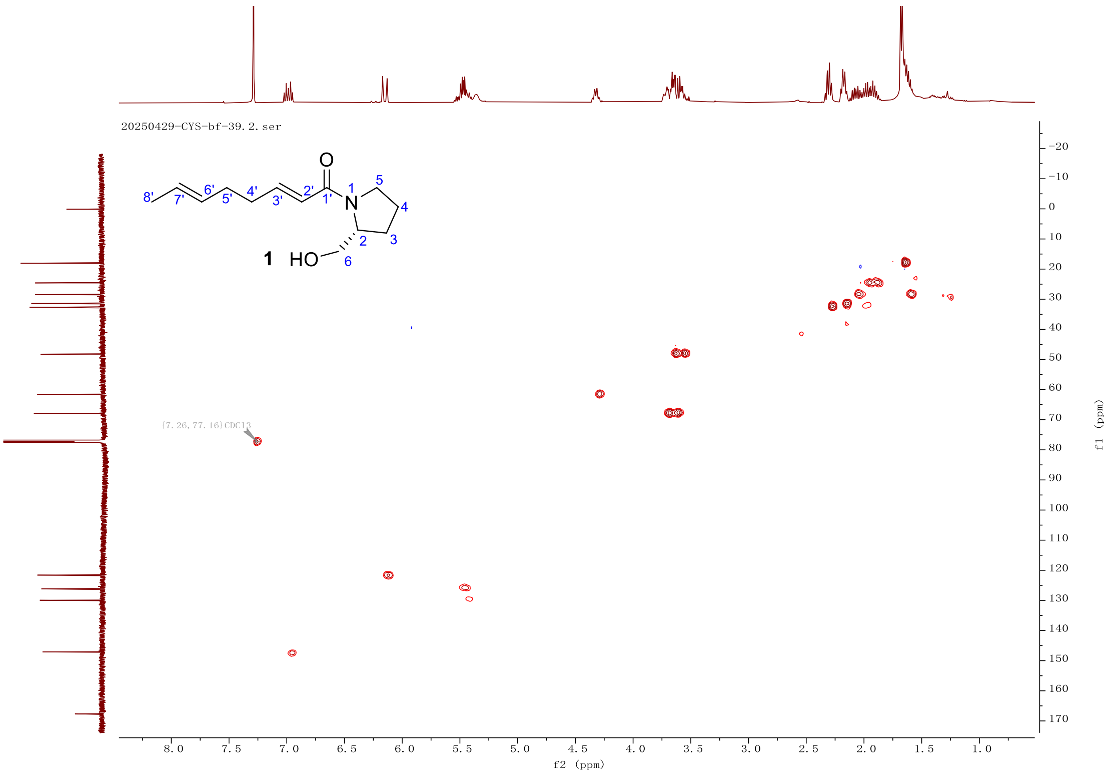

Figure S6 HMBC spectrum of **1** (600 MHz, $CDCl_3$).

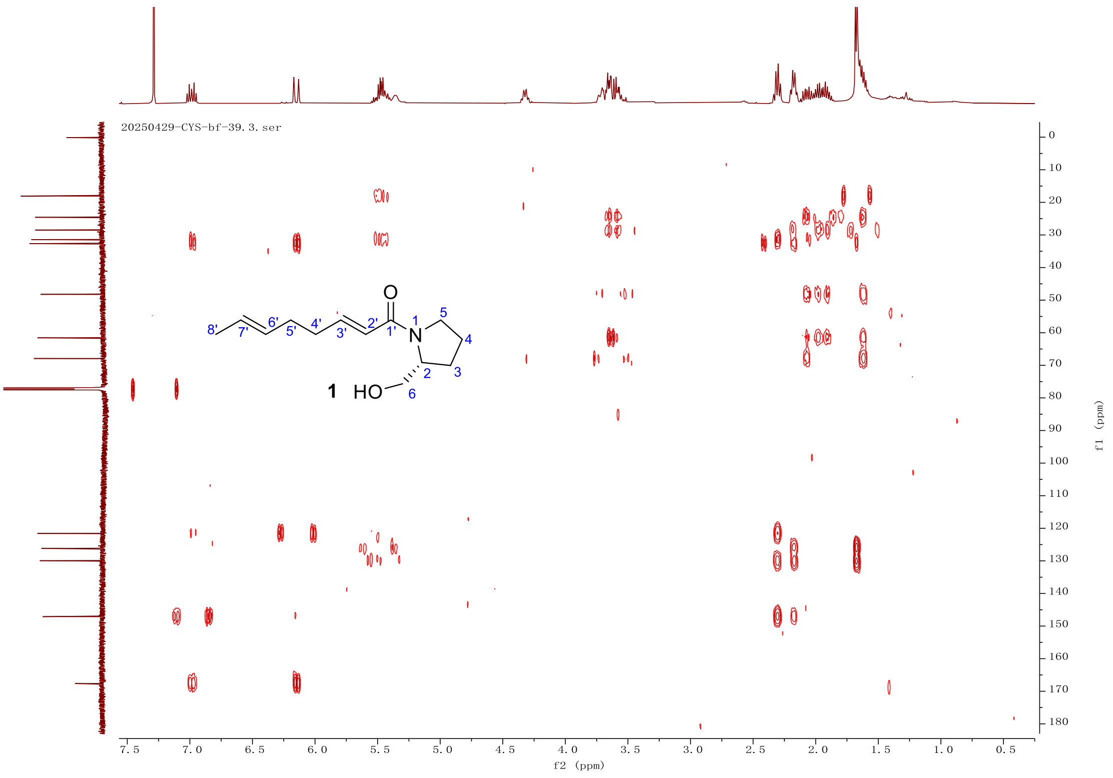

Figure S7 HRESIMS spectrum of **2**.

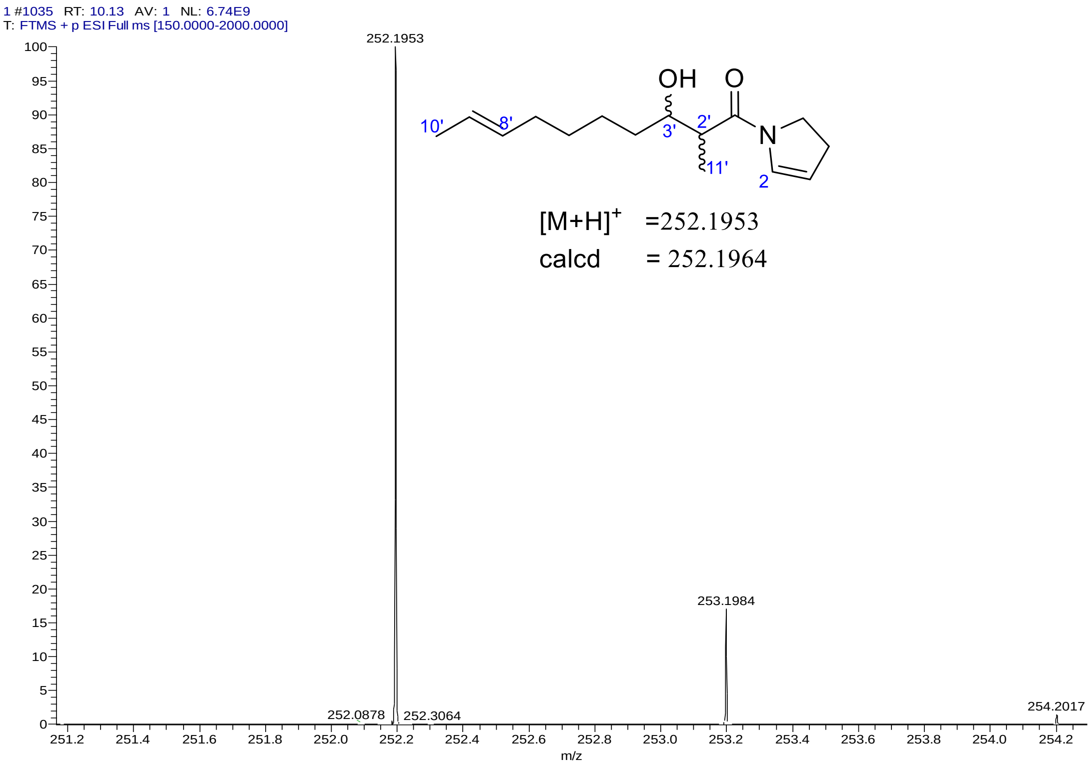

Figure S8 ^{1}H NMR spectrum of **2** (400 MHz, $CDCl_3$).

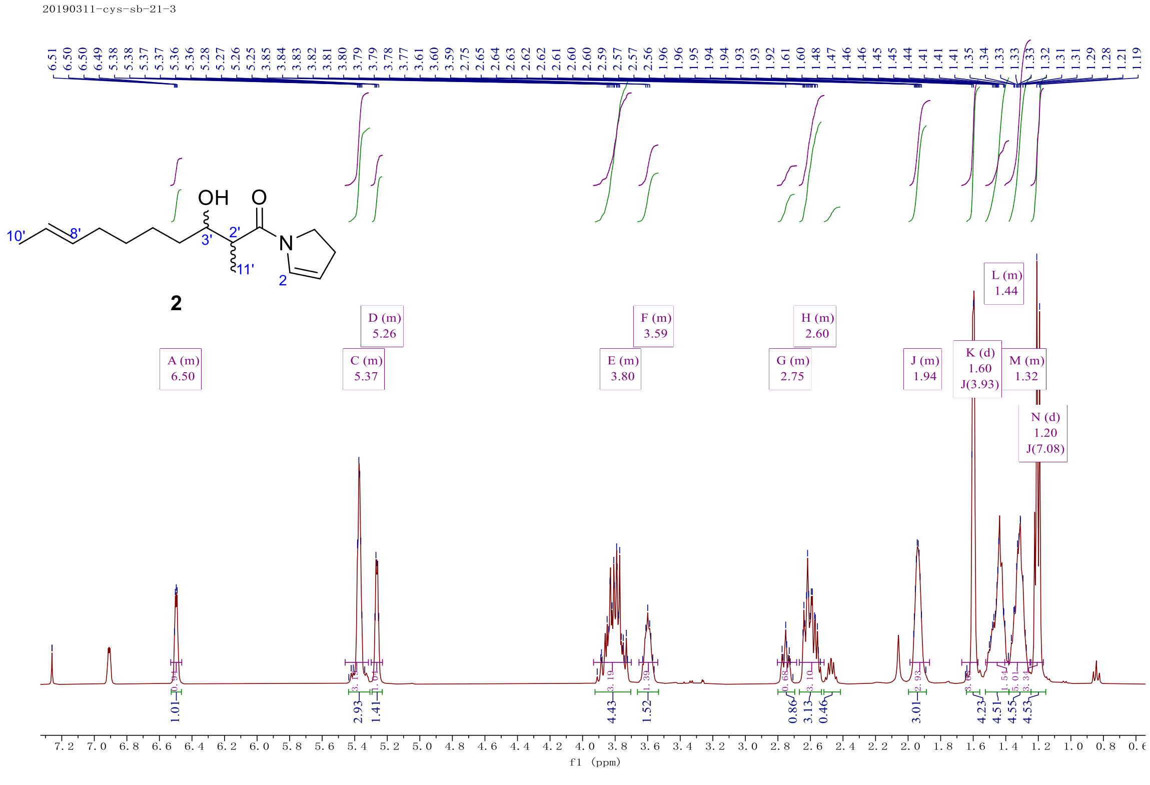

Figure S9 ^{13}C NMR spectrum of **2** (101 MHz, $CDCl_3$).

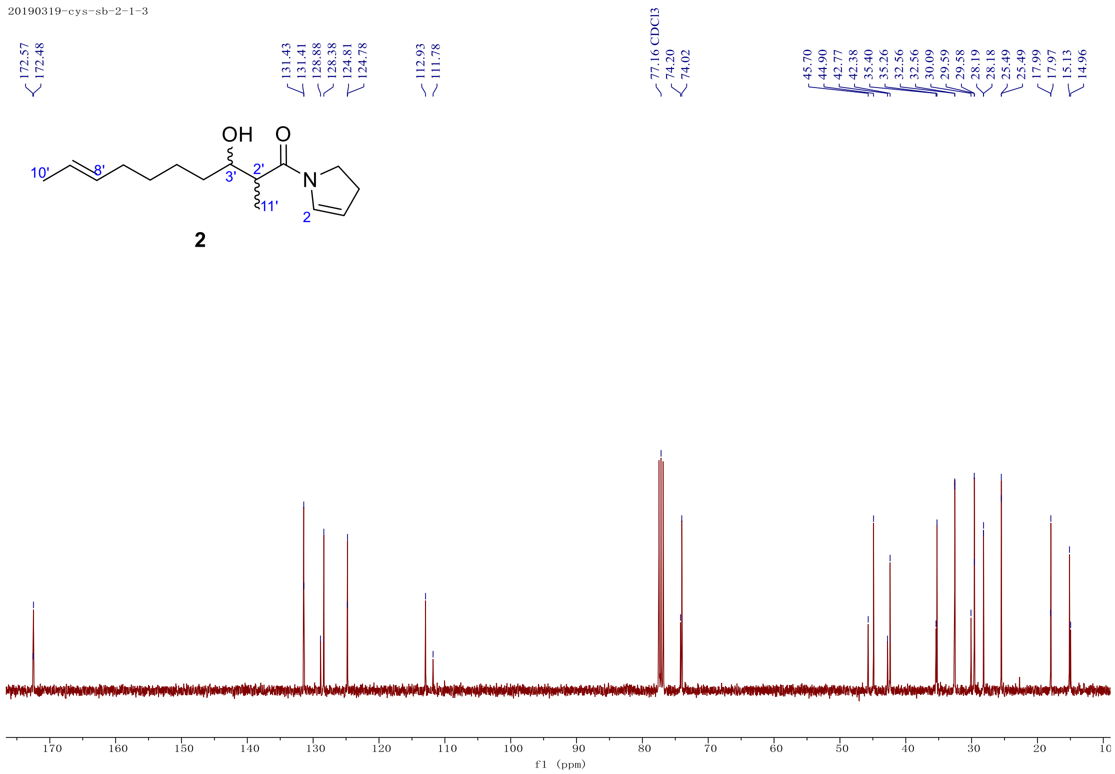

Figure S10 ^{1}H-^{1}H COSY spectrum of **2** (400 MHz, $CDCl_3$).

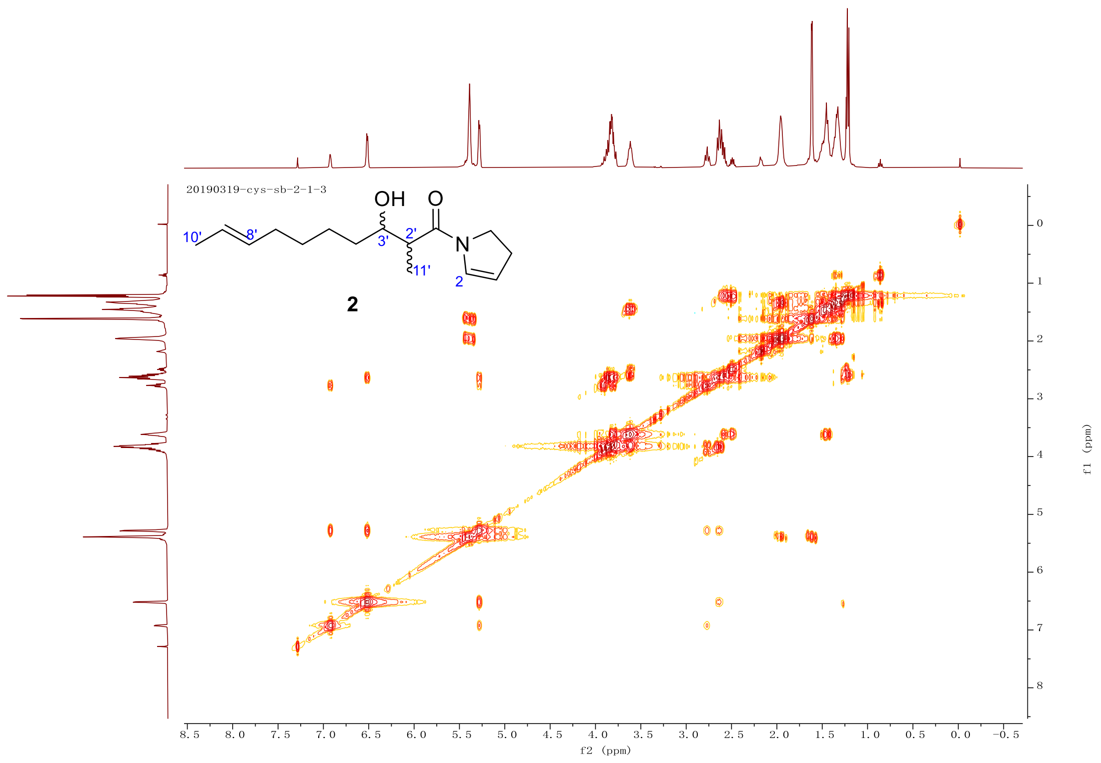

Figure S11 HSQC spectrum of **2** (400 MHz, $CDCl_3$).

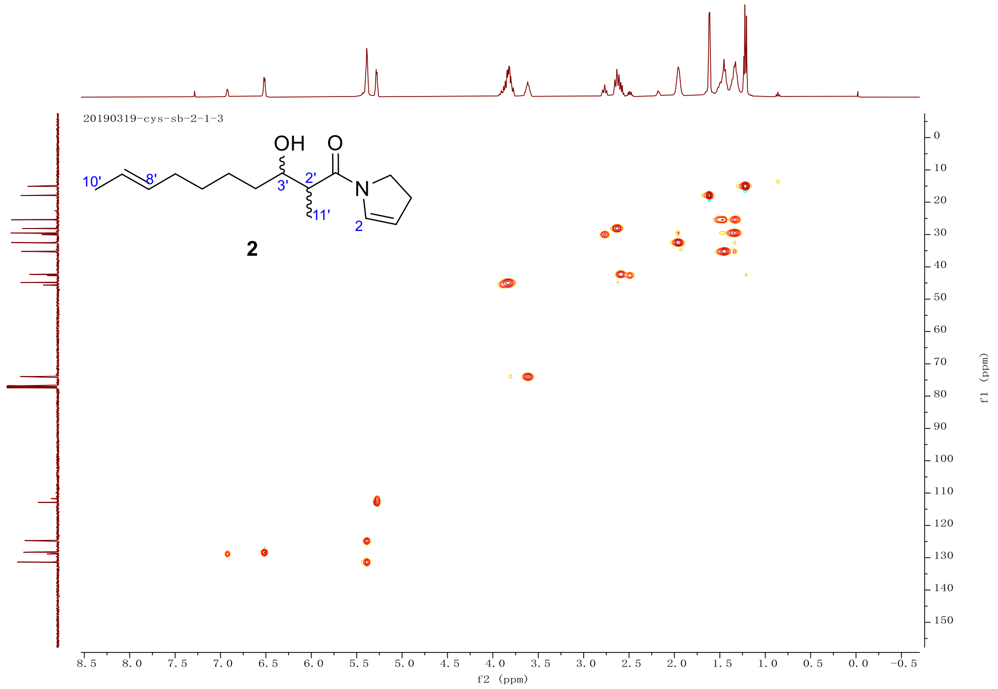

Figure S12 HMBC spectrum of **2** (400 MHz, $CDCl_3$).

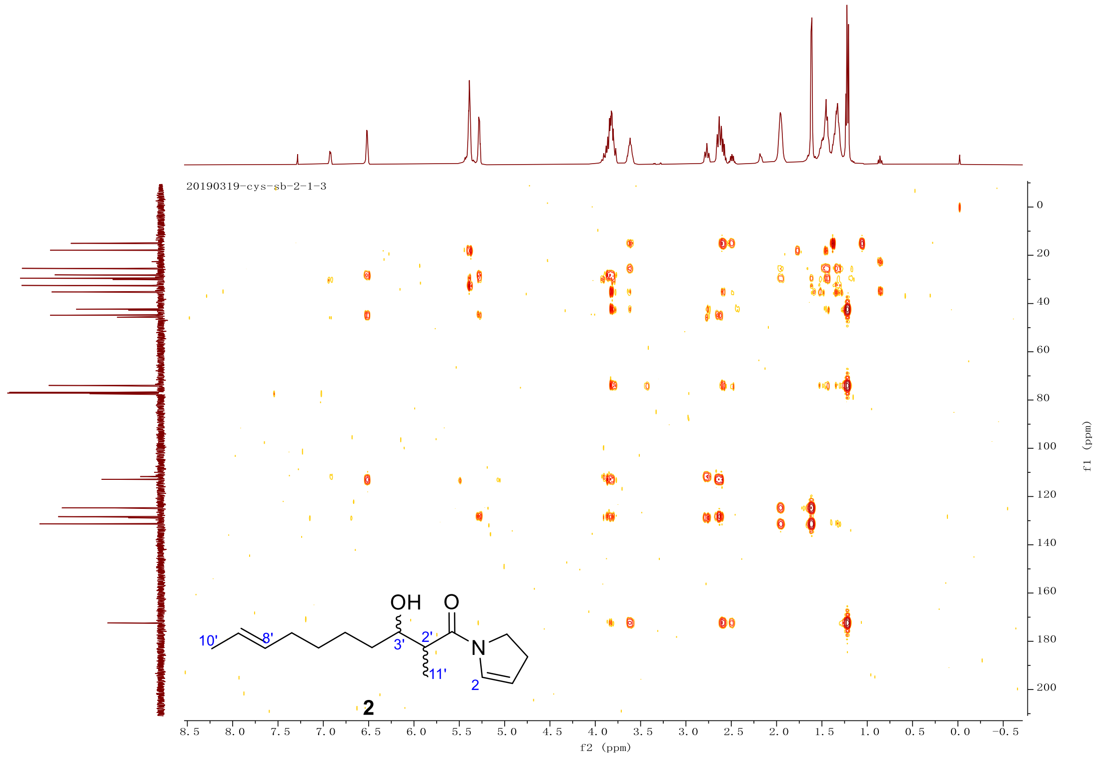

Figure S13 ^{1}H NMR spectrum of the (*S*)-MTPA (**2a,** green) and (*R*)-MTPA (**2b,** red) esters (600 MHz, pyr-d_6).

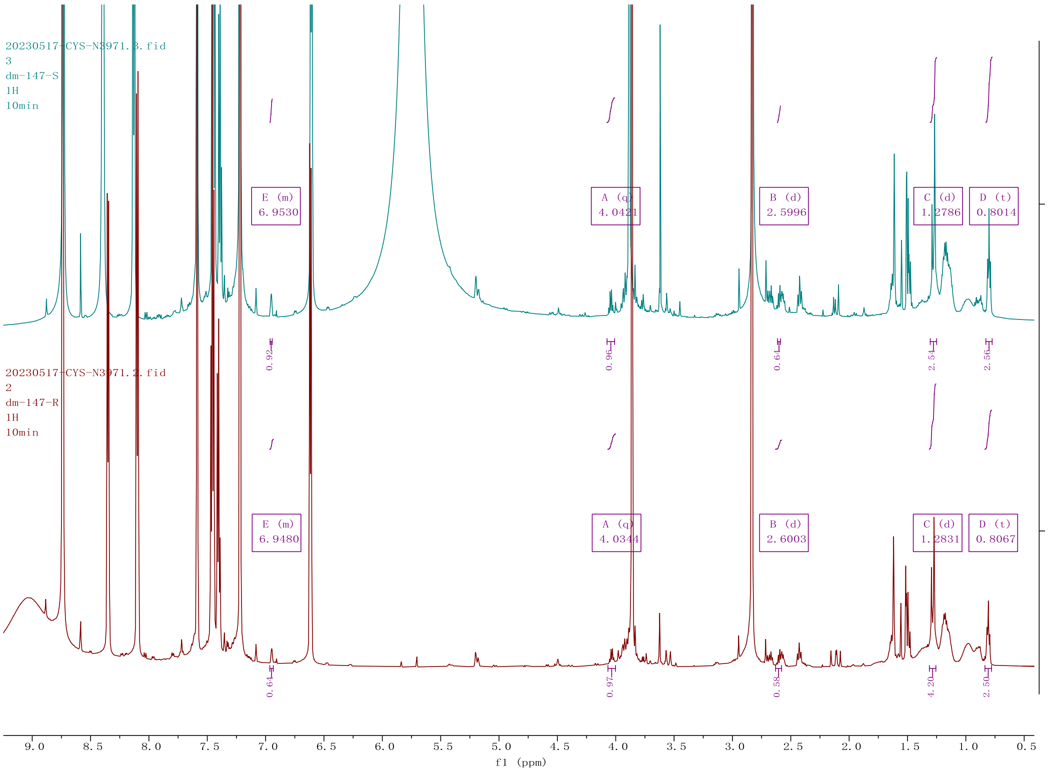

Figure S14 HRESIMS spectrum of **3**.

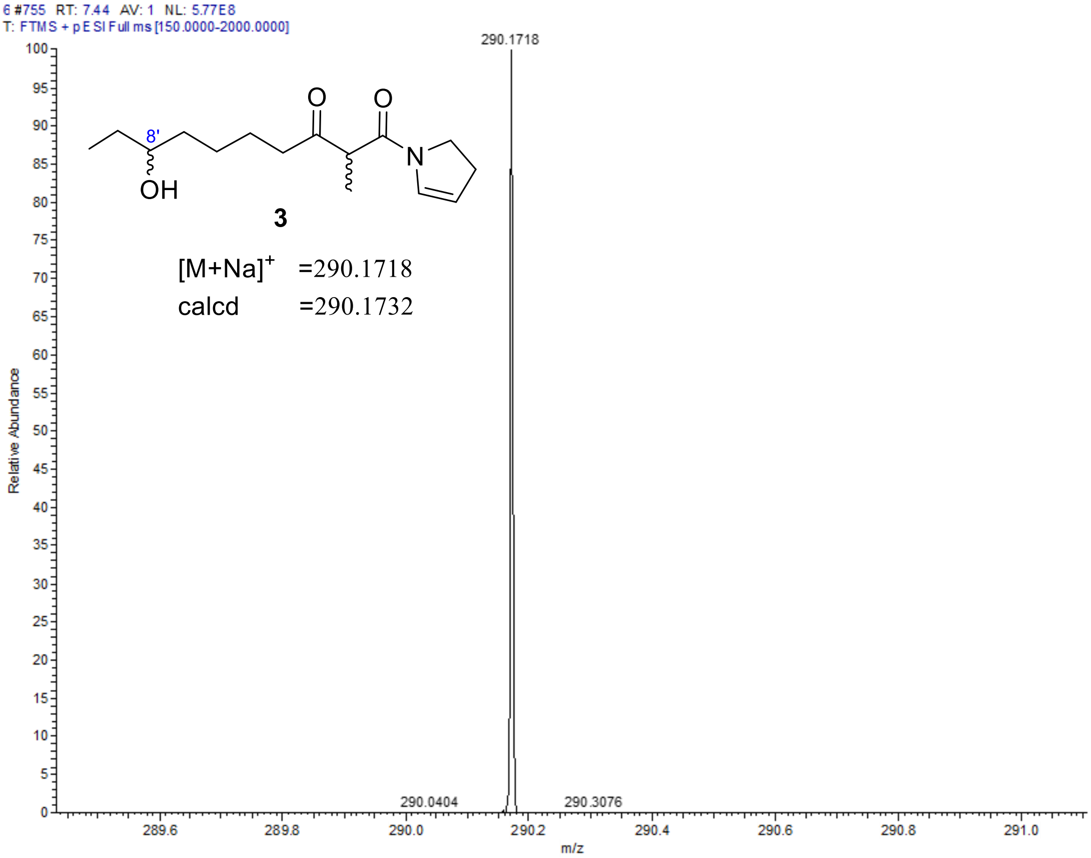

Figure S15 ^{1}H NMR spectrum of **3** (400 MHz, $CDCl_3$).

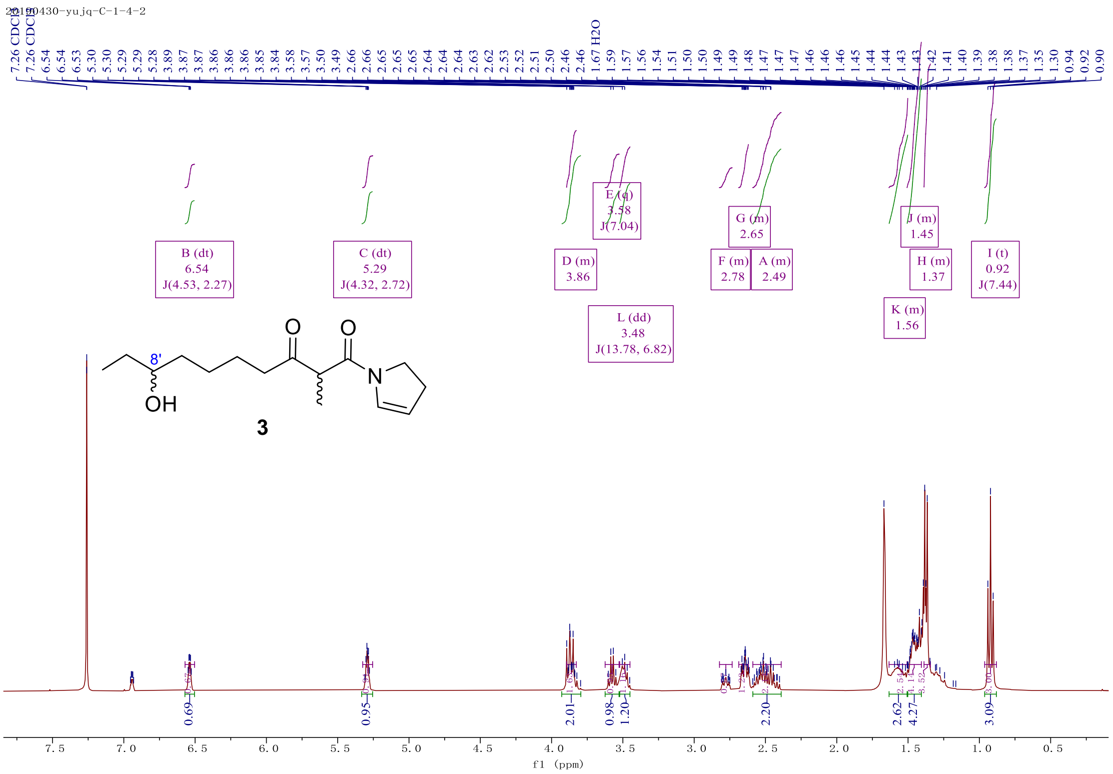

Figure S16 ^{13}C NMR spectrum of **3** (101 MHz, $CDCl_3$).

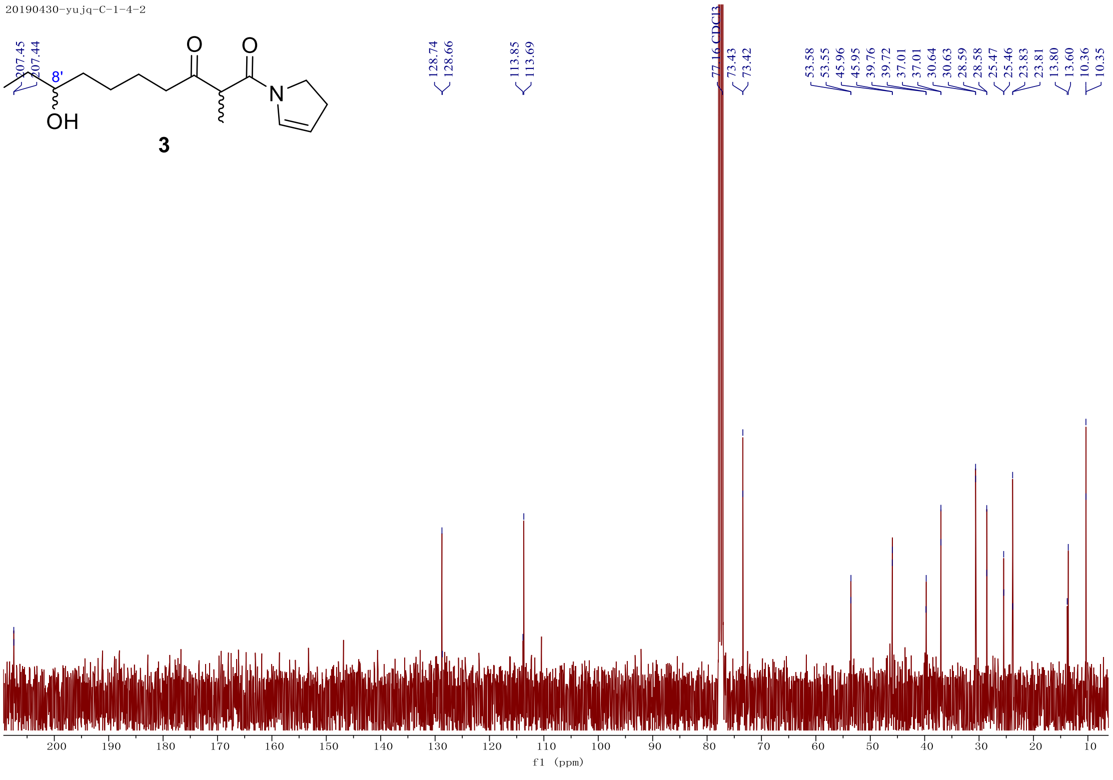

Figure S17 ^{1}H-^{1}H COSY spectrum of **3** (400 MHz, $CDCl_3$).

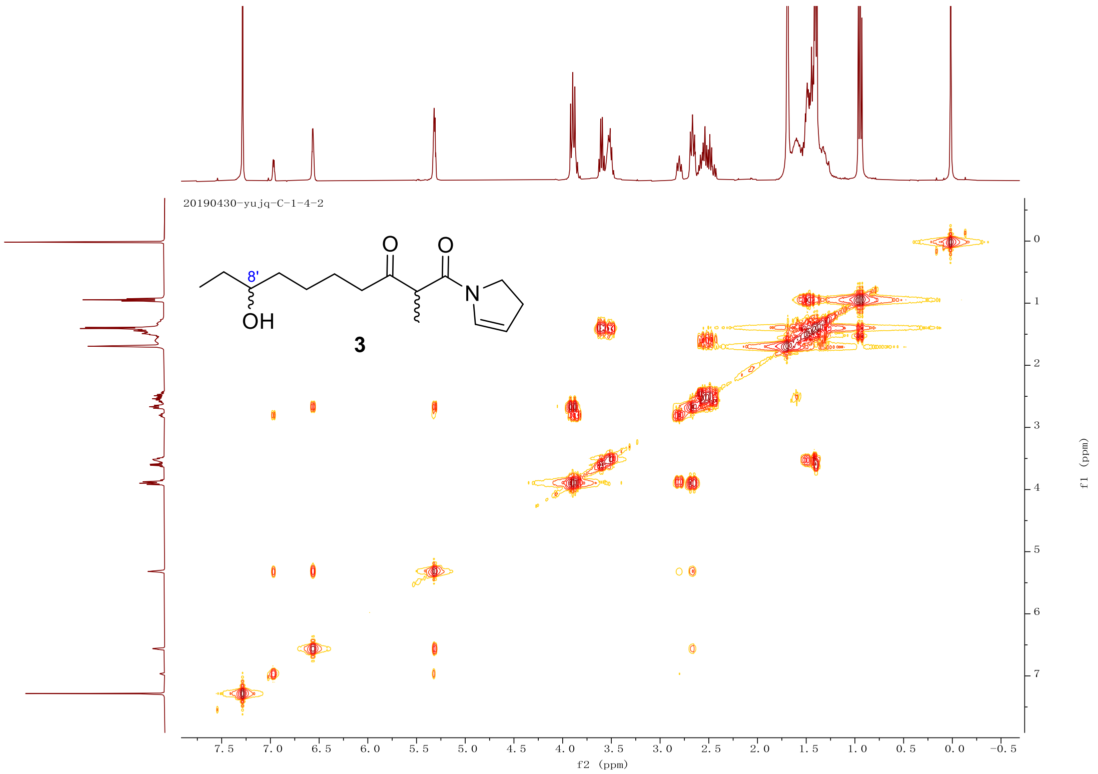

Figure S18 HSQC spectrum of **3** (400 MHz, $CDCl_3$).

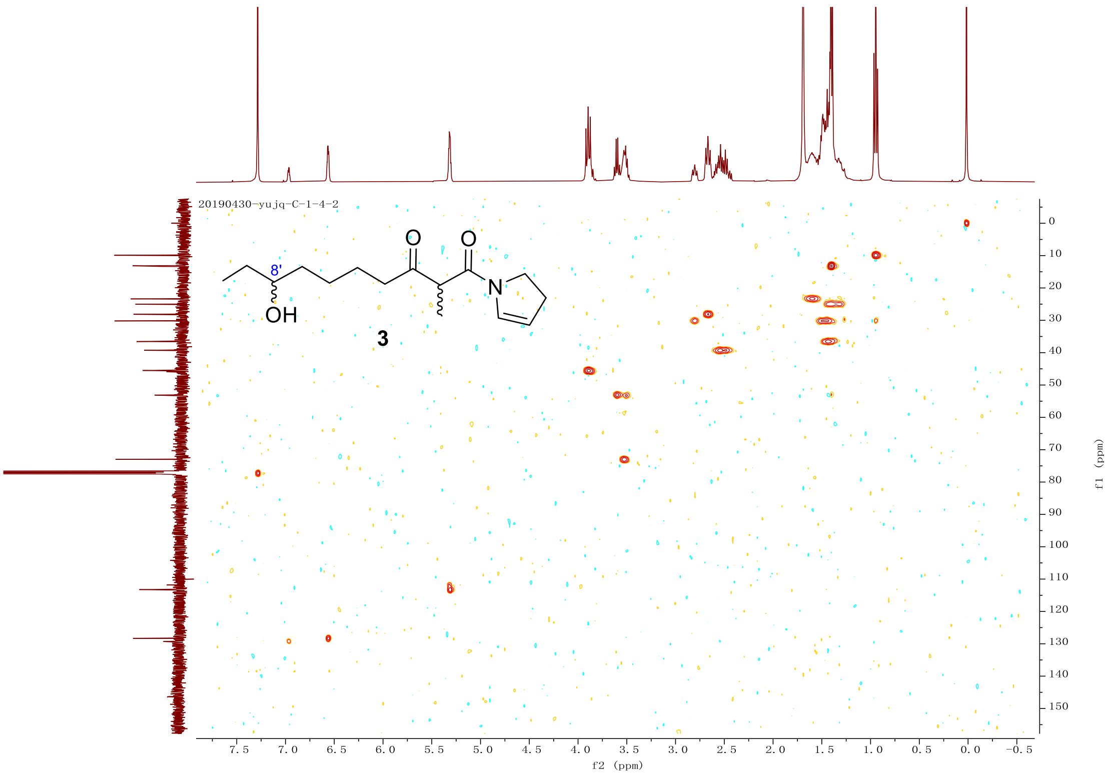

Figure S19 HMBC spectrum of **3** (400 MHz, $CDCl_3$).

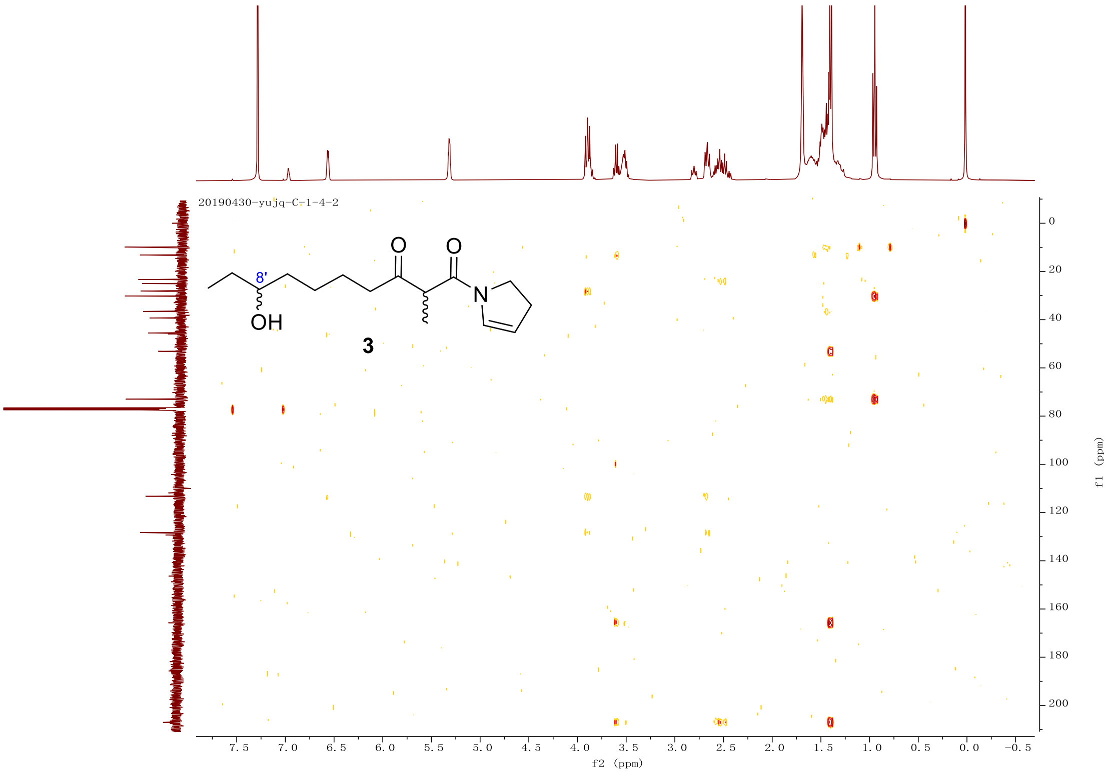

Figure S20 HRESIMS spectrum of **4**.

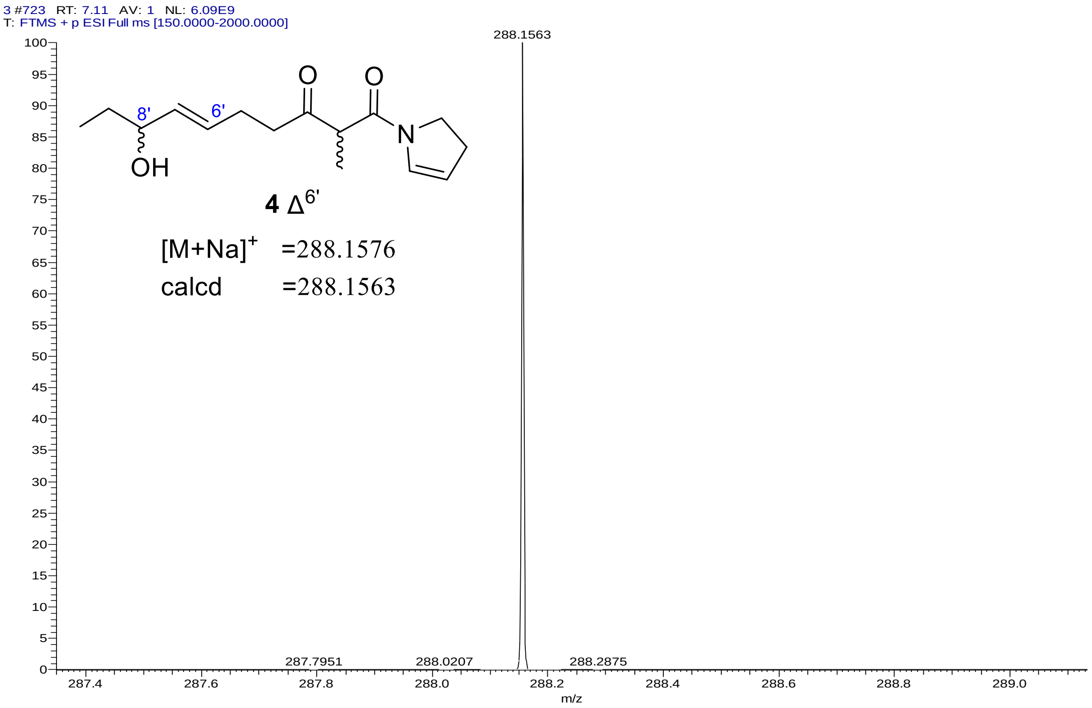

Figure S21 ^{1}H NMR spectrum of **4** (400 MHz, $CDCl_3$).

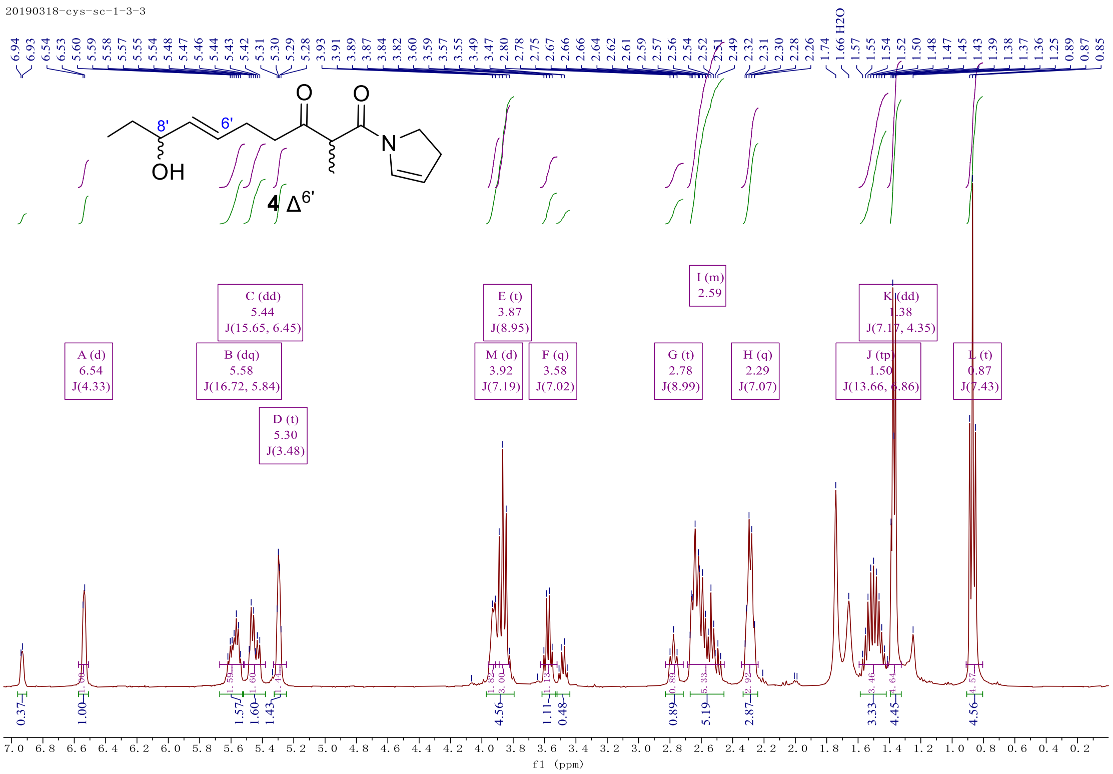

Figure S22 ^{13}C NMR spectrum of **4** (101 MHz, $CDCl_3$).

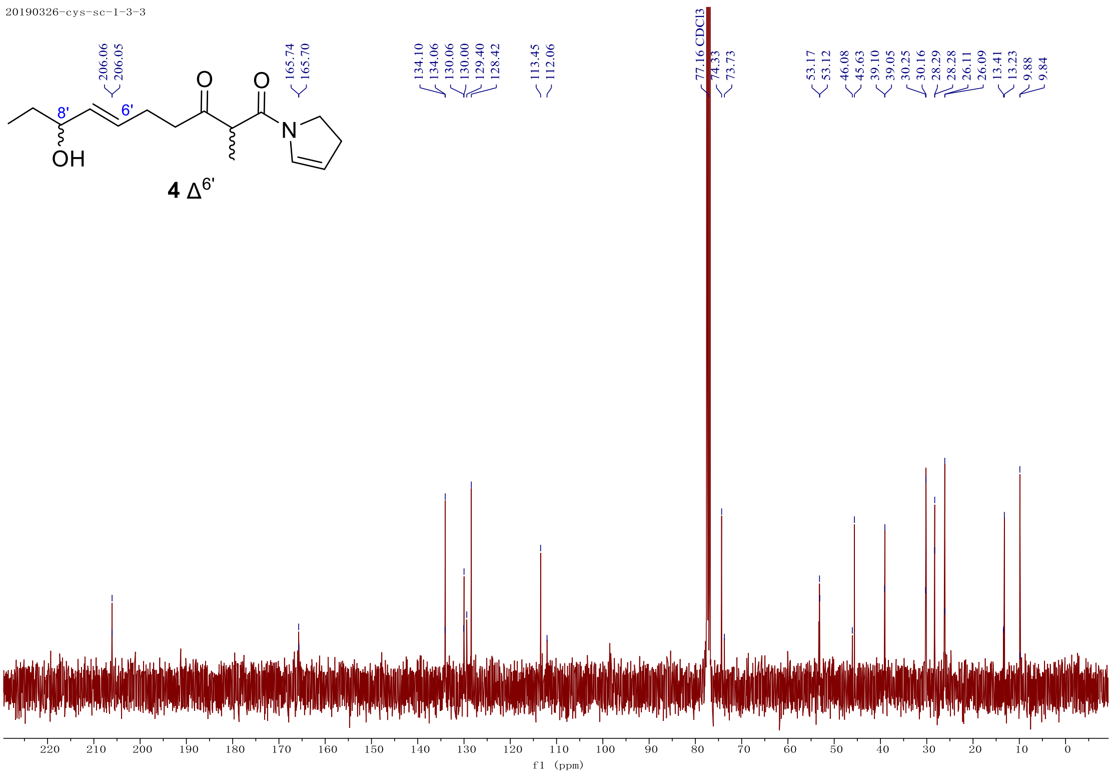

Figure S23 ^{1}H-^{1}H COSY spectrum of **4** (400 MHz, $CDCl_3$).

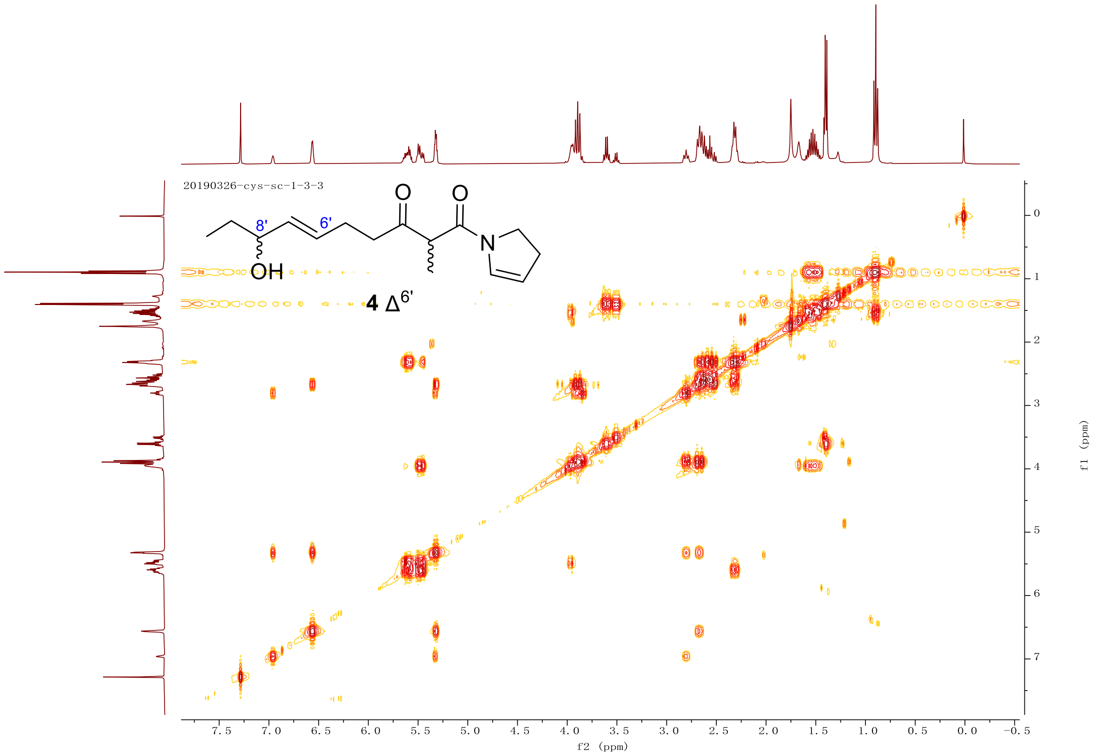

Figure S24 HSQC spectrum of **4** (400 MHz, $CDCl_3$).

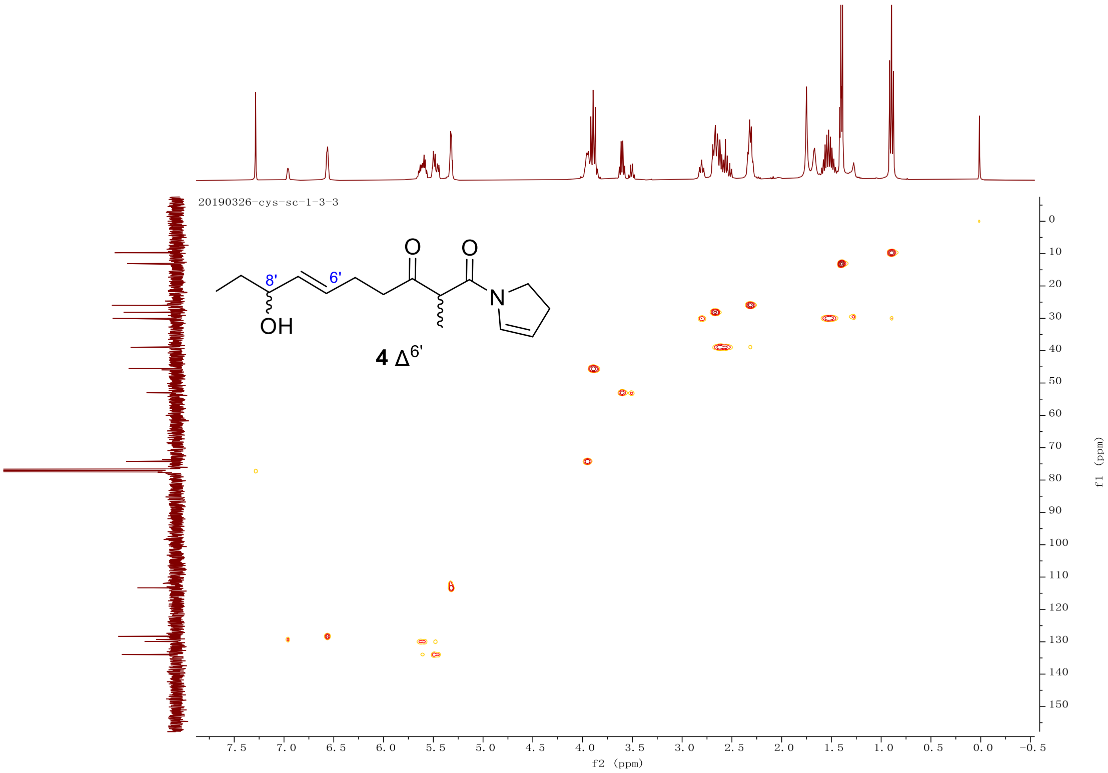

Figure S25 HMBC spectrum of **4** (400 MHz, $CDCl_3$).

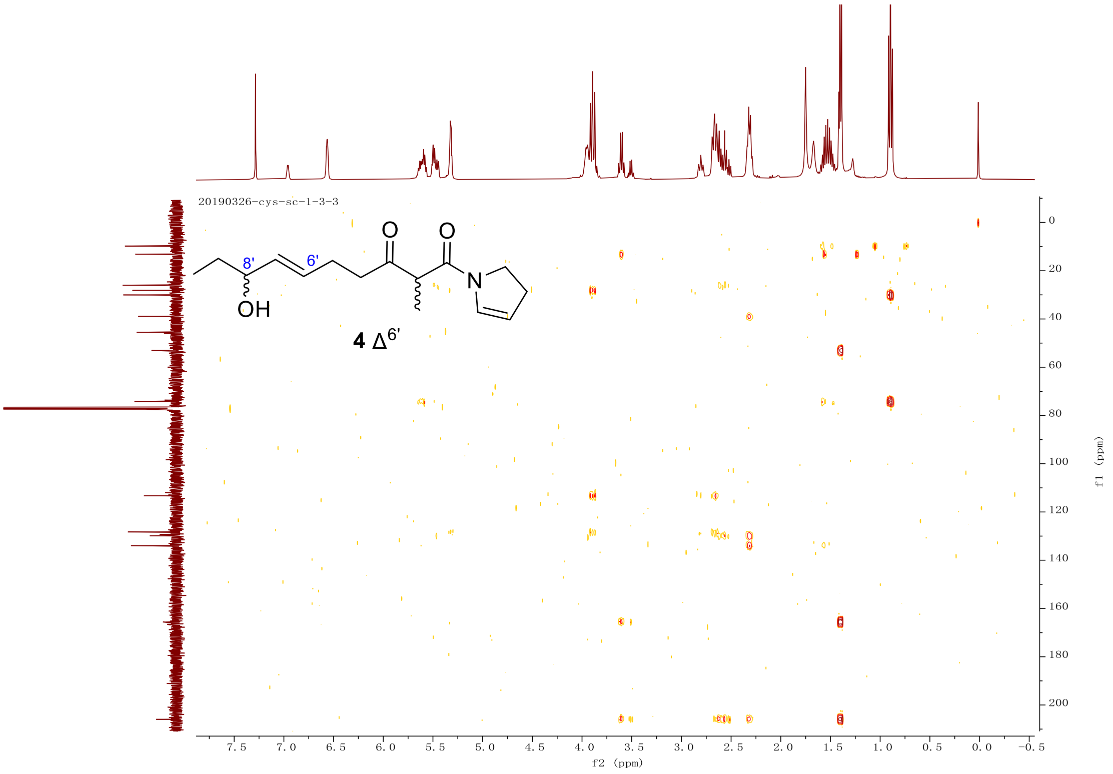

Figure S26 HRESIMS spectrum of **5**.

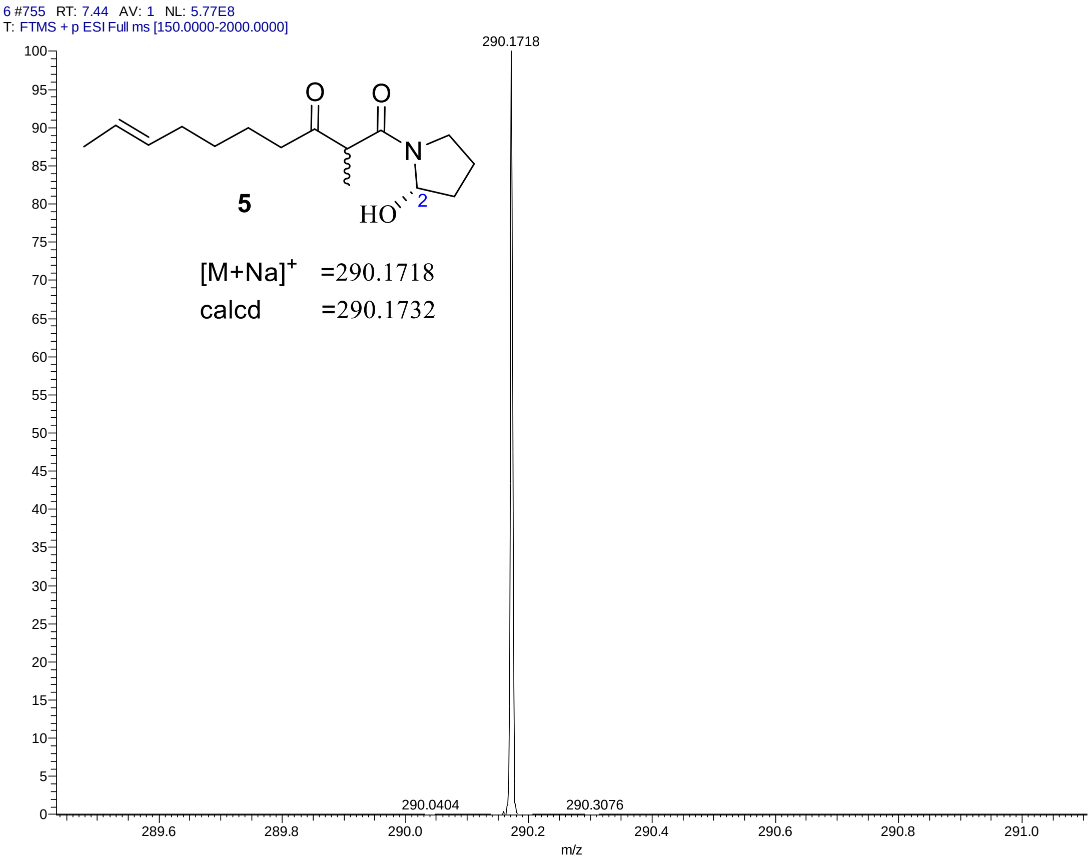

Figure S27 ^{1}H NMR spectrum of **5** (400 MHz, $CDCl_3$).

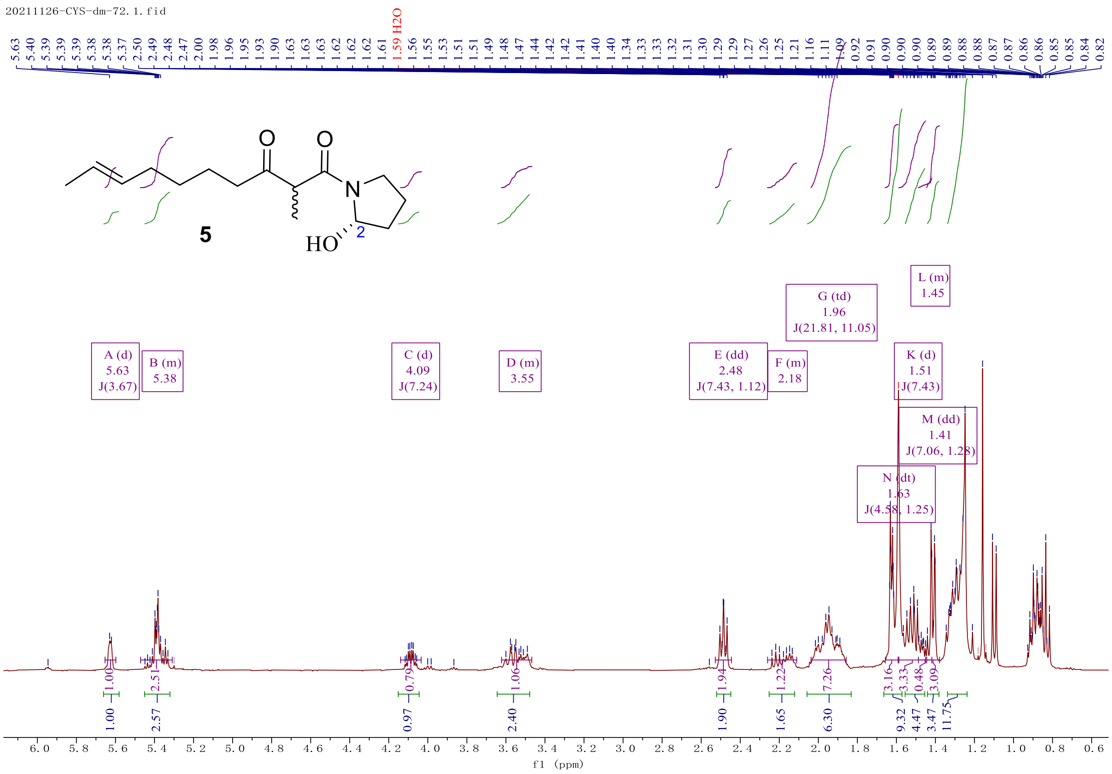

Figure S28 ^{13}C NMR spectrum of **5** (101 MHz, $CDCl_3$).

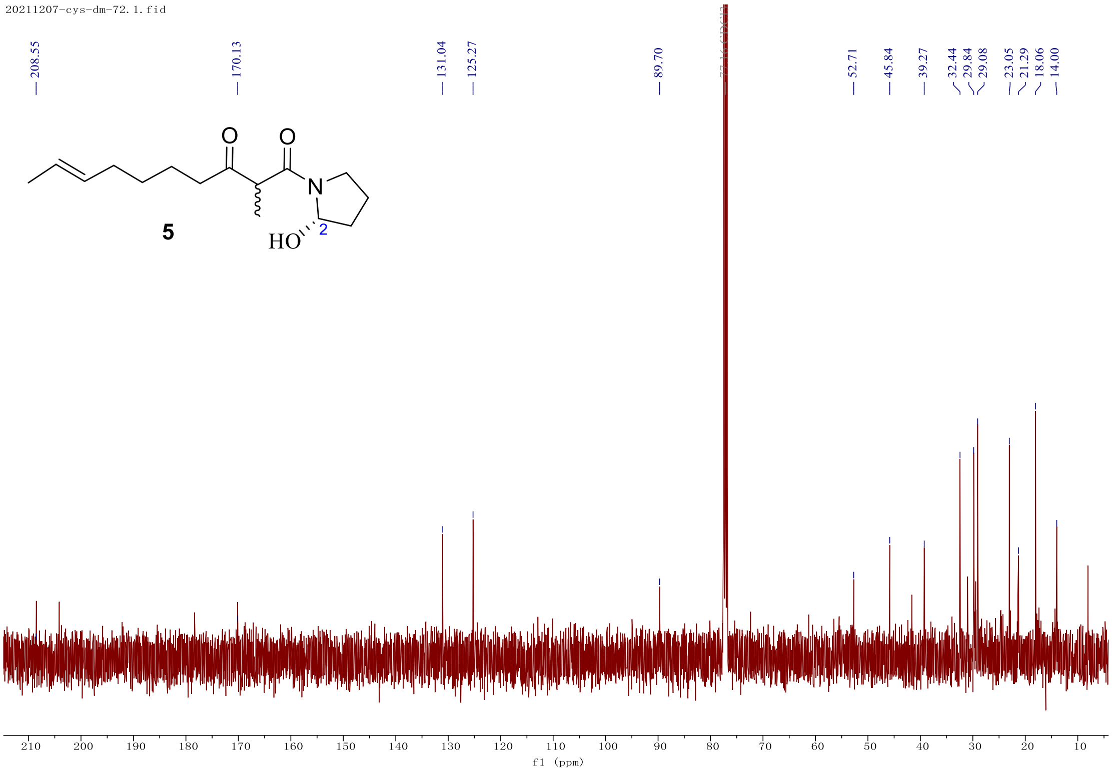

Figure S29 ^{1}H-^{1}H COSY spectrum of **5** (400 MHz, $CDCl_3$).

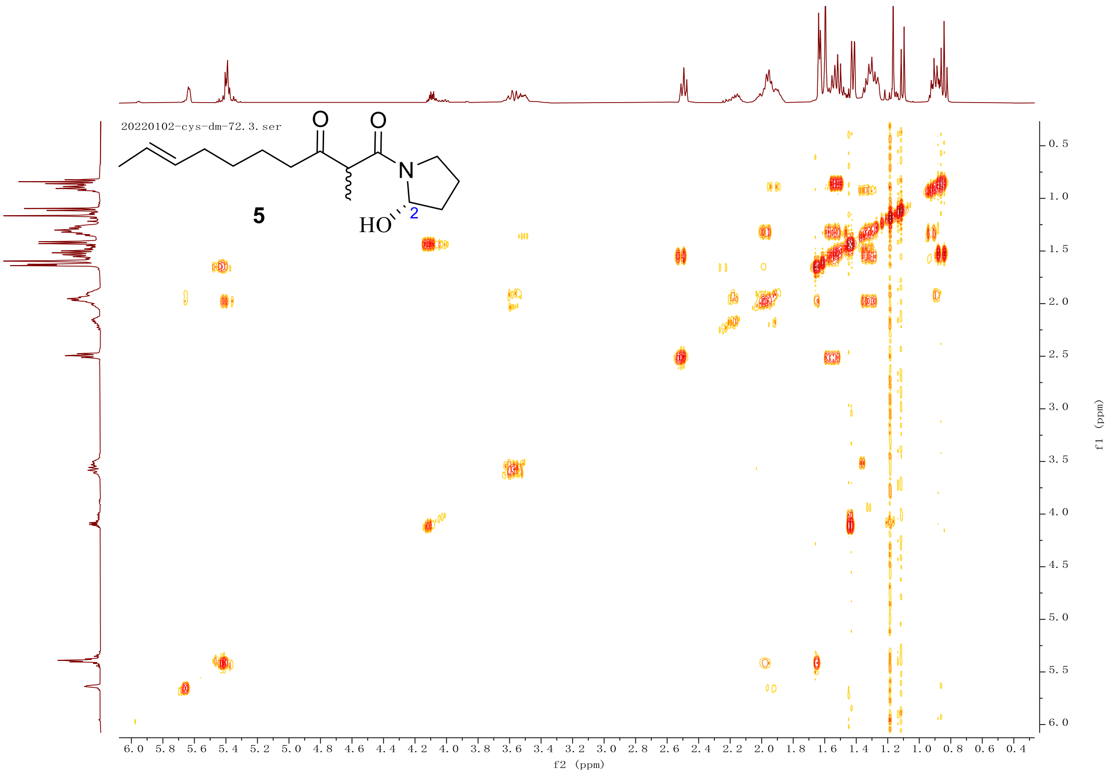

Figure S30 HSQC spectrum of **5** (400 MHz, $CDCl_3$).

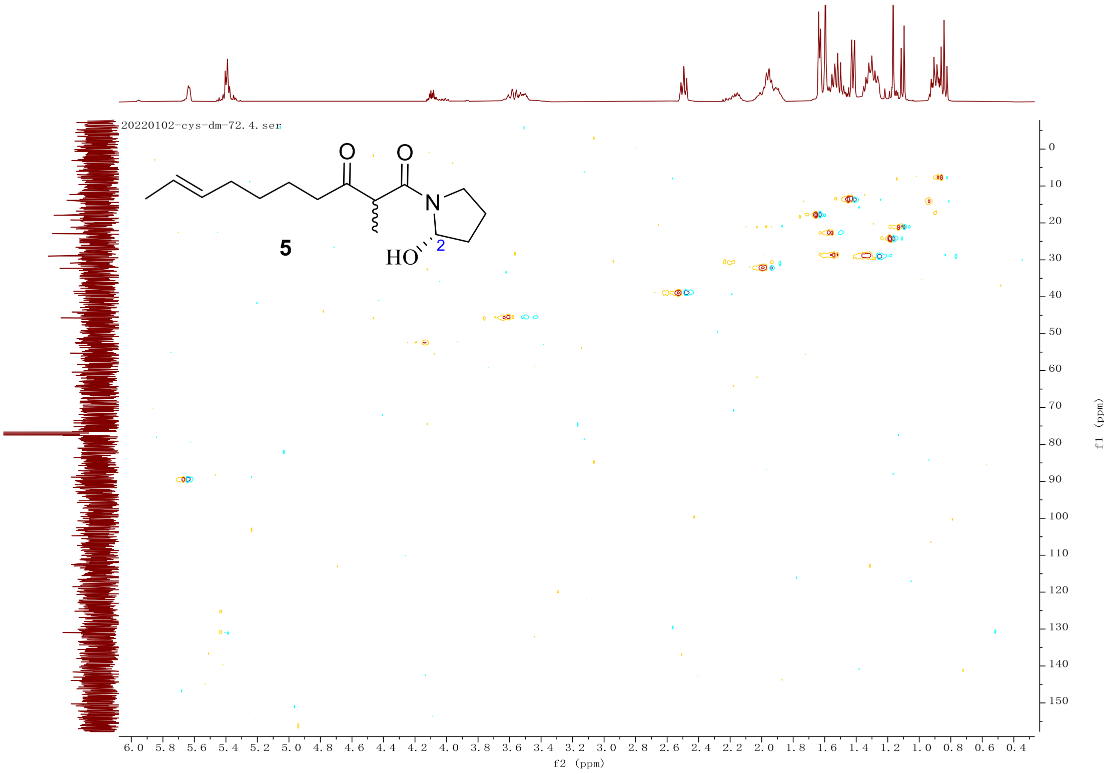

Figure S31 HMBC spectrum of **5** (400 MHz, $CDCl_3$).

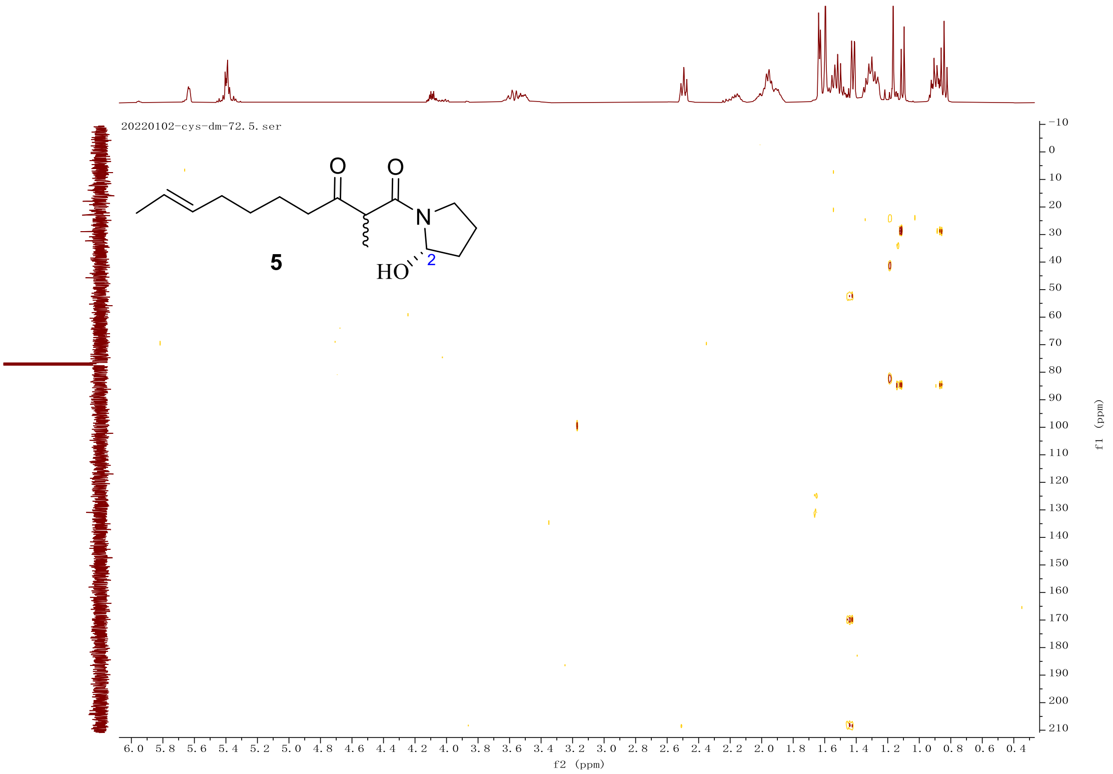

Figure S32 HRESIMS spectrum of **6**.

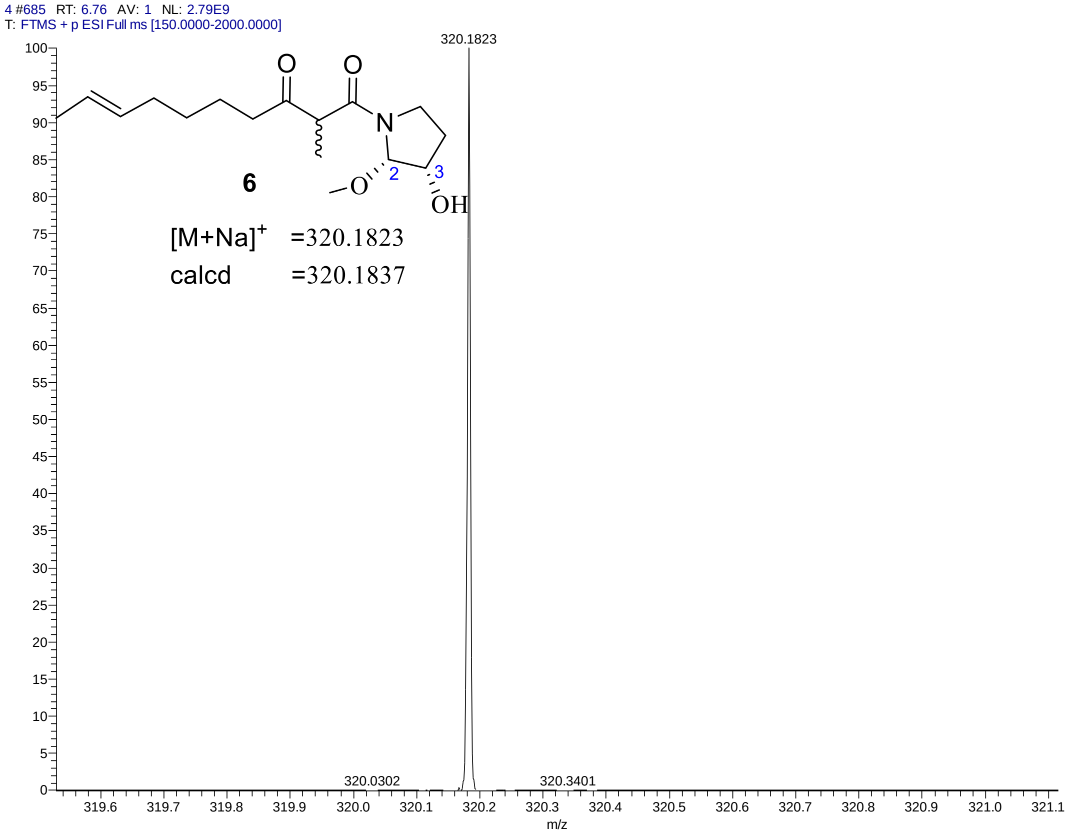

Figure S33 ^{1}H NMR spectrum of **6** (400 MHz, $CDCl_3$).

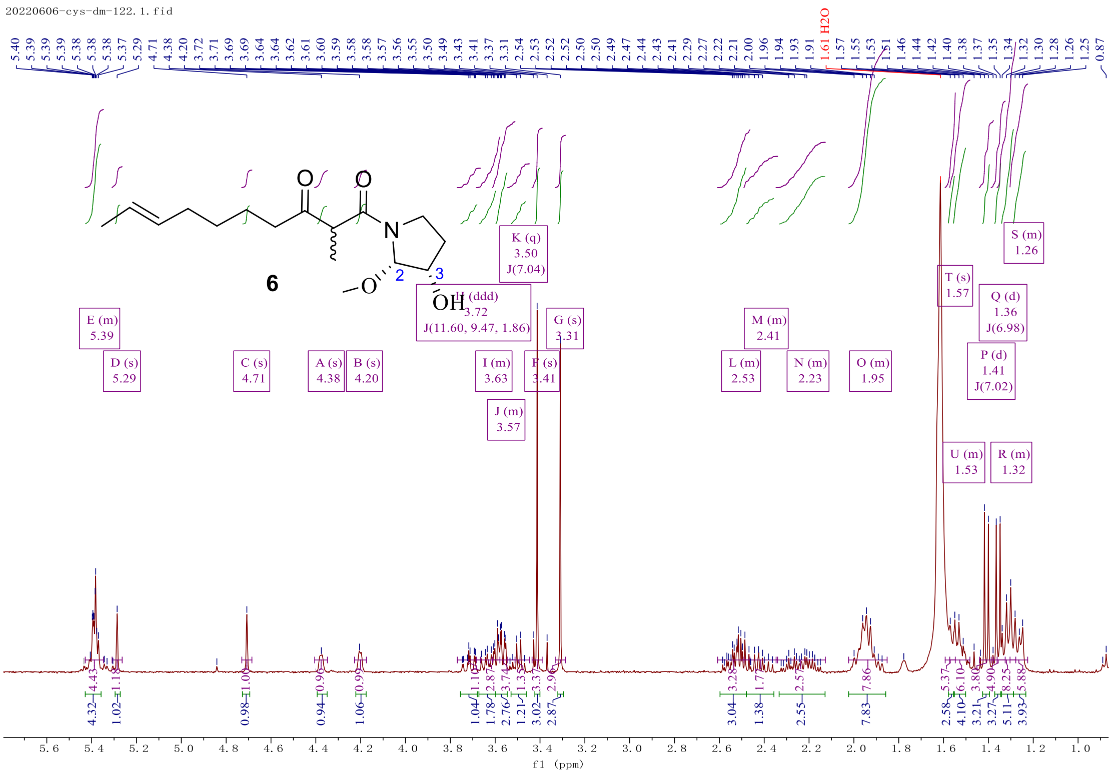

Figure S34 ^{13}C NMR spectrum of **6** (101 MHz, $CDCl_3$).

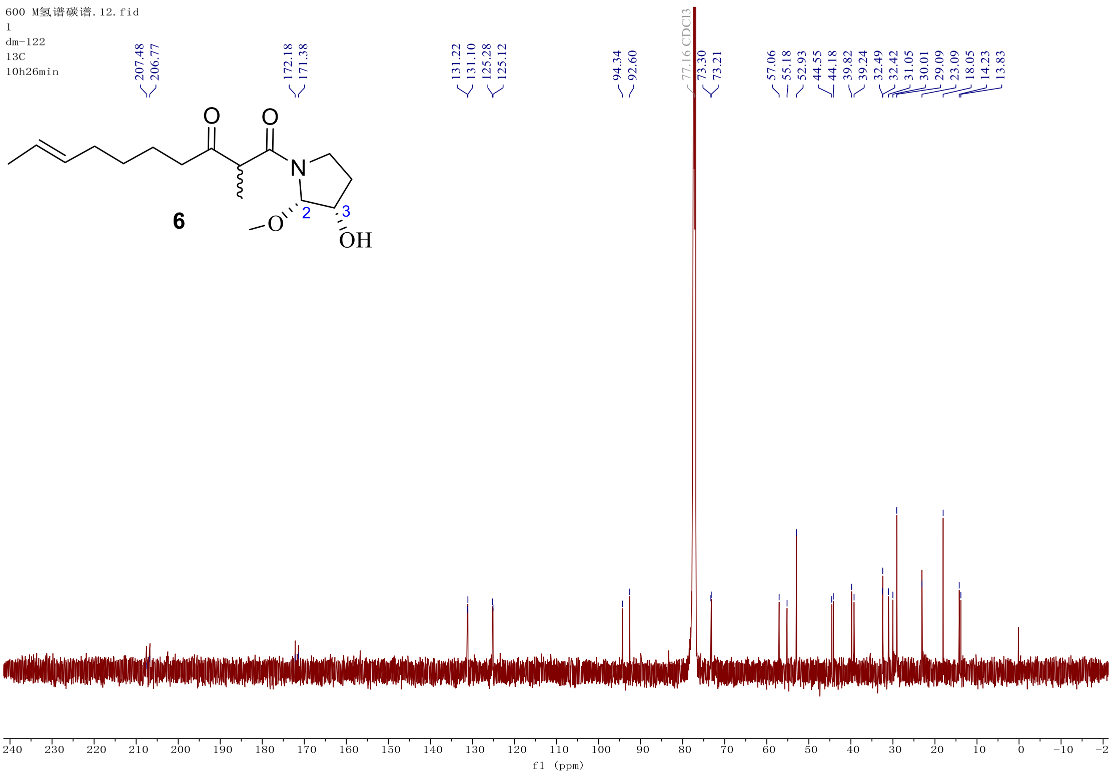

Figure S35 ^{1}H-^{1}H COSY spectrum of **6** (400 MHz, CDCl$_3$).

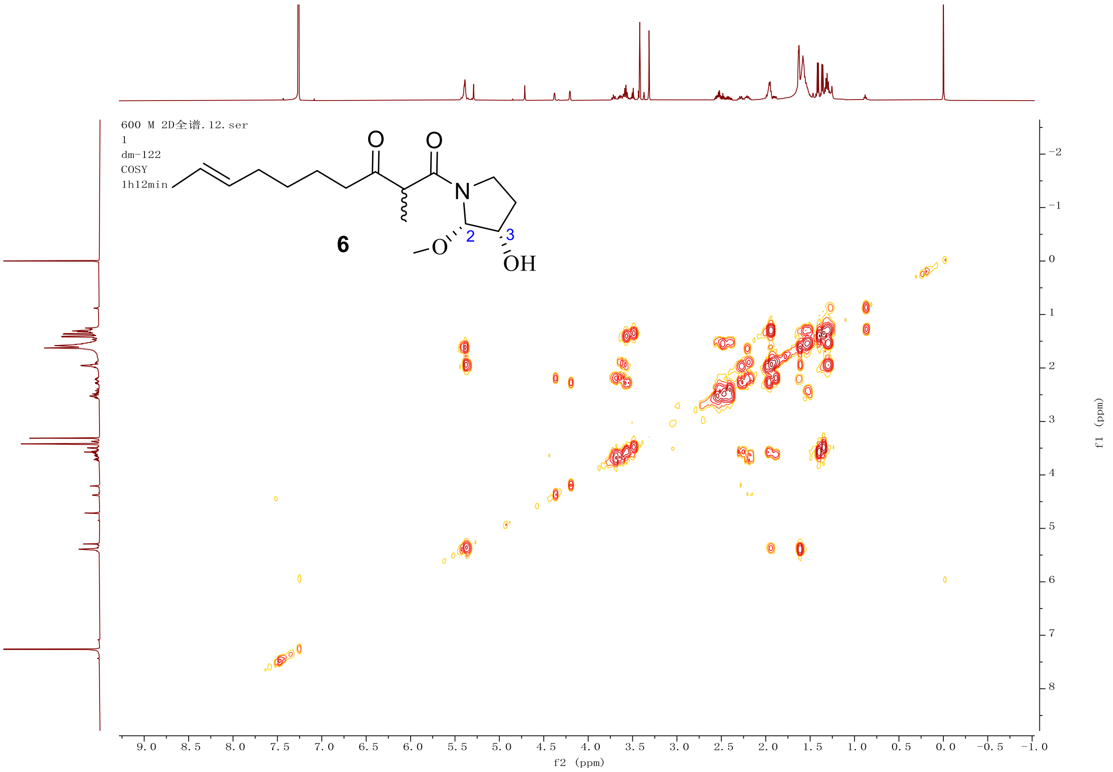

Figure S36 HSQC spectrum of **6** (400 MHz, CDCl$_3$).

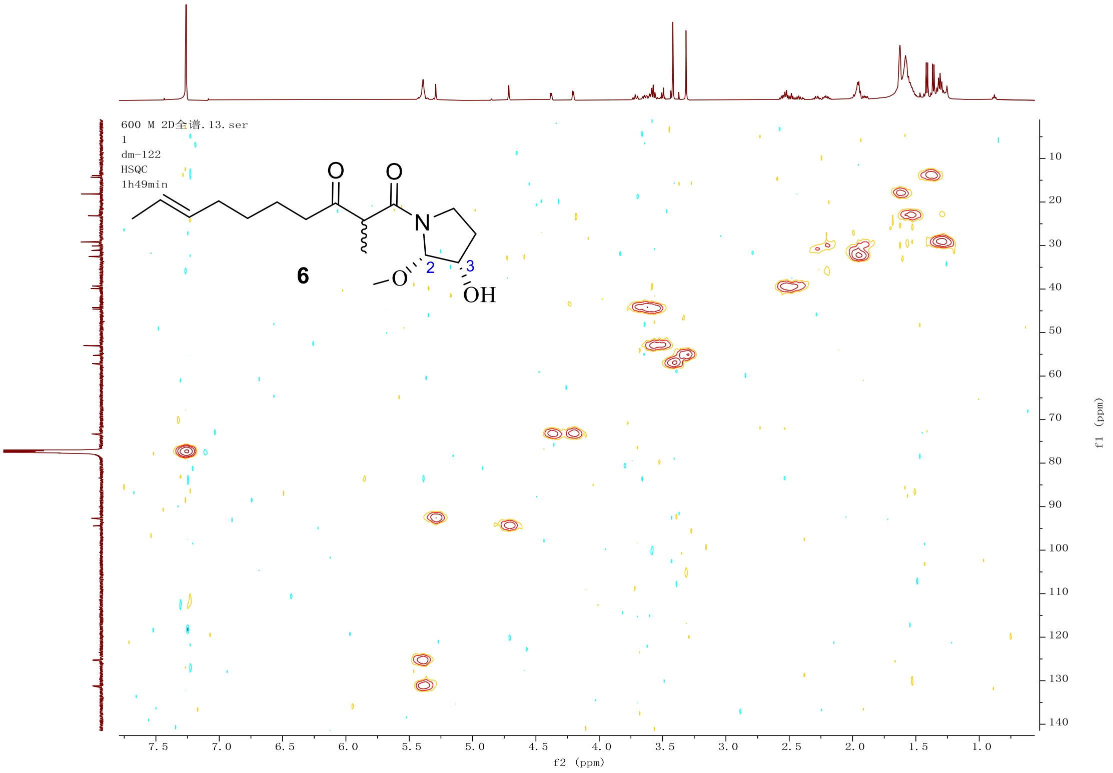

Figure S37 HMBC spectrum of **6** (400 MHz, $CDCl_3$).

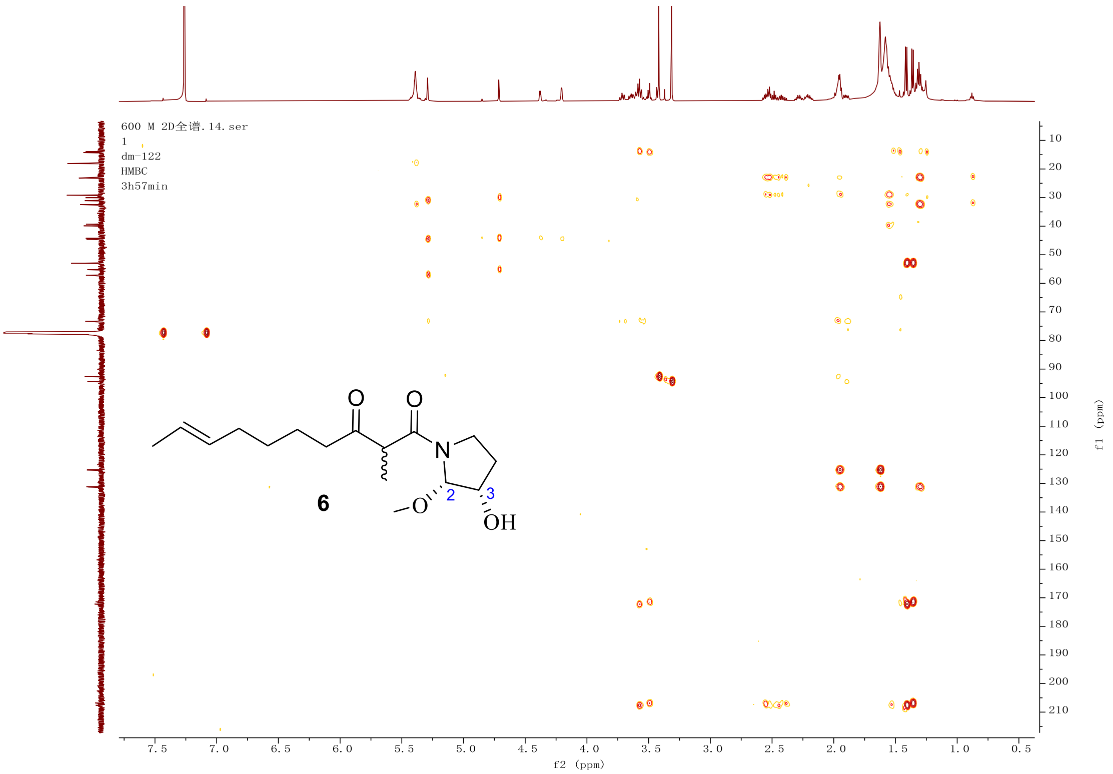

Figure S38 NOESY spectrum of **6** (400 MHz, $CDCl_3$).

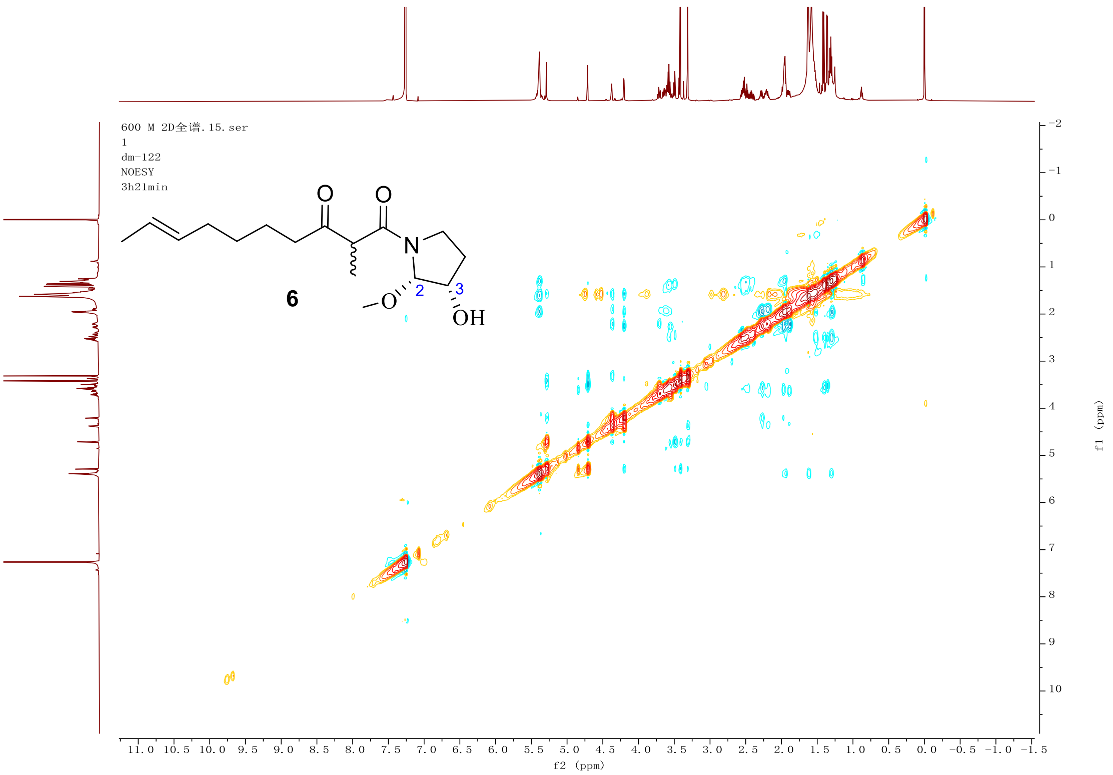

Figure S39 HRESIMS spectrum of **7**.

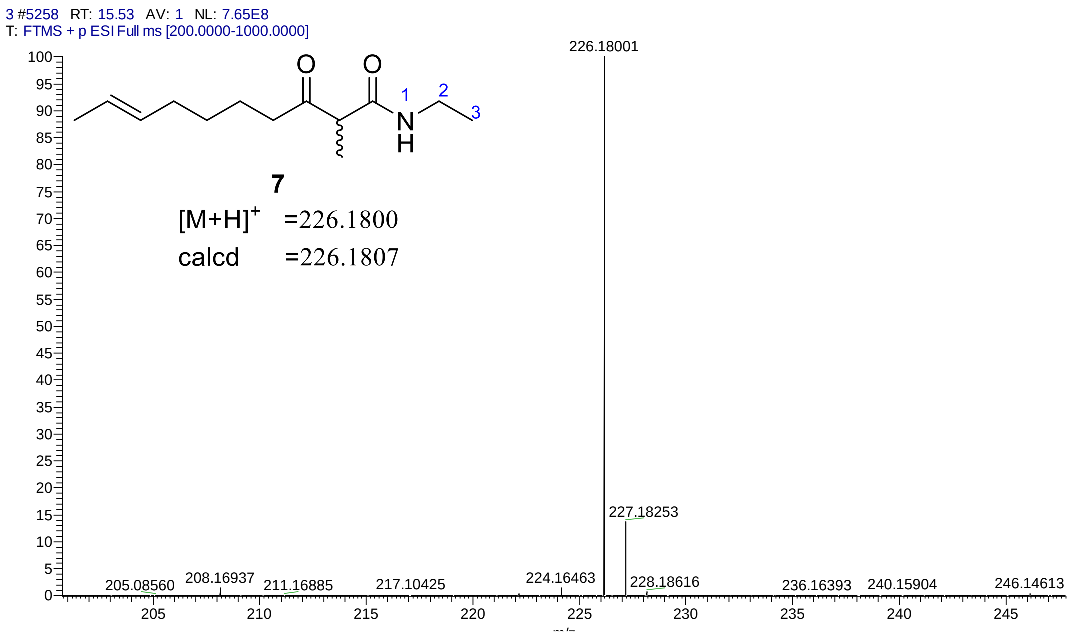

Figure S40 ^{1}H NMR spectrum of **7** (400 MHz, $CDCl_3$).

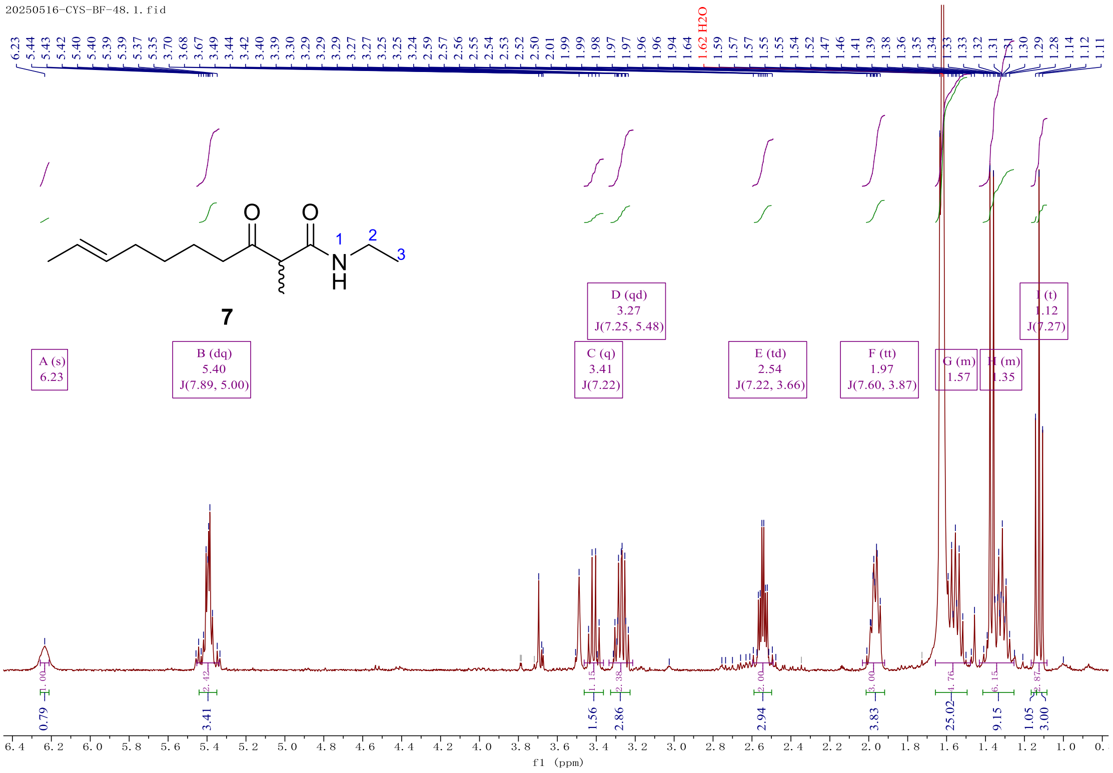

Figure S41 ^{13}C NMR spectrum of **7** (151 MHz, $CDCl_3$).

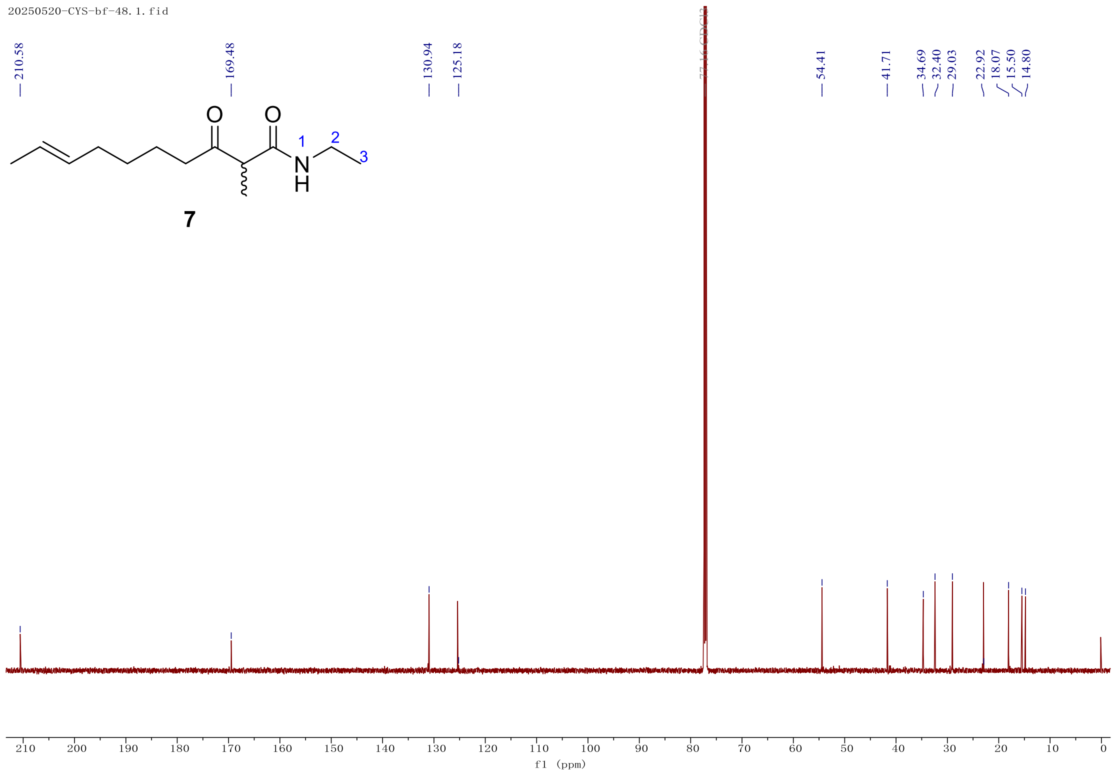

Figure S42 ^{1}H-^{1}H COSY spectrum of **7** (600 MHz, $CDCl_3$).

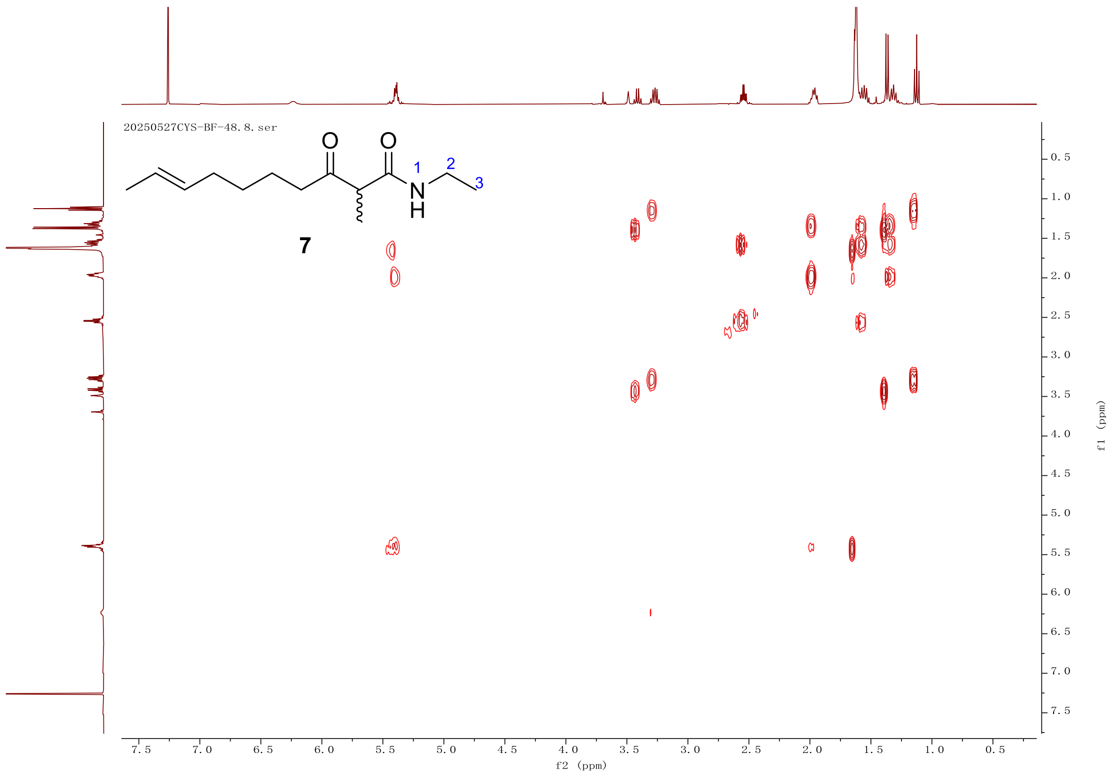

Figure S43 HSQC spectrum of **7** (600 MHz, $CDCl_3$).

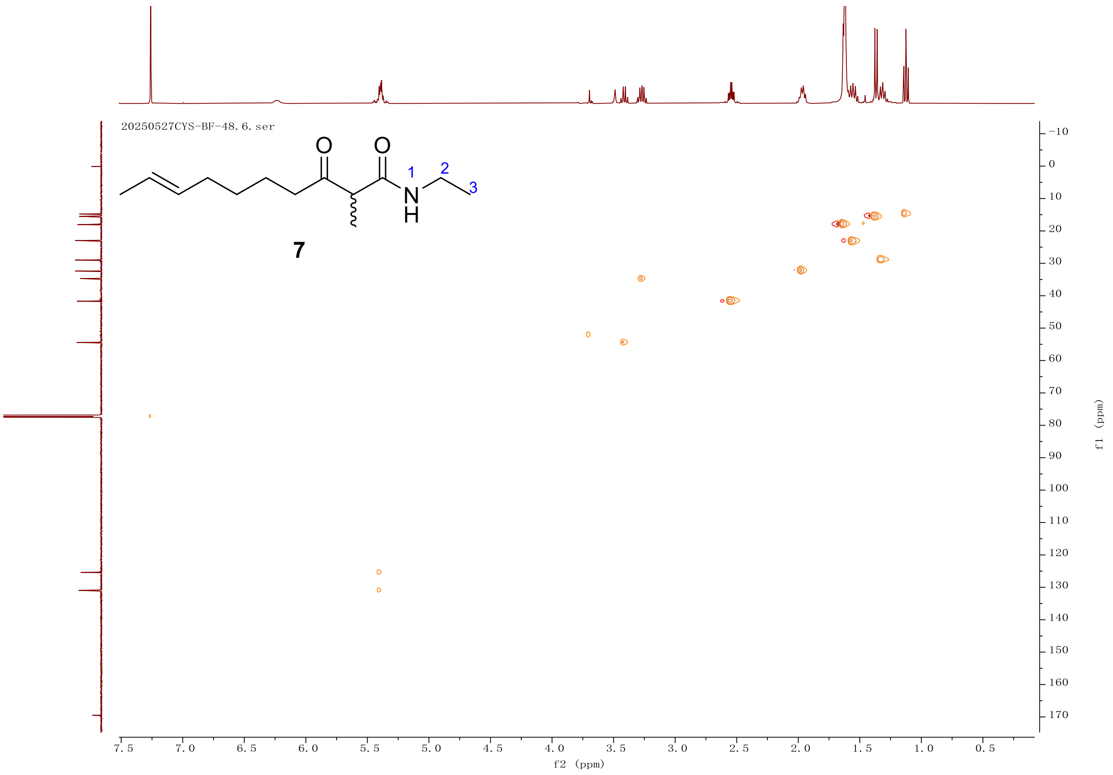

Figure S44 HMBC spectrum of **7** (600 MHz, $CDCl_3$).

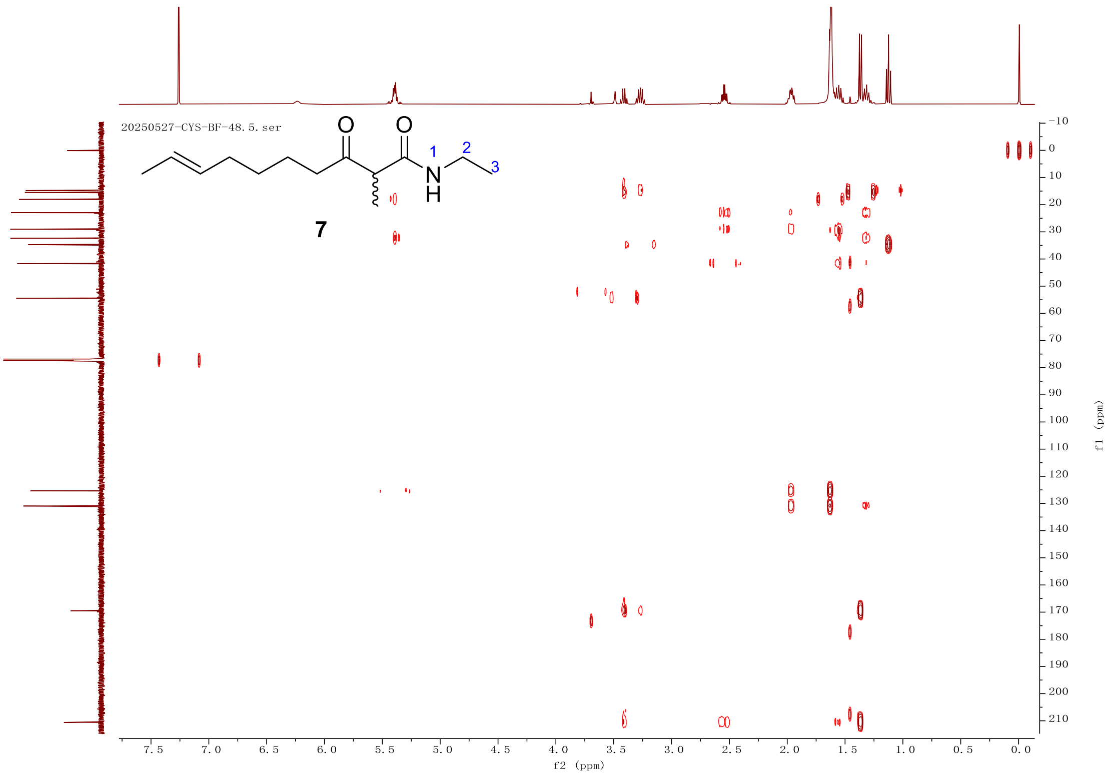

Figure S45 HRESIMS spectrum of **8**.

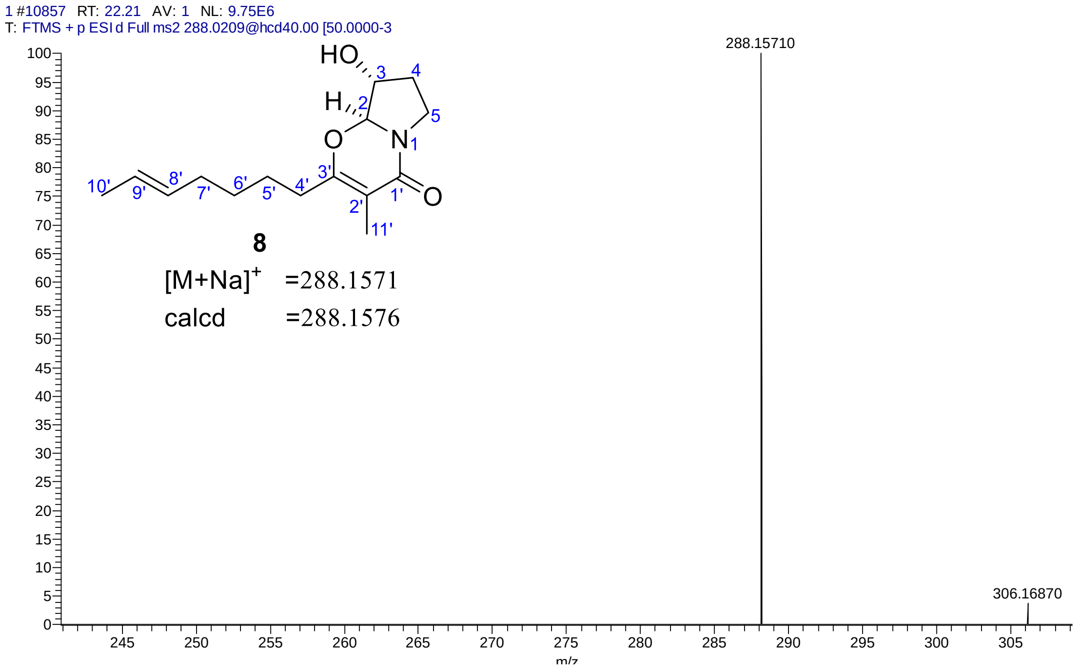

Figure S46 ^{1}H NMR spectrum of **8** (400 MHz, $CDCl_3$).

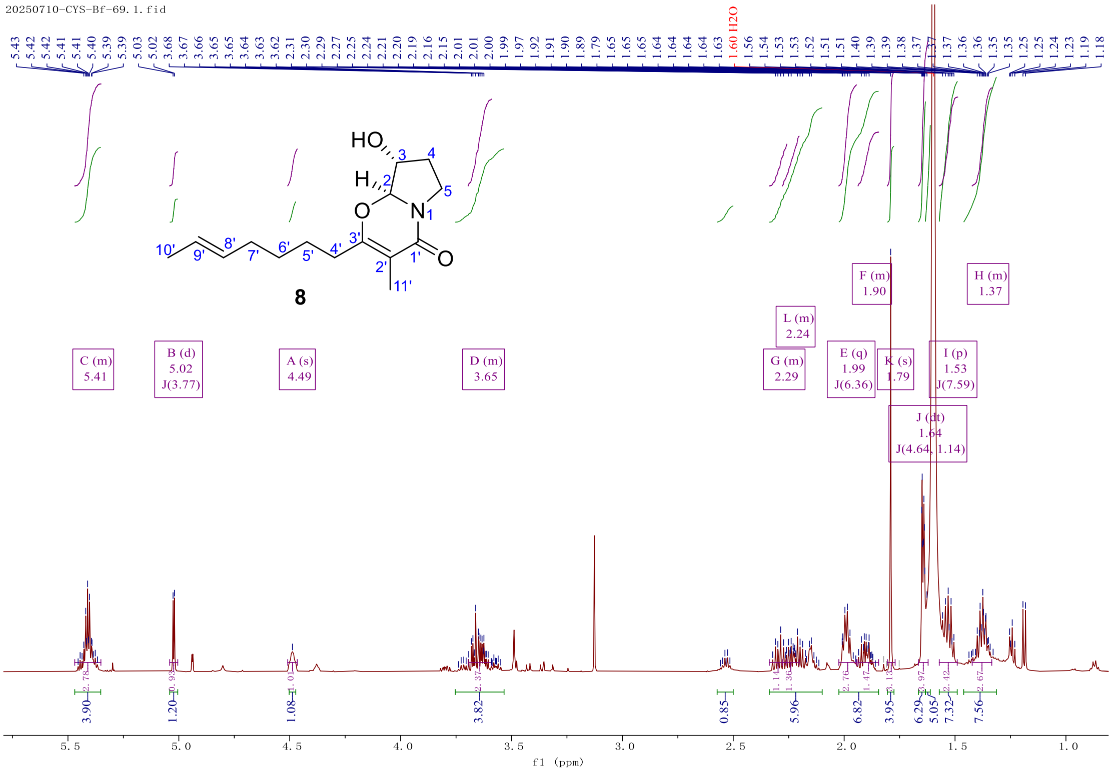

Figure S47 ^{13}C NMR spectrum of **8** (151 MHz, $CDCl_3$).

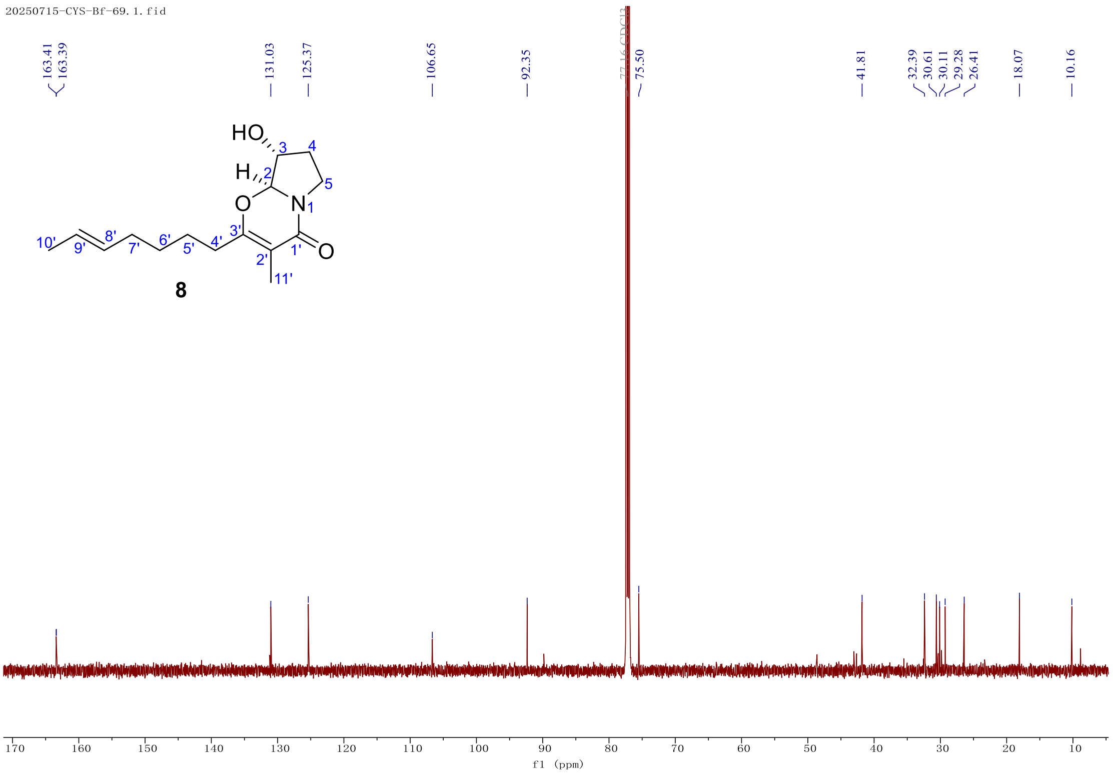

Figure S48 ^{1}H-^{1}H COSY spectrum of **8** (600 MHz, $CDCl_3$).

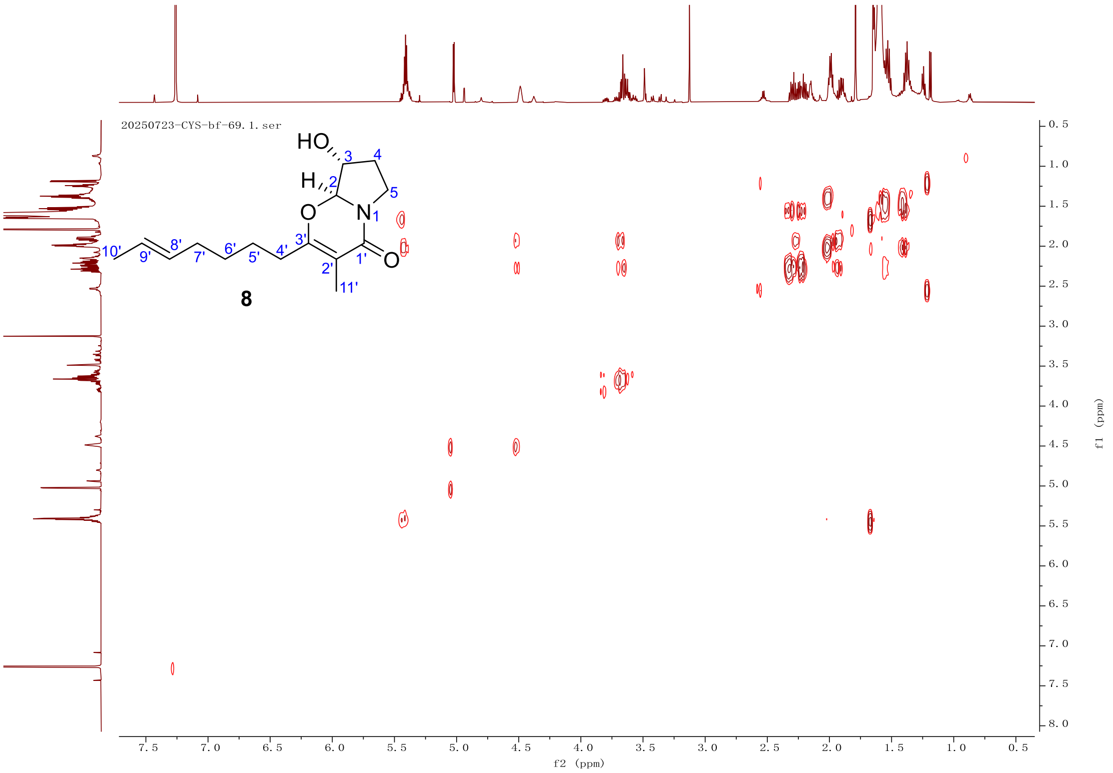

Figure S49 HSQC spectrum of **8** (600 MHz, $CDCl_3$).

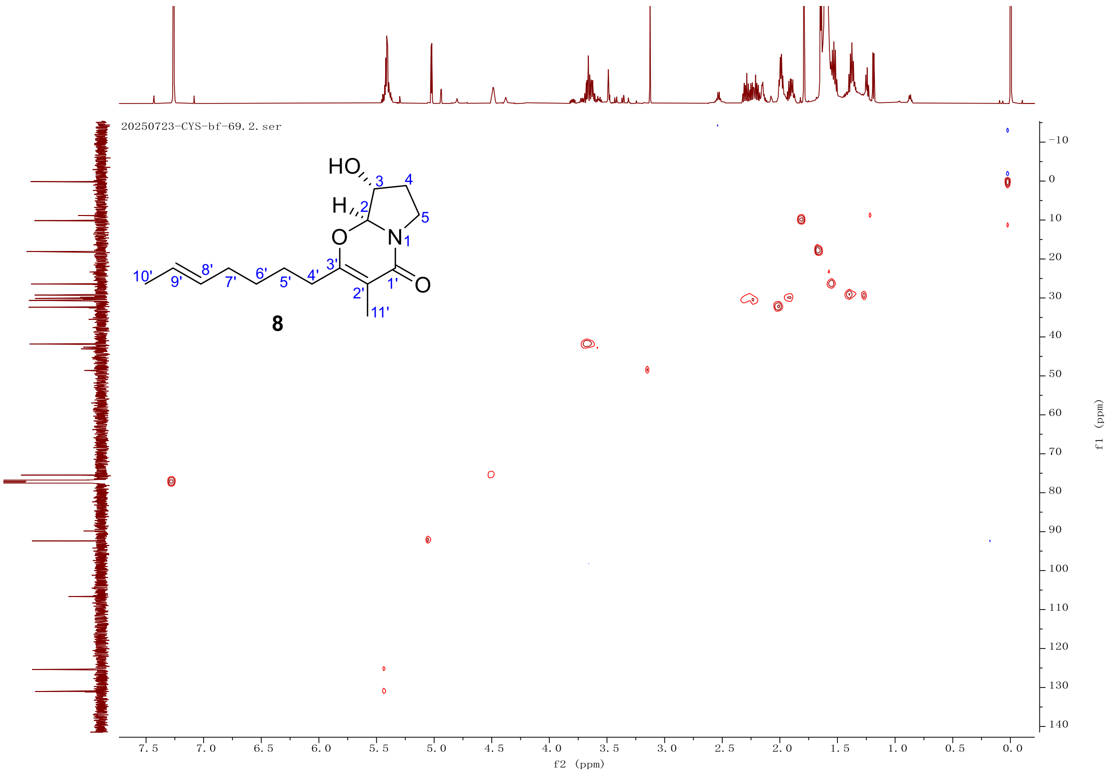

Figure S50 HMBC spectrum of **8** (600 MHz, $CDCl_3$).

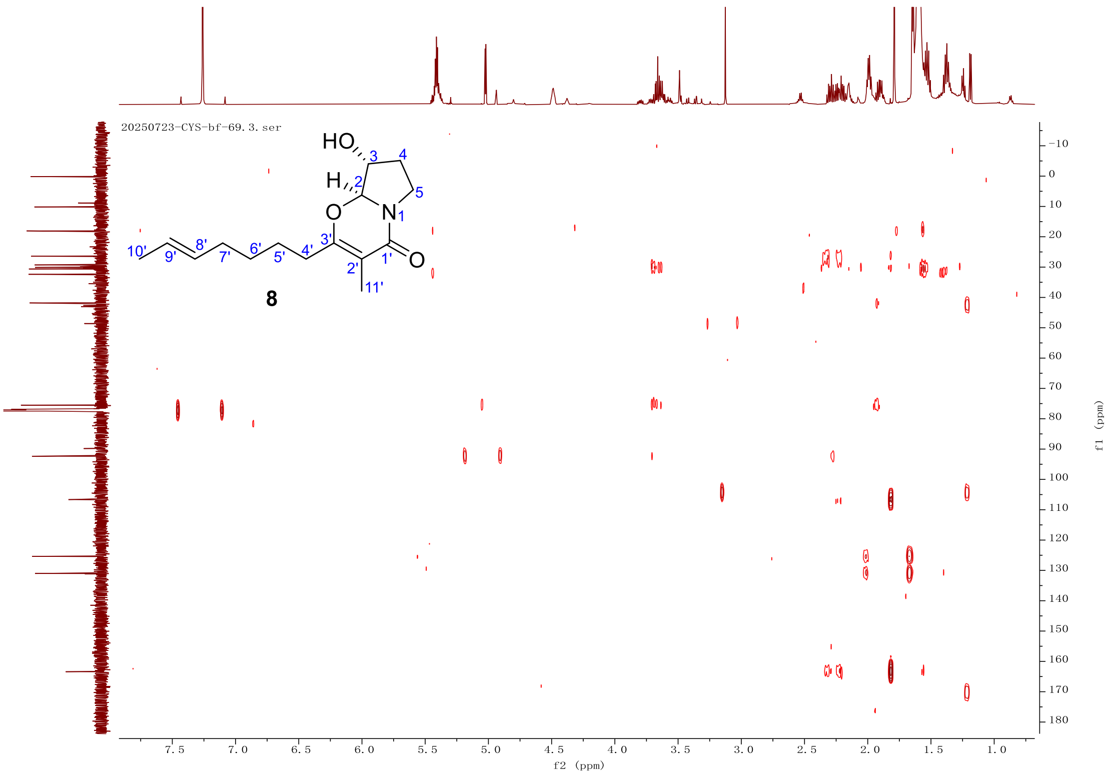

Figure S51 ECD spectrum of **8** in MeOH.

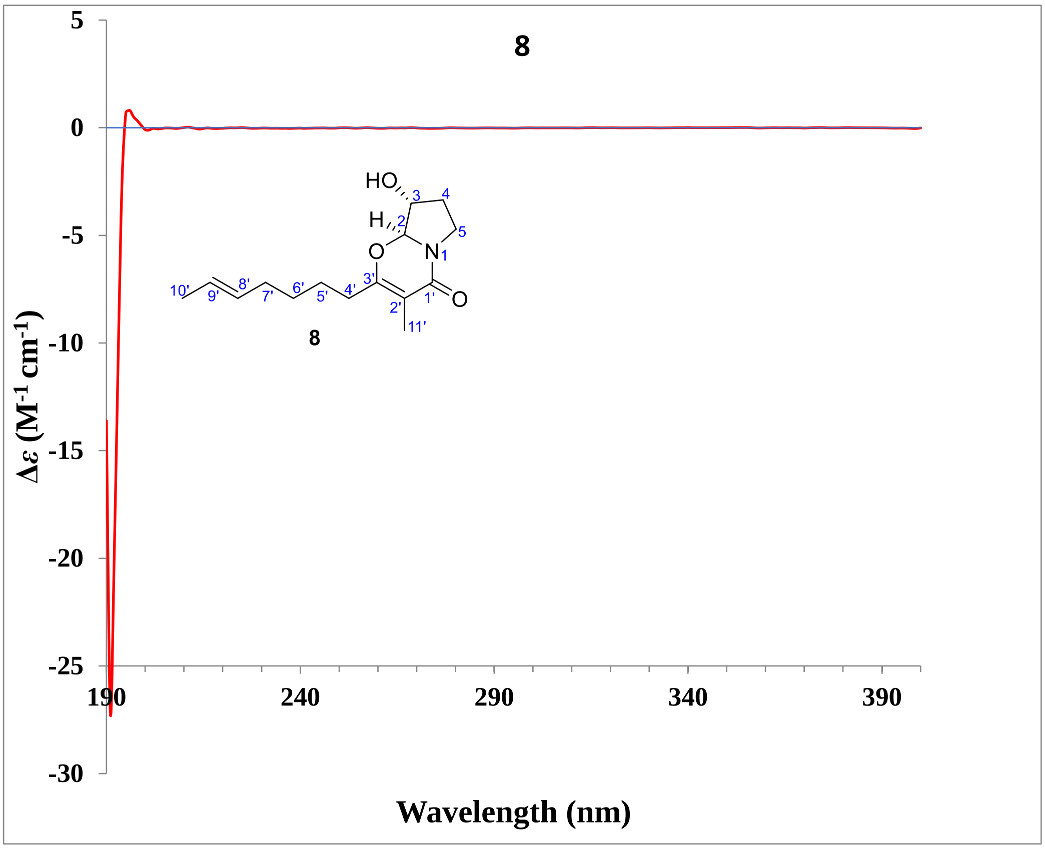

Figure S52 HRESIMS spectrum of **9**.

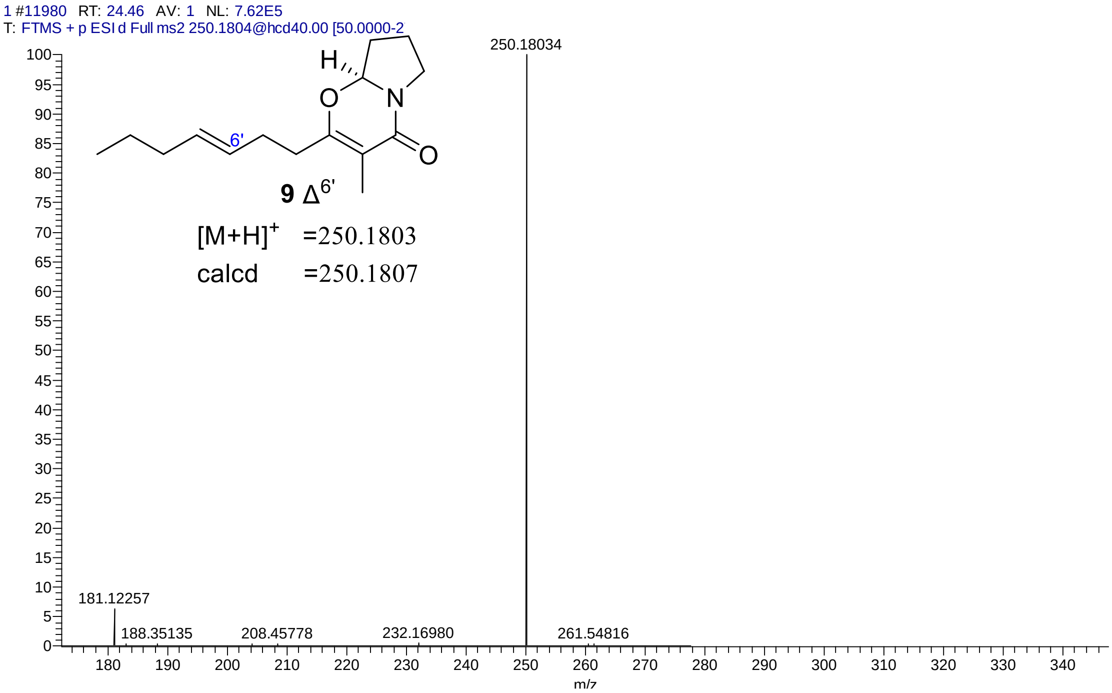

Figure S53 ^{1}H NMR spectrum of **9** (600 MHz, $CDCl_3$).

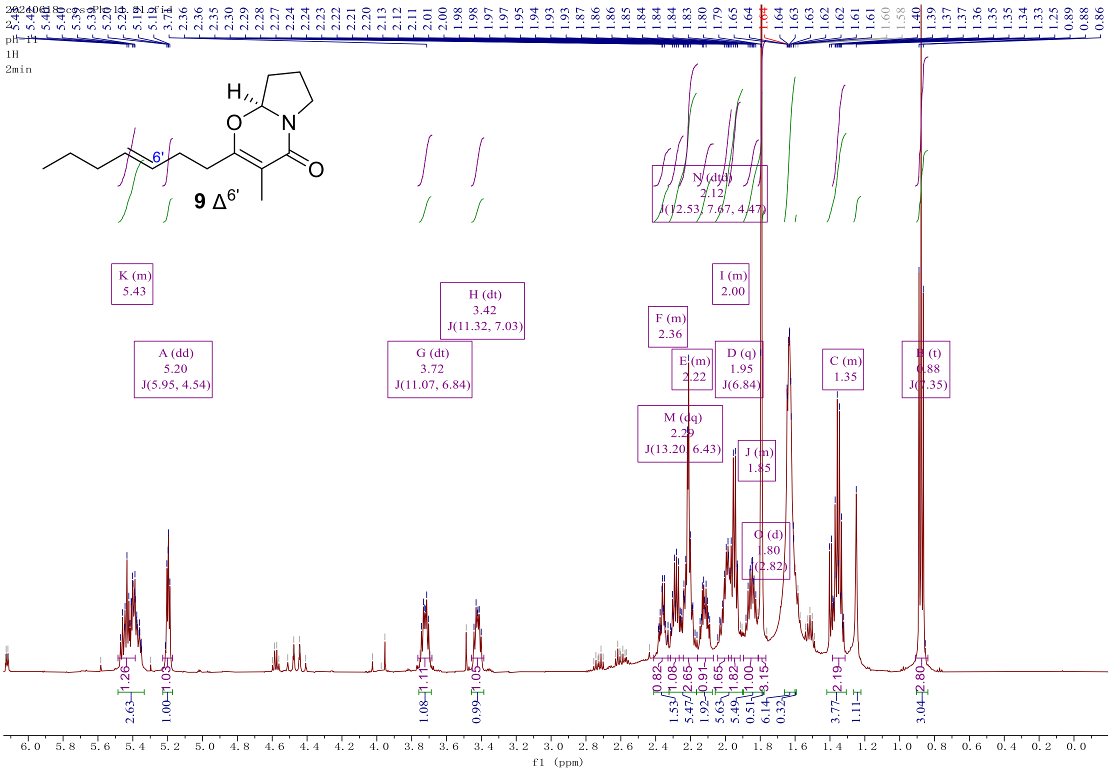

Figure S54 ^{13}C NMR spectrum of **9** (151 MHz, $CDCl_3$).

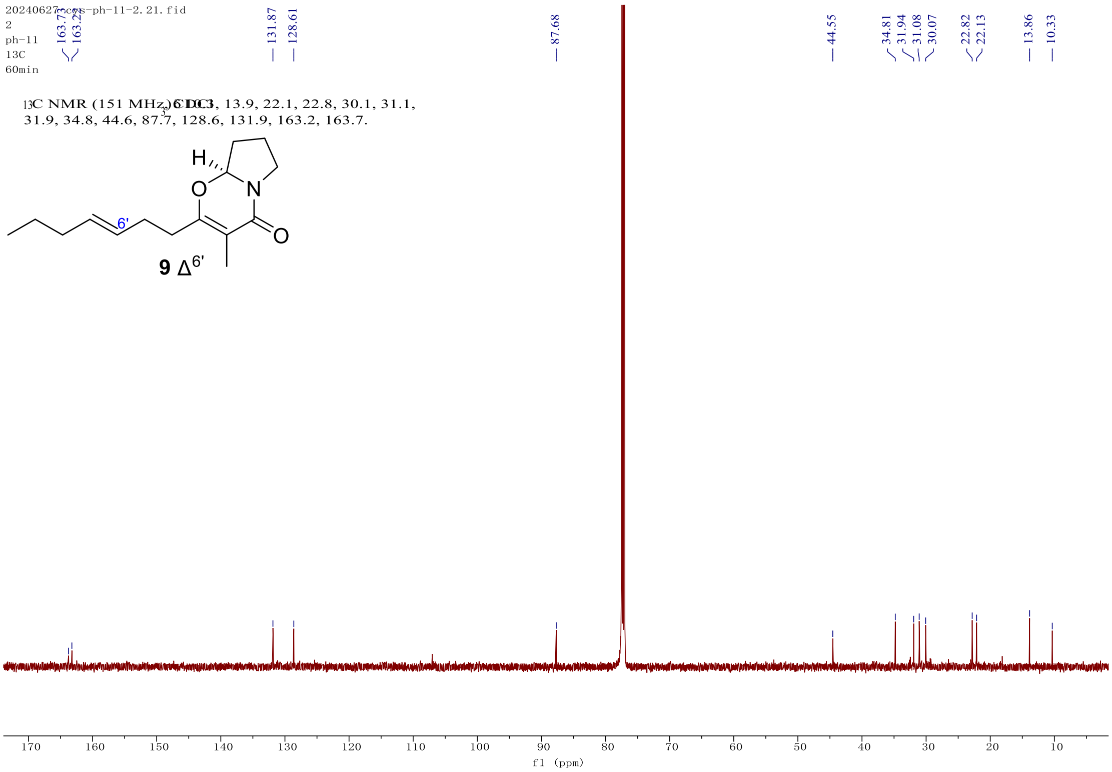

Figure S55 ^{1}H-^{1}H COSY spectrum of **9** (600 MHz, CDCl$_3$).

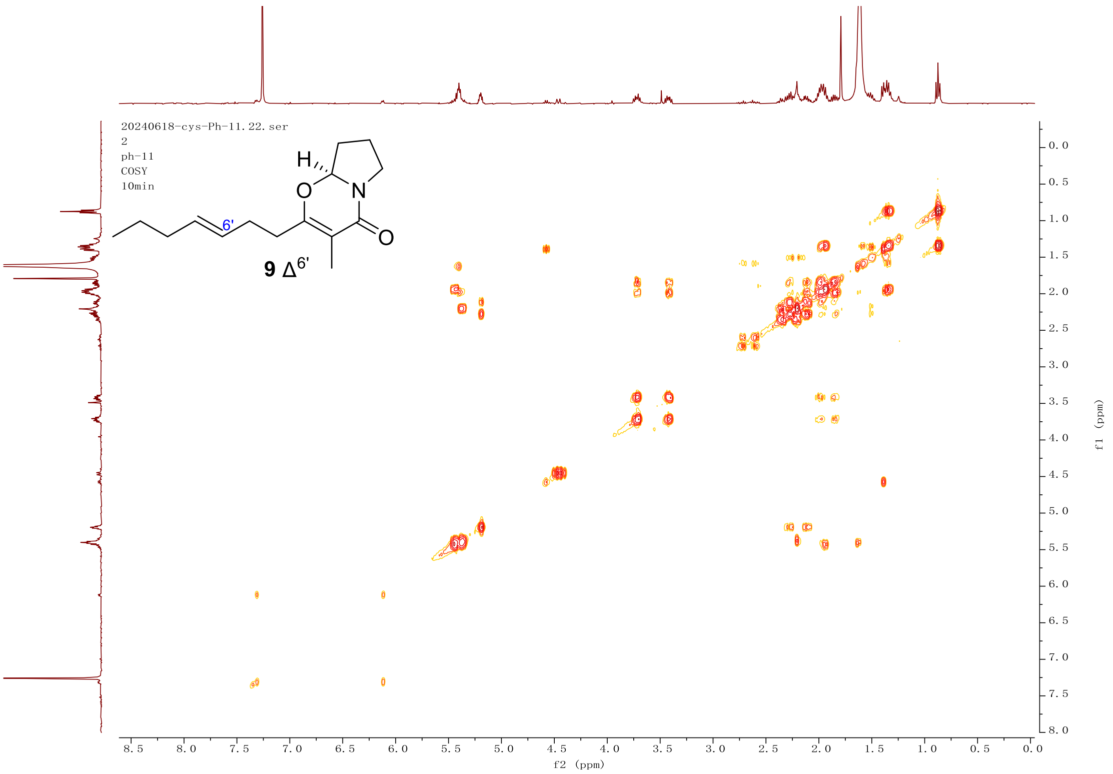

Figure S56 HSQC spectrum of **9** (600 MHz, CDCl$_3$).

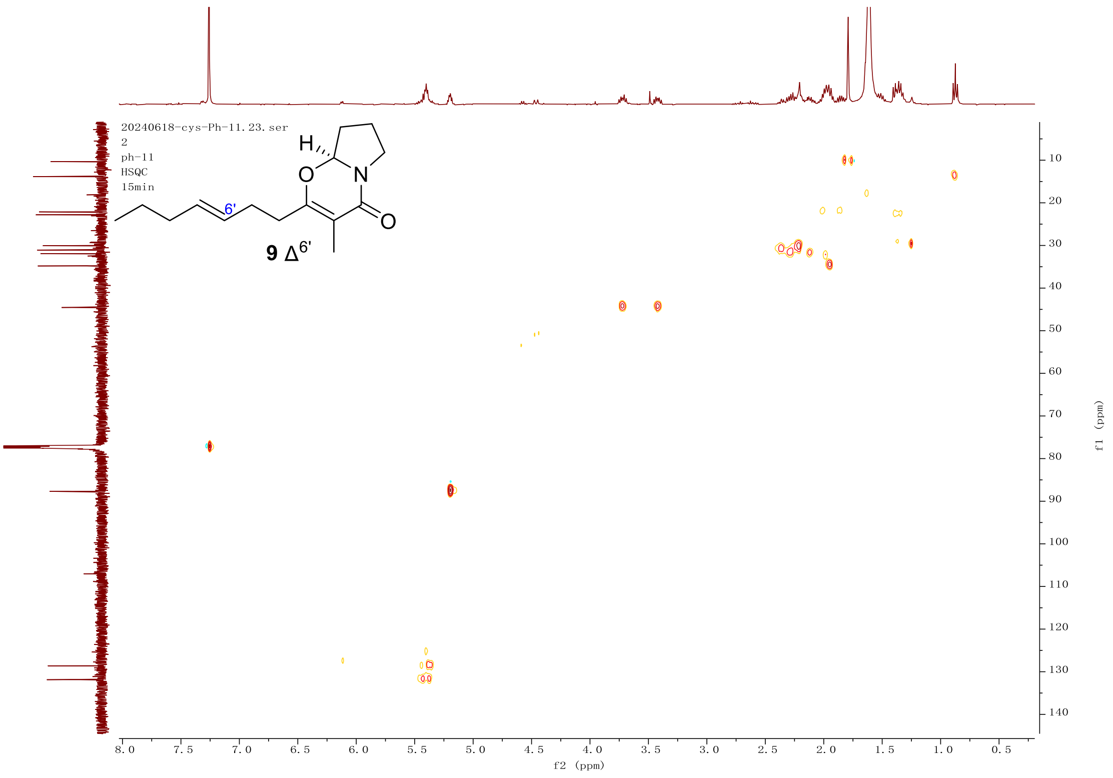

Figure S57 HMBC spectrum of **9** (600 MHz, $CDCl_3$).

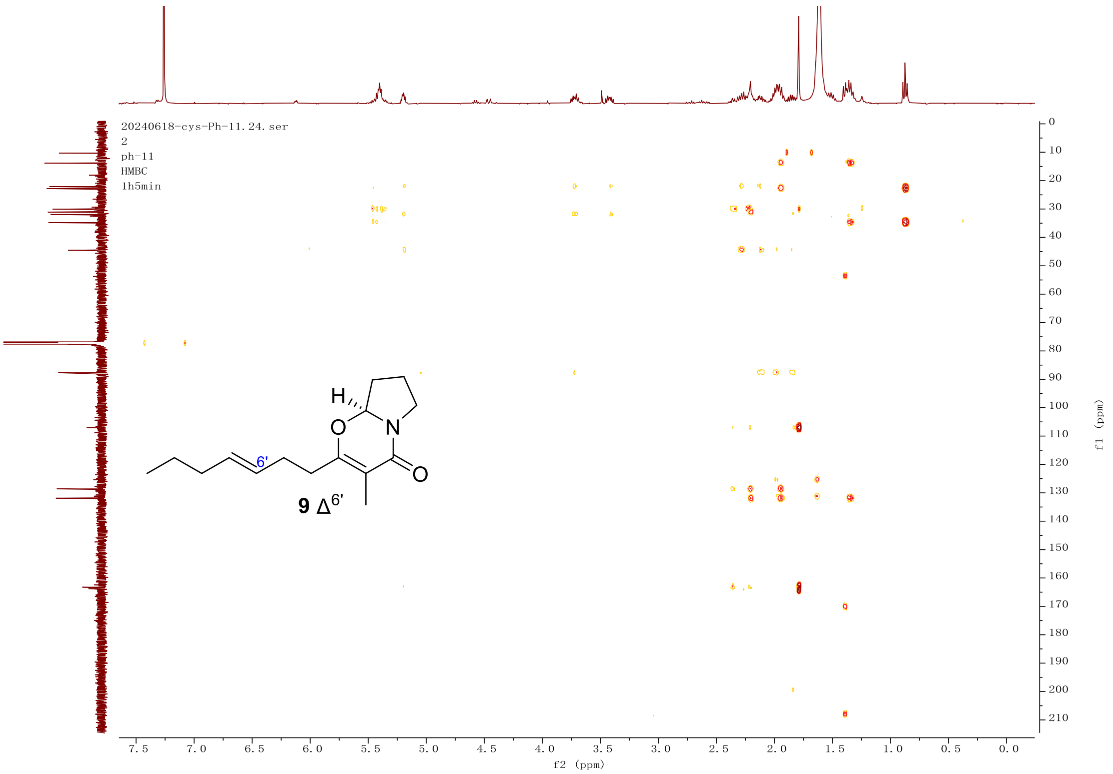

Figure S58 Experimental and calculated ECD spectra of **9** in MeOH.

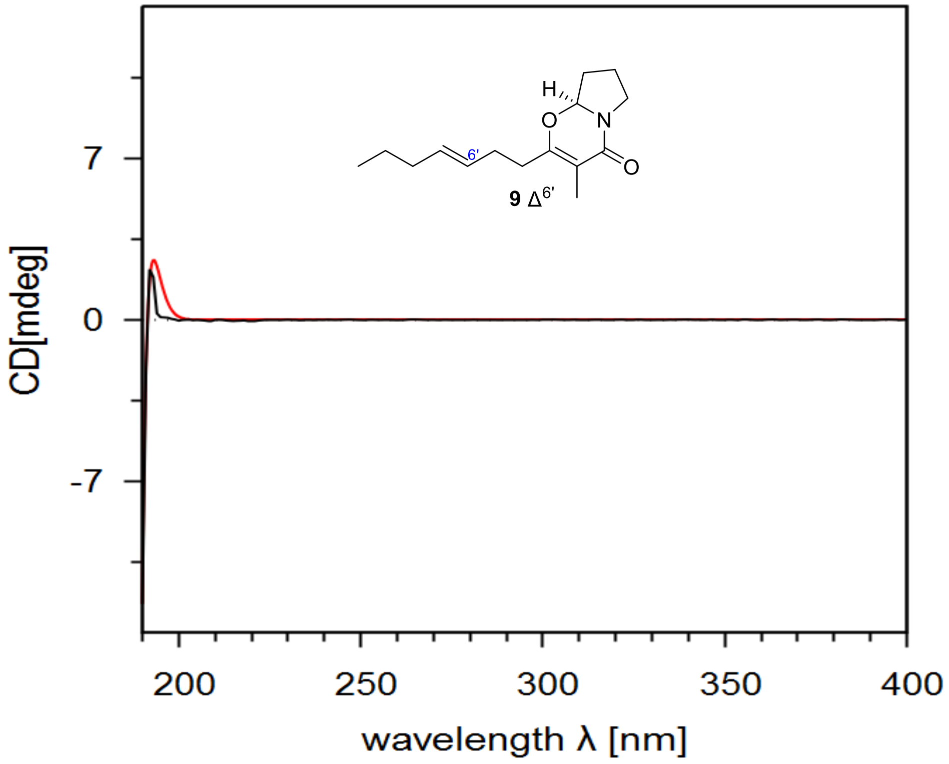

Figure S59 HRESIMS spectrum of **10**.

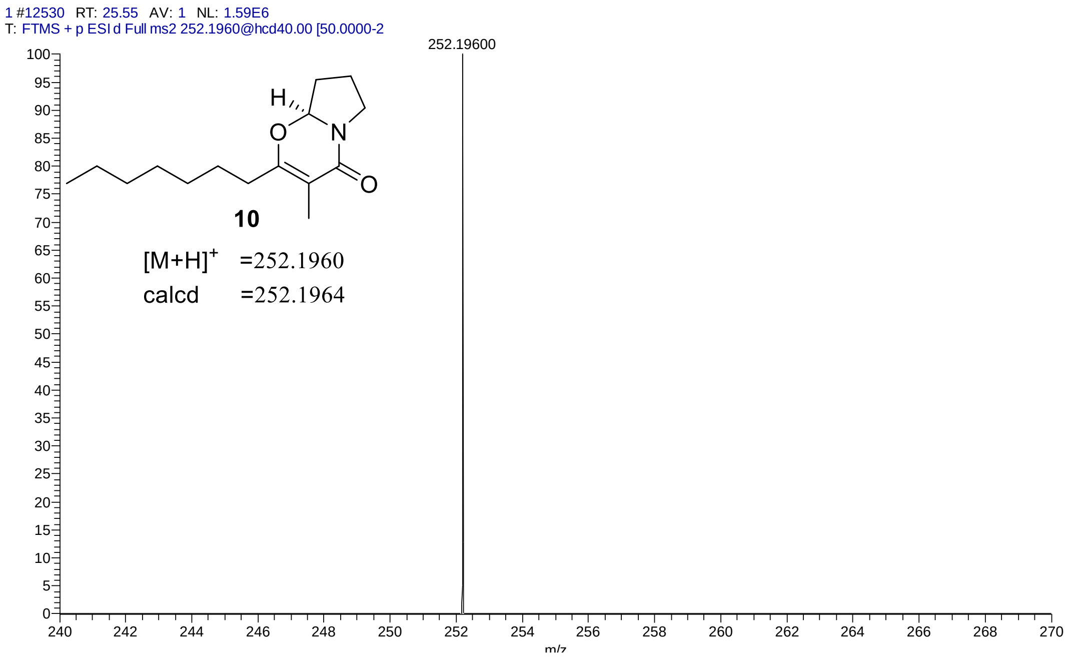

Figure S60 ^{1}H NMR spectrum of **10** (400 MHz, $CDCl_3$).

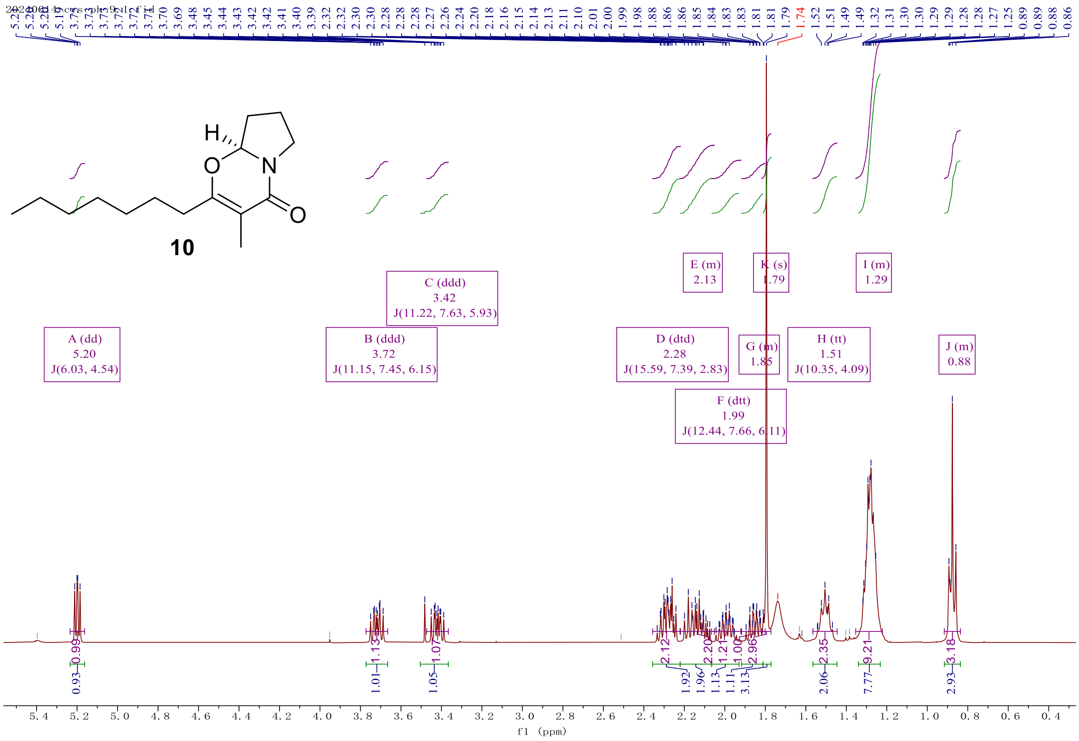

Figure S61 ^{13}C NMR spectrum of **10** (151 MHz, $CDCl_3$).

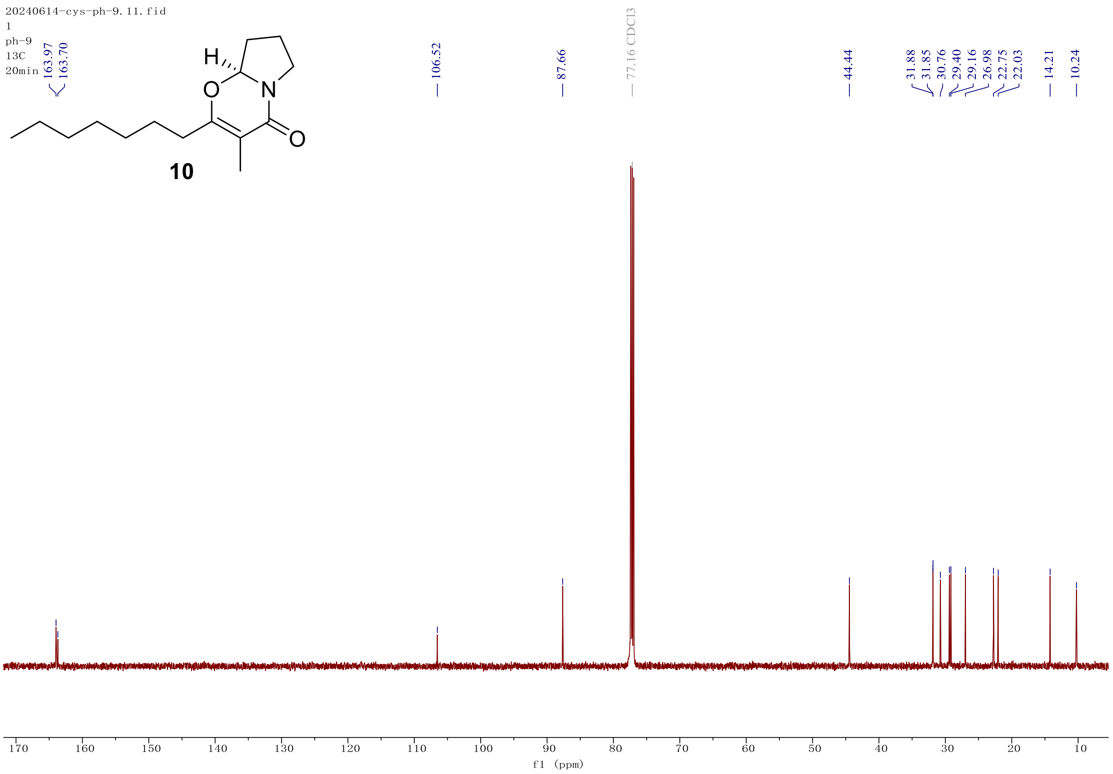

Figure S62 ^{1}H-^{1}H COSY spectrum of **10** (600 MHz, $CDCl_3$).

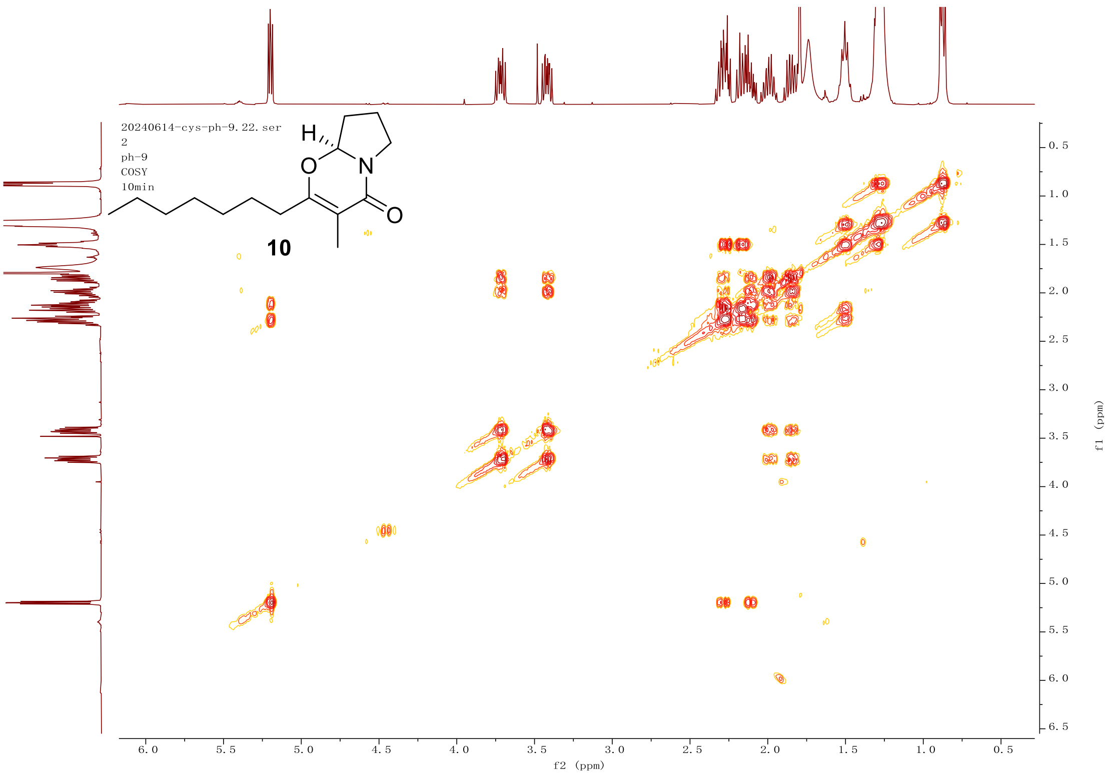

Figure S63 HSQC spectrum of **10** (600 MHz, $CDCl_3$).

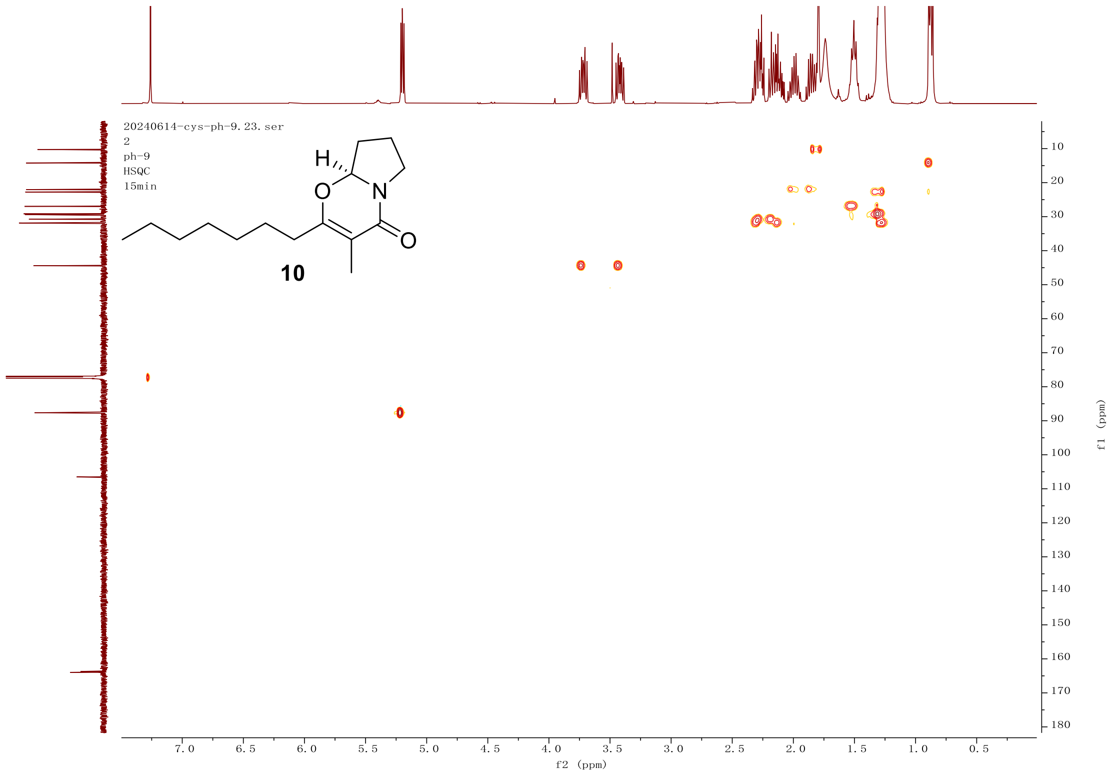

Figure S64 HMBC spectrum of **10** (600 MHz, $CDCl_3$).

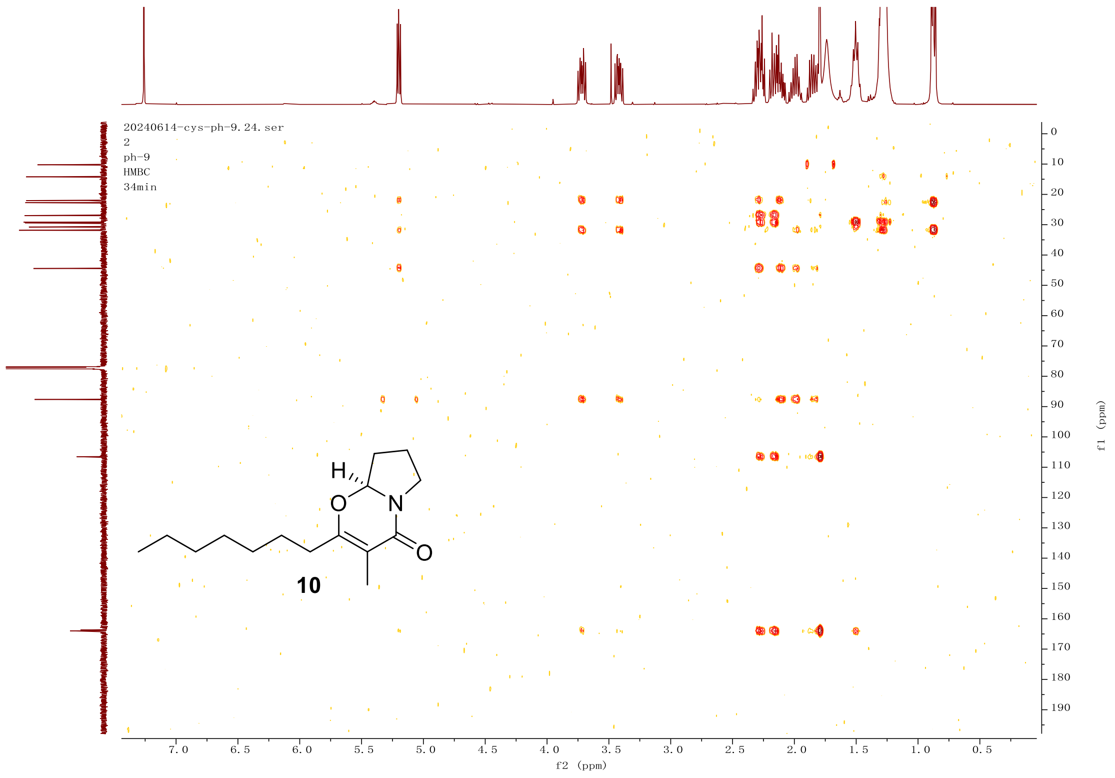

Figure S65 Experimental and calculated ECD spectra of **10** in MeOH.

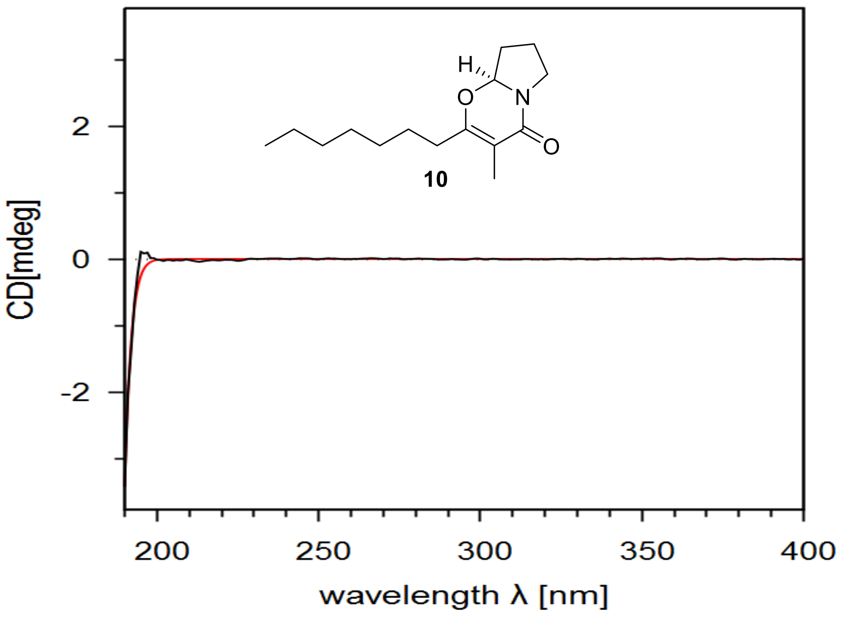

Figure S66 ECD spectrum of 11 in MeOH.

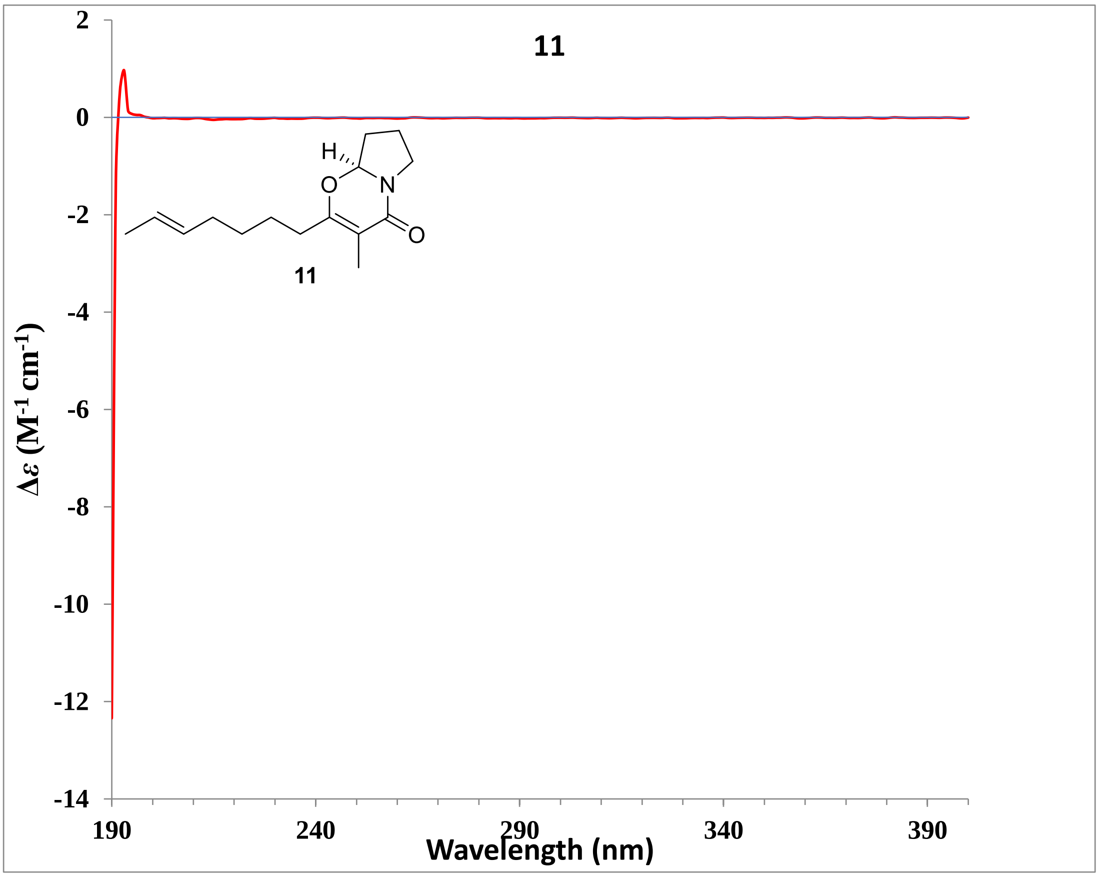

Figure S67 ^{1}H NMR spectrum of **11** (400 MHz, $CDCl_3$).

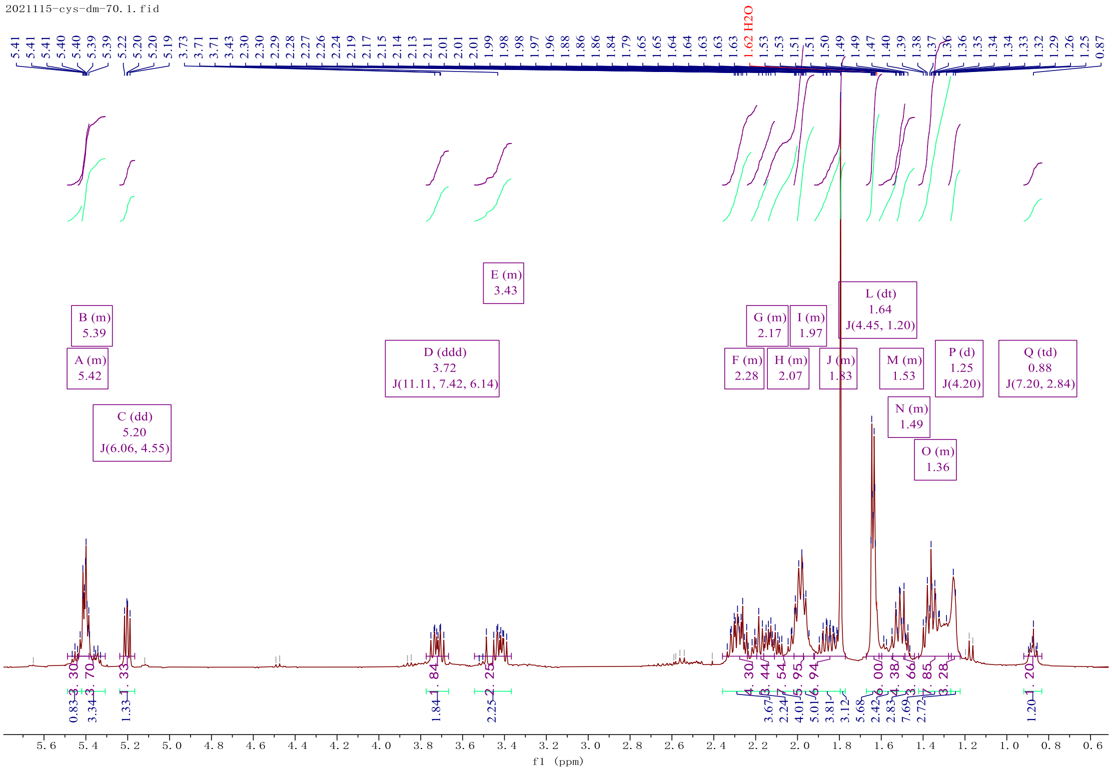

Figure S68 ^{13}C NMR spectrum of **11** (101 MHz, $CDCl_3$).

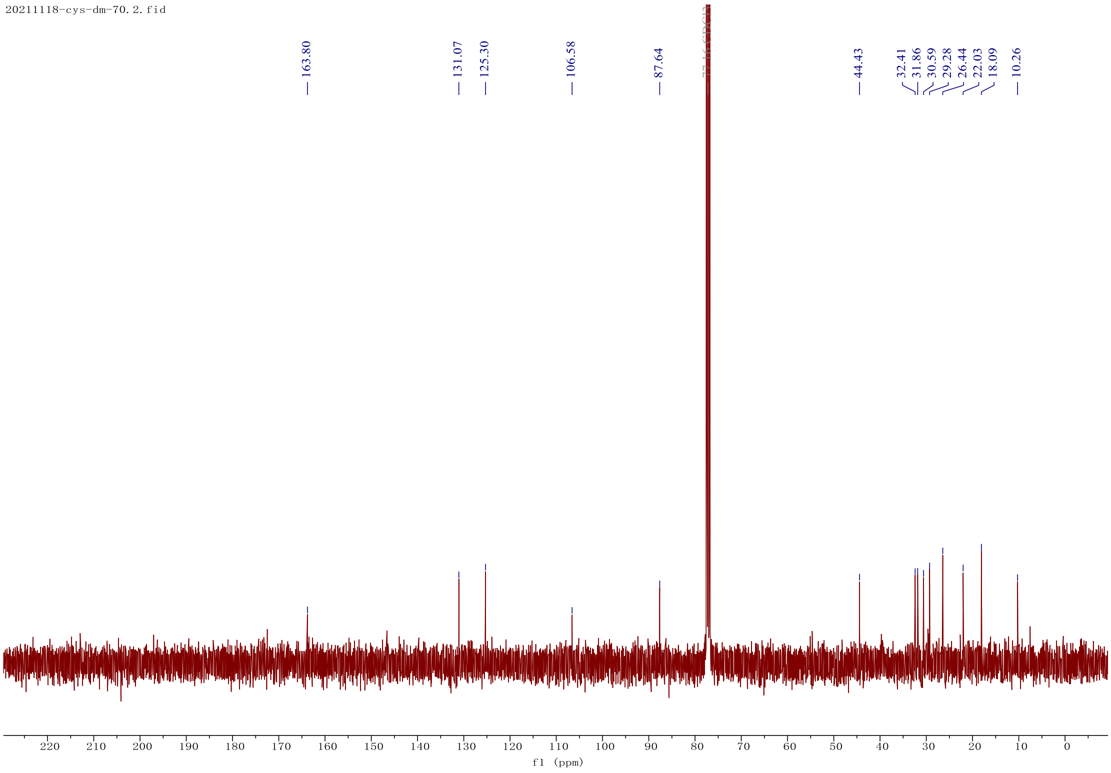

Figure S69 ^{1}H NMR spectrum of **12** (400 MHz, $CDCl_3$).

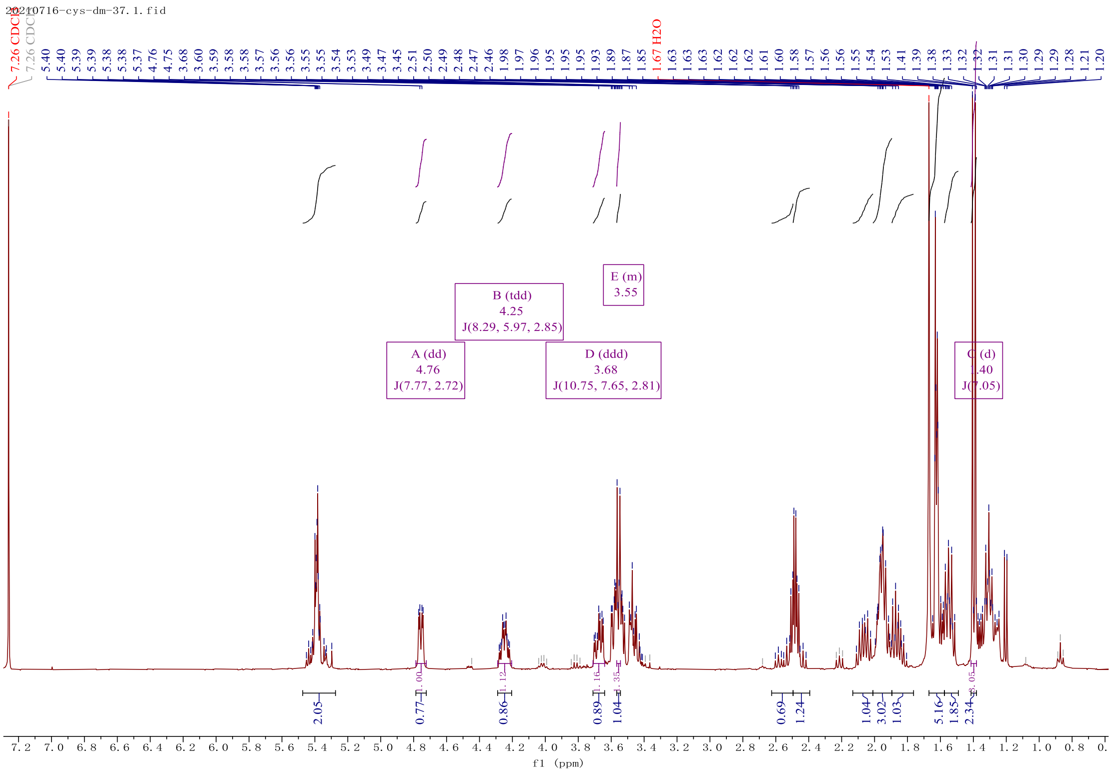

Figure S70 ^{13}C NMR spectrum of **12** (101 MHz, $CDCl_3$).

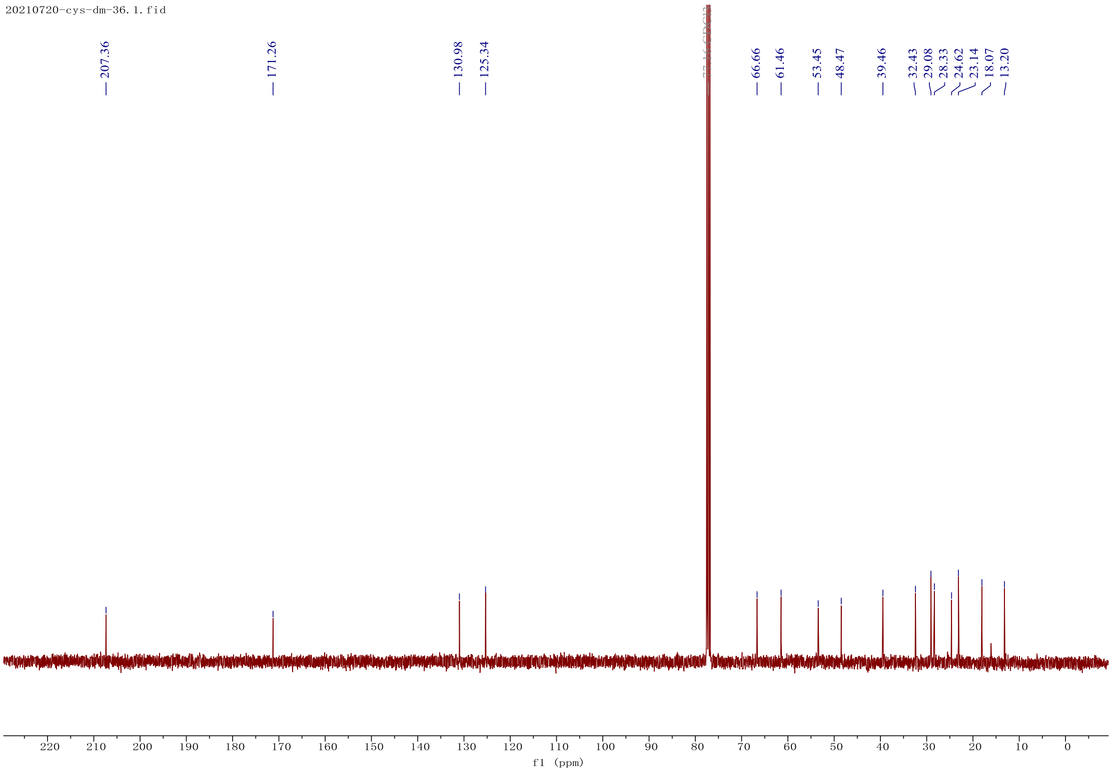

Figure S71 ^{1}H NMR spectrum of **13** (400 MHz, $CDCl_3$).

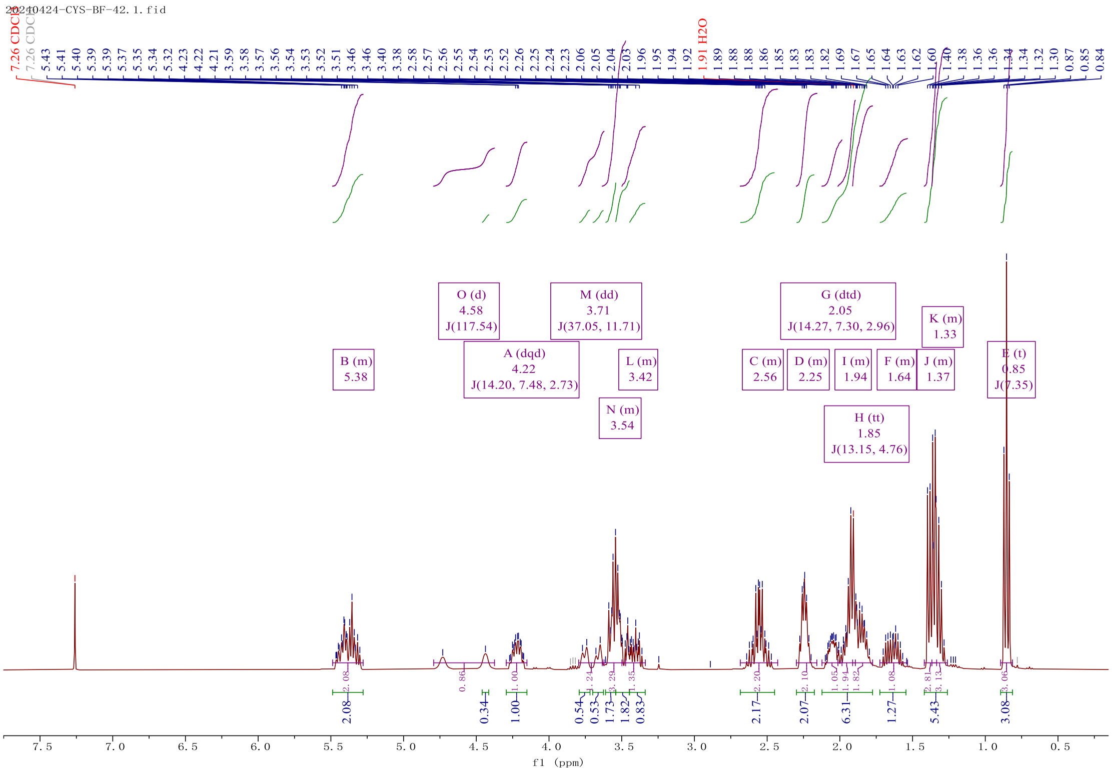

Figure S72 ^{13}C NMR spectrum of **13** (151 MHz, $CDCl_3$).

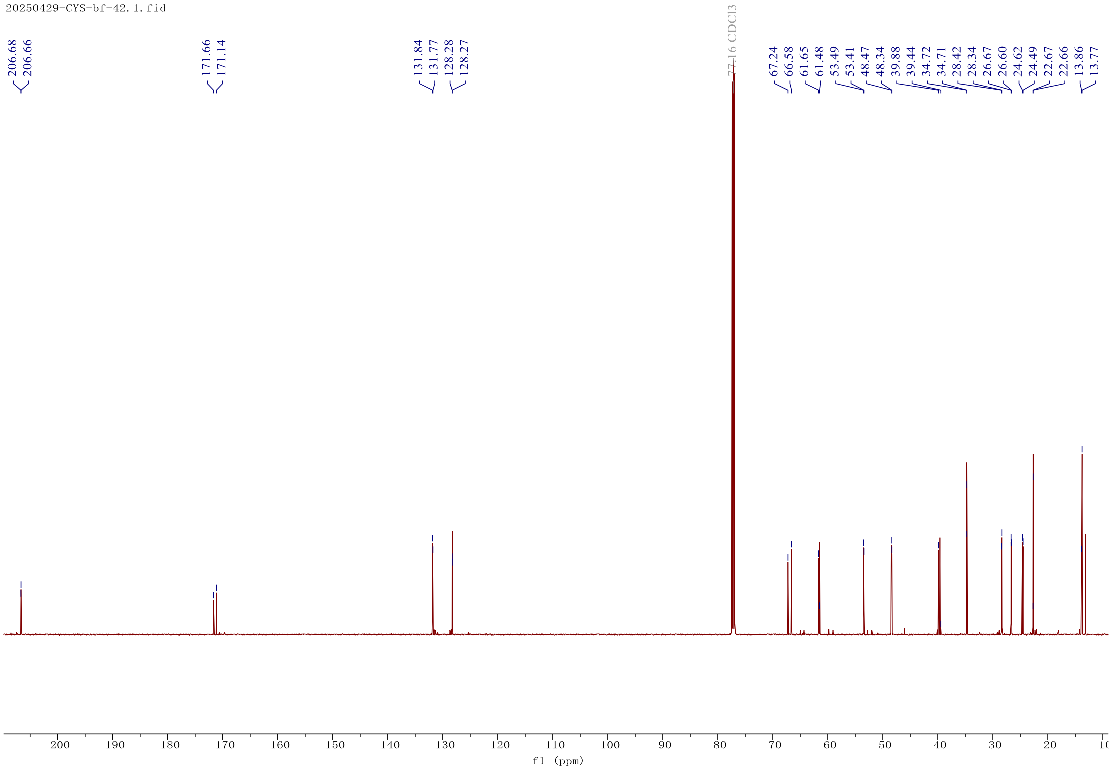

Figure S73 ^{1}H NMR spectrum of **14** (400 MHz, $CDCl_3$).

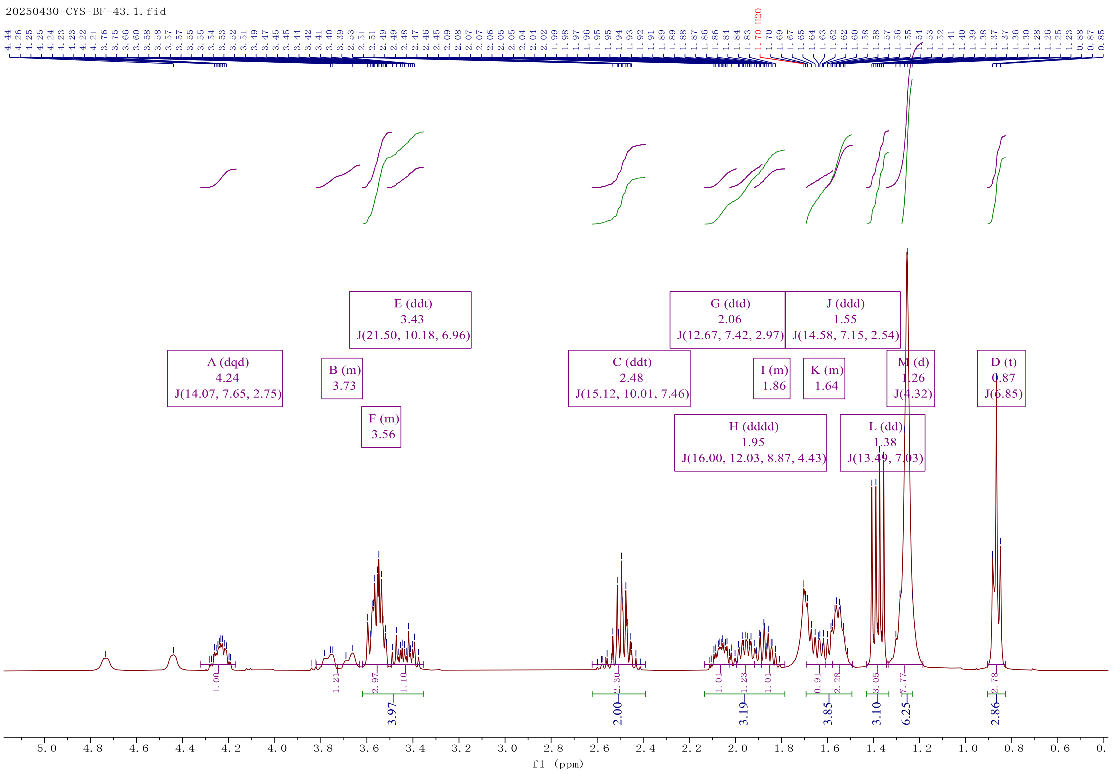

Figure S74 ^{13}C NMR spectrum of **14** (151 MHz, $CDCl_3$).

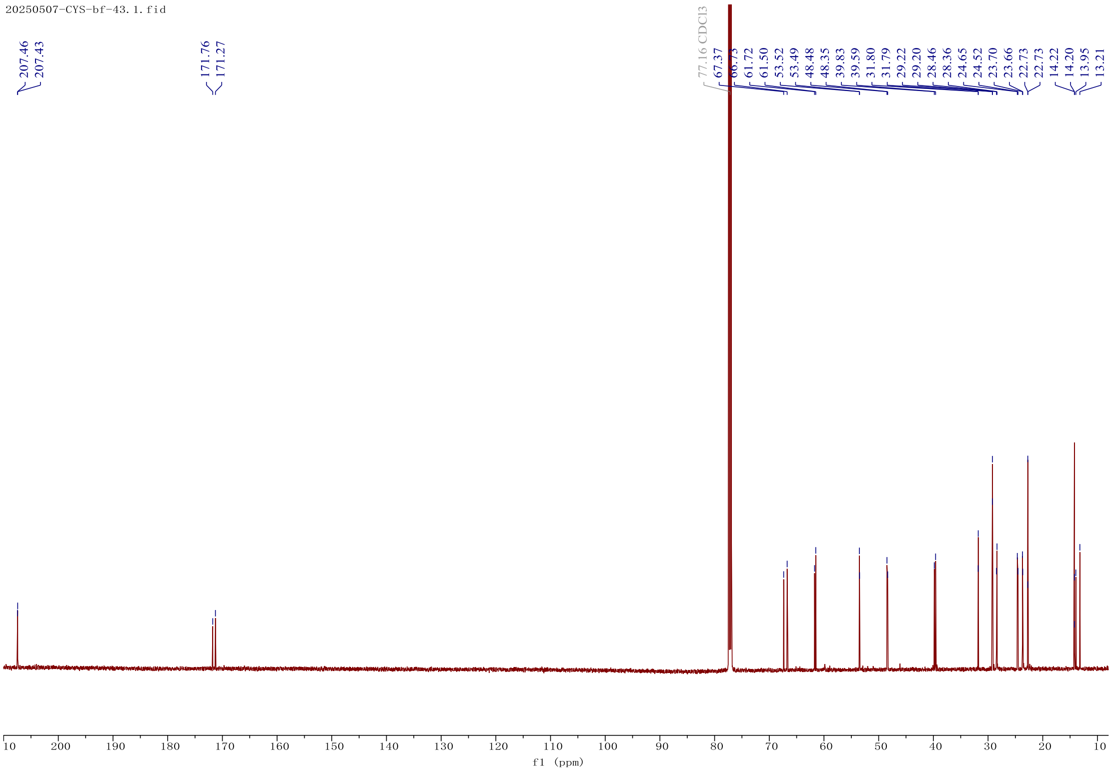

Figure S75 ^{1}H NMR spectrum of **15** (400 MHz, $CDCl_3$).

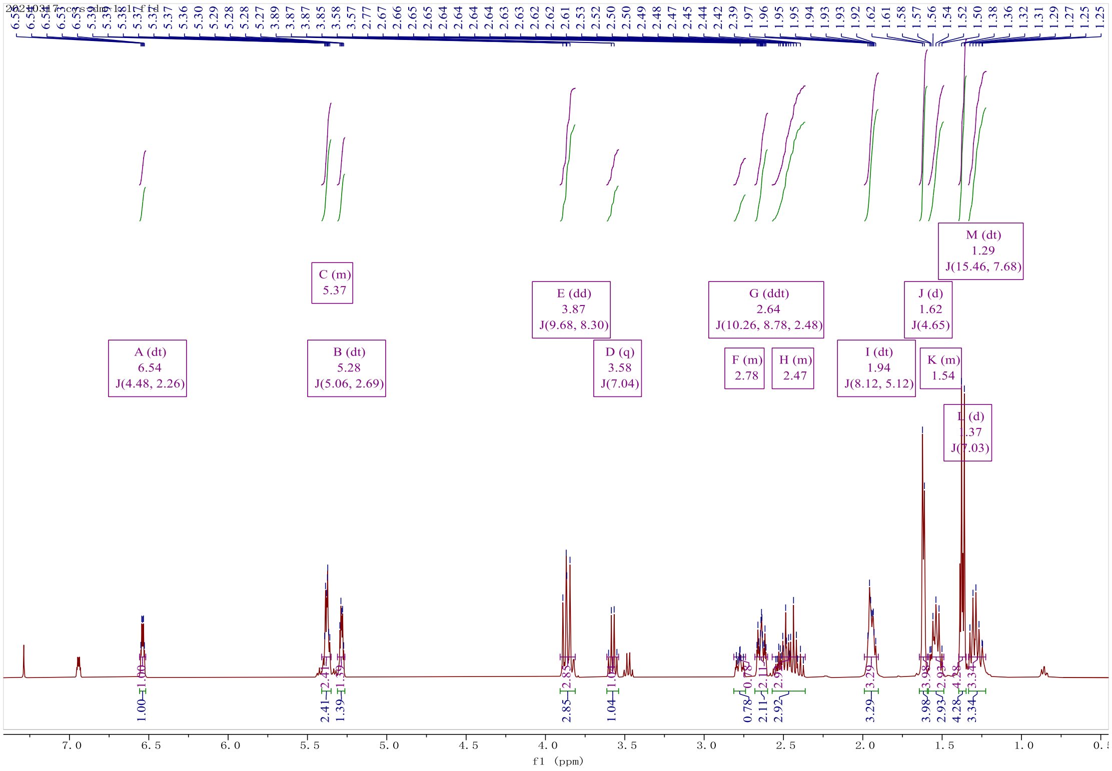

Figure S76 ^{13}C NMR spectrum of **15** (101 MHz, $CDCl_3$).

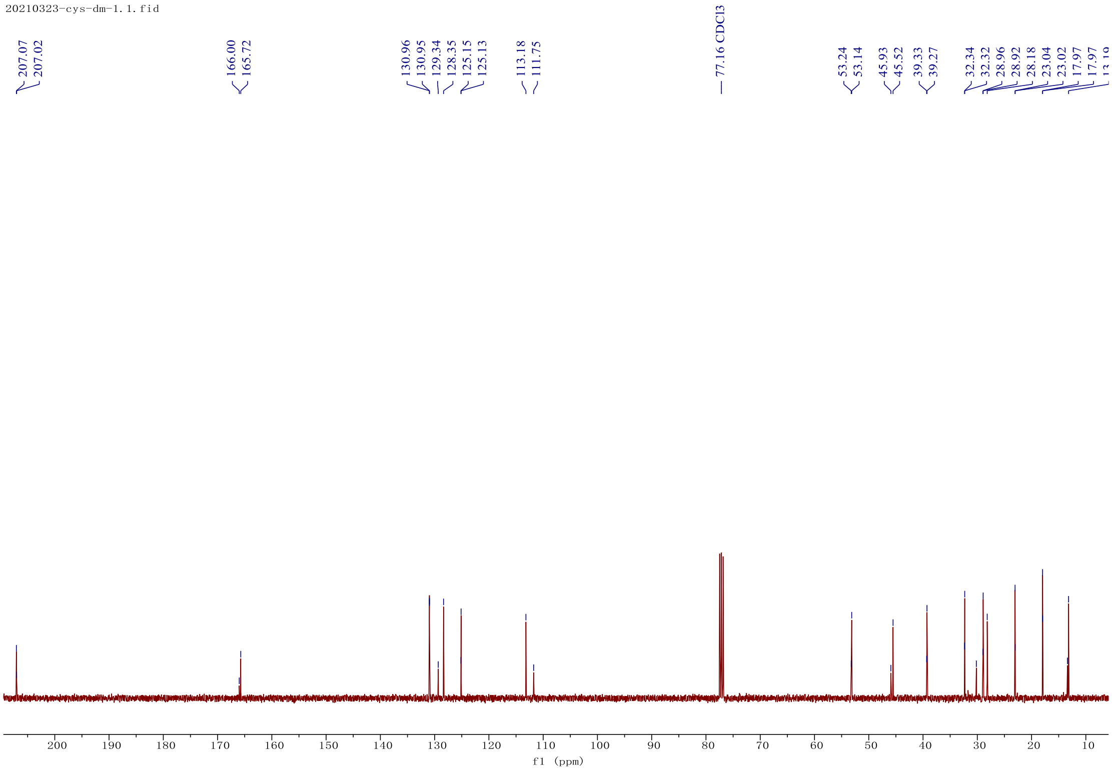

Figure S77 Cell viability of L6 cells treated with compounds **1−10** (n = 5). The data are presented as the mean ± SD. * $p < 0.05$ vs the DMSO control.

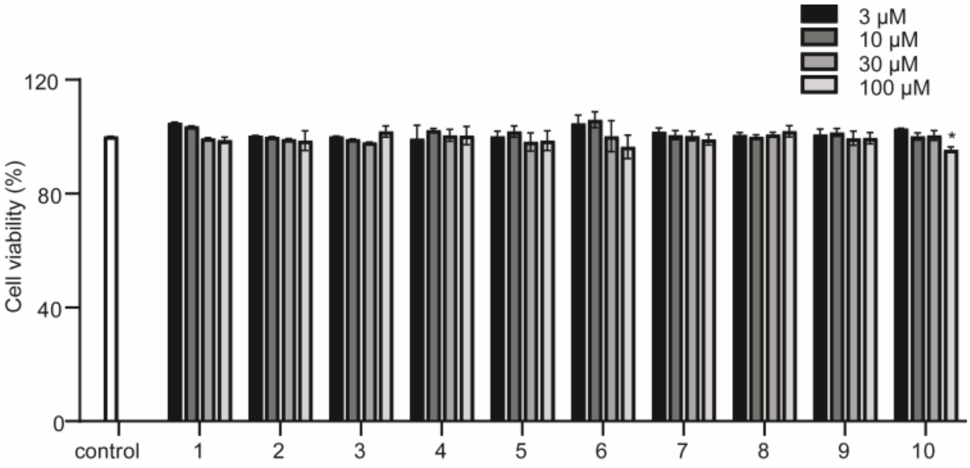

Supplement: Supplementary file 1 [file marinedrugs-23-00455-s001.zip › marinedrugs-3986912-supplementary.pdf]
